# Plastic Pores for Switchable and Optimized Adsorption Behaviors

**DOI:** 10.1021/acscentsci.4c02155

**Published:** 2025-03-13

**Authors:** Xue-Wen Zhang, Rong-Hua Wang, Jie-Peng Zhang, Xiao-Ming Chen

**Affiliations:** MOE Key Laboratory of Bioinorganic and Synthetic Chemistry, GBRCE for Functional Molecular Engineering, School of Chemistry, IGCME, Sun Yat-Sen University, Guangzhou 510275, China

## Abstract

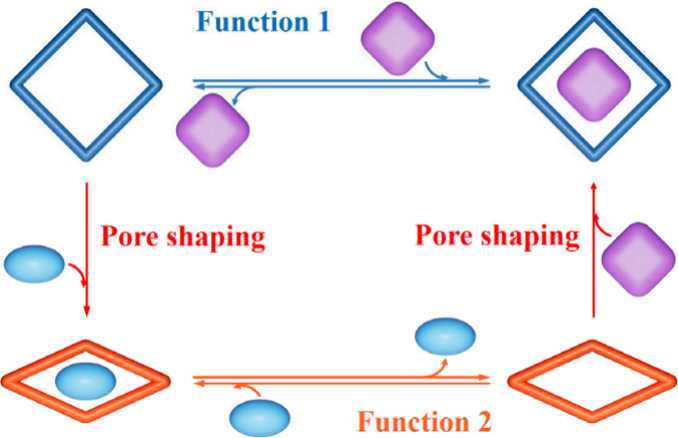

Similar to conventional solids, porous materials have demonstrated rigid and flexible behaviors. Here, we show that flexible pores can be not just elastic but also plastic. By variation of the hydrogen-bonding ability and steric hindrance of ligand side groups, the energy difference and barrier between metastable states of a porous framework are fine-tuned to enable the plastic behavior. All metastable pore structures can transform to the target ones in atmospheres of the target guests with sufficiently high pressures, and all shaped pores can remain unchanged after guest removal, resulting in optimized host–guest recognitions for the target guests. Up to a 6-fold increase of adsorption selectivity and 9-fold increase of purification productivity for CO_2_ capture and coalmine CH_4_ upgrading, and even inversion of CO_2_/C_2_H_2_ selectivity, have been achieved by reversible pore-shaping of a single plastic-pore adsorbent. The realization of plastic pores creates an opportunity for on-demand switching of adsorption and separation functions with optimized performances.

## Abstract 2

While porous materials possess rigid or flexible/elastic pores, a plastic pore is realized for the first time, which can be shaped by target guest molecules to meet needs of different applications.

## Introduction

As typical mechanical properties of solids, rigidity and flexibility refer to the abilities to resist or allow shape deformation under external stress, respectively.^1^ Moreover, flexible solids can undergo either elastic or plastic deformation, which is reversible or nonreversible (permanent) upon releasing the stress, respectively.^2^ Rigid, elastic, and plastic solids are useful in different situations. For example, elastics can buffer, store, and release mechanical energy, while plastics can be easily shaped to meet specific requirements.

**Figure 1:**
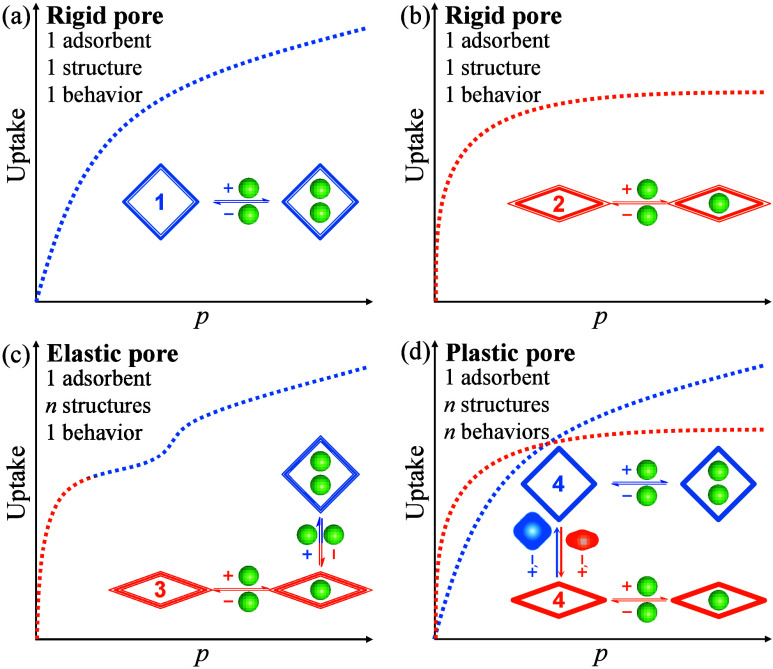
Adsorption and structural transformation behaviors of rigid/flexible pores. (a,b) Rigid pore (one rigid-pore adsorbent exhibits one behavior; multiple rigid adsorbents are required to show multiple behaviors), (c) elastic pore (one elastic-pore adsorbent exhibits one behavior, although the entire process shows multiple structures), and (d) plastic pore (one plastic-pore adsorbent exhibits multiple behaviors, because it possesses multiple structures after pore shaping by specific guests, as illustrated by the solid blue square and orange rhombus). Ideally *n* ≫ 1, but *n* = 2 is used here for clarity. Four adsorbent materials **1** ∼ **4** (two rigid, one elastic, and one plastic in different line styles, respectively) with two types of structures (square and rhombus) and three types of adsorption/transformation behaviors (each is represented by one isotherm shape) for one adsorbate (small green spheres) are shown.

Porous materials are originally considered to be rigid (Figures 1a,b and S1).^3,4^ With the advent of molecule-based crystalline adsorbents, particularly porous coordination polymers or metal–organic frameworks, flexibility of porous frameworks under chemical stress (i.e., the change of guest loading) has attracted great attention (Figures 1c and S1).^5−11^ As the result of reversible guest-induced structural transformations, elastic pores exhibit abnormal isotherm shapes useful for improving working capacity and lowering energy consumption in storage/separation applications.^12,13^

Regardless of being rigid or elastic, an adsorbent can present only one kind of adsorption behavior for a given guest at a given condition (Figures 1a–c and S1). To change/optimize adsorption behavior, synthesizing new adsorbents is necessary, but precise elucidation of structure–property relationships and construction of target porous structures are long-lasting challenges.^14−17^ In principle, plastic pores can switch pore size/shape to fit various guest molecules and attain optimized host–guest recognition for corresponding adsorption applications, which combine the advantages of both rigid and elastic pores (Figures 1d and S1).

**Figure 2:**
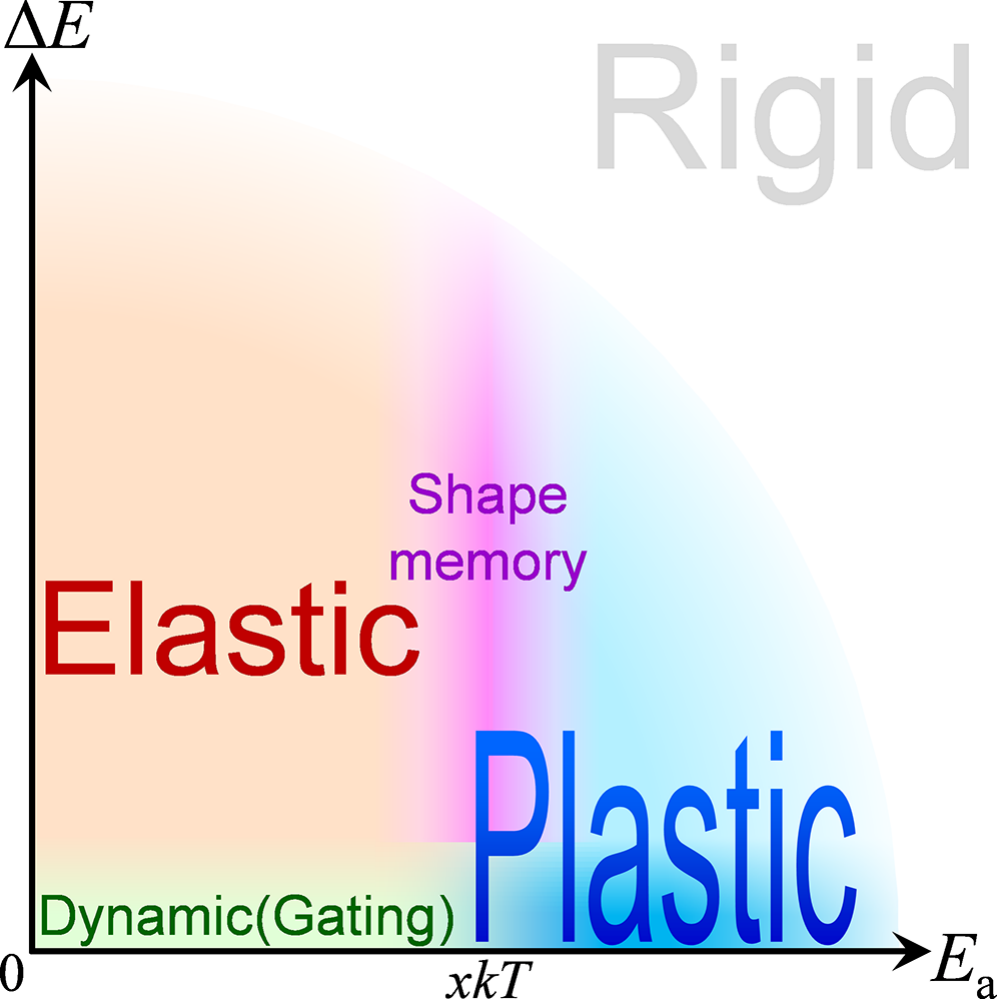
Thermodynamic and kinetic characteristics of different types of adsorbent flexibility. *xkT* refers to the system energy fluctuation, where *x* is related to the spatial range of the independent structural transformation (e.g., the domain size). When Δ*E* and/or *E*_a_ are too large, there is no structural transformation (rigid). Note that the shape-memory pore is a subset of plastic pore. However, the smaller Δ*E* makes it easier to realize/utilize plastic pore, which contrasts with the requirement for the shape-memory pore.

It is well-known that flexibility relies on the presence of multiple metastable states with similar energies, i.e., small energy differences (Δ*E*). The important role of the energy barrier (*E*_a_) for structural transformation has also been realized (Figure 2 and Table S1).^18−20^ Typically, multiple metastable states are separated with negligibly low energy barriers (*E*_a_ < *xkT*) so that they spontaneously transform to the most stable state defined by the guest environment, resulting in elastic behaviors.^21−23^ To construct plastic pores, multiple metastable pore structures should have not only distinct guest recognition abilities but also suitably high energy barriers. However, energy differences and barriers are extremely difficult to tune precisely. Only a few flexible adsorbents possess suitable energy barriers,^19,24−30^ but they have demonstrated the shape-memory pore behavior rather than the plastic-pore behavior.

**Figure 3:**
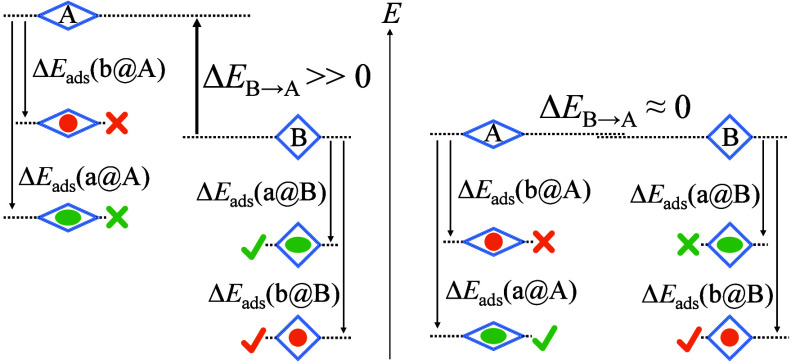
Effect of the structural transformation energy on the guest recognition ability of plastic pores. Two pore states **A** and **B** (Δ*E*_**B**→**A**_**≥ 0)** match guests **a** and **b** better, respectively. Regardless of the magnitude of Δ*E*_**B**→**A**_, adsorption of **b** always gives **b**@**B**. To accomplish pore shaping, the energy of **a**@**A** should be lower than that of **a**@**B**, i.e., Δ*E*_ads_(**a**@**A**) + Δ*E*_**B**→**A**_ < Δ*E*_ads_(**a**@**B**) + 0 or Δ*E*_ads_(**a**@**B**) – Δ*E*_ads_(**a**@**A**) > Δ*E*_**B**→**A**_. When Δ*E*_**B**→**A**_ is large, a small difference between Δ*E*_ads_(**a**@**B**) and Δ*E*_ads_(**a**@**A**) is not enough. When Δ*E*_**B**→**A**_ = 0, simply Δ*E*_ads_(**a**_@_**B**) > Δ*E*_ads_(**a**@**A**) is enough.

In addition to the lack of suitable pore structures and guest recognition abilities (most examples involve a nonporous phase as the stable state, which cannot accommodate any guest to show adsorption functionality and/or accomplish the pore-shaping action), shape-memory pores require a large energy difference (the nonporous phase tends to possess a lower energy than the porous one) to facilitate the key action, i.e., complete structural transformation from the metastable state to the stable state by heating. In contrast, small energy differences, ideally zero, are beneficial for plastic pores to effectively recognize and distinguish corresponding guests (Figure 3). On the other hand, since the shape-memory process is not required, and the pore-shaping process is driven by adsorption, plastic pores can have higher energy barriers than shape-memory pores. However, flexible adsorbents with exceptionally small energy differences usually exhibit very low energy barriers, resulting in dynamic transient structural transformation (occurs transiently at nonequilibrium states, instead of at equilibrium states like other types of flexibility) including gating adsorption. As a proof of concept study, we report a crystal engineering approach for tuning the energy difference and barrier and show deeper insights for their roles toward the hypothetical plastic-pore behavior.

## Results and Discussion

### Synthesis and Structure

We have reported a pair of isostructural porous frameworks with contrasting flexibility and adsorption behaviors.^31^ [Zn_3_(OH)_2_(pzdc)(tz)] (MAF-91 or **H**, H_3_pzdc = 3,5-pyrazoledicarboxylic acid, Htz = 1,2,4-trizole) and [Zn_3_(OH)_2_(pzdc)(atz)] (MAF-92 or **A**, Hatz = 3-amino-1,2,4-trizole) both exhibit typical elastic-pore behaviors between the large-pore (*lp*) and small-pore (*sp*) states. Interestingly, the thermodynamically stable state of **H** is *lp*, whereas that of **A** is *sp*, indicating that the noncoordinating ligand side groups (−H for **H**, −NH_2_ for **A**) can effectively tune the framework energy.

**Figure 4:**
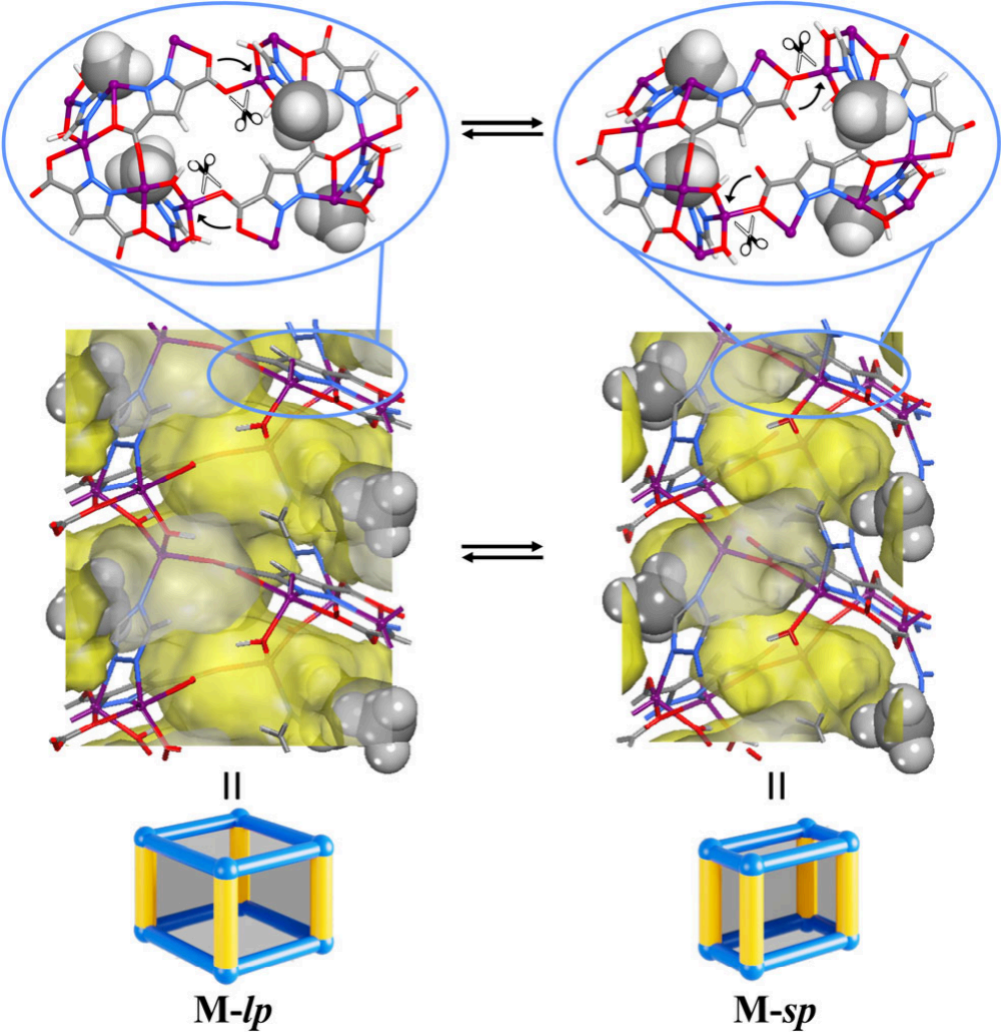
Crystal-to-crystal structural transformations between **M-*****lp*** and **M-*****sp***. Guest molecules are omitted; −CH_3_ groups are highlighted in the space-filling mode.

Similar to **H-*****lp***·DMA (DMA = *N,N*-dimethylacetamide) and **A-*****lp***·DMA, solvothermal reaction of ZnSO_4_, H_3_pzdc, and 3-methyl-1,2,4-trizole (Hmtz) in mixed solvent of DMA/H_2_O gave crystals of [Zn_3_(OH)_2_(pzdc)(mtz)]·DMA (MAF-93-*lp*·DMA or **M-*****lp***·DMA). Single-crystal X-ray diffraction (SCXRD) confirmed that **M-*****lp***·DMA is isostructural with **H-*****lp***·DMA and **A-*****lp***·DMA (Figures 4 and S2, and Table S2).^31^ Powder X-ray diffraction (PXRD) and elemental analyses indicated high purity of the as-synthesized samples (Figures S3–S5). Similar to **A** and **H**,^31^**M** can be stable in water at pH 2–12 (Figure S6).

### Single-Component Vapor/Liquid Adsorption Behaviors

**Figure 5:**
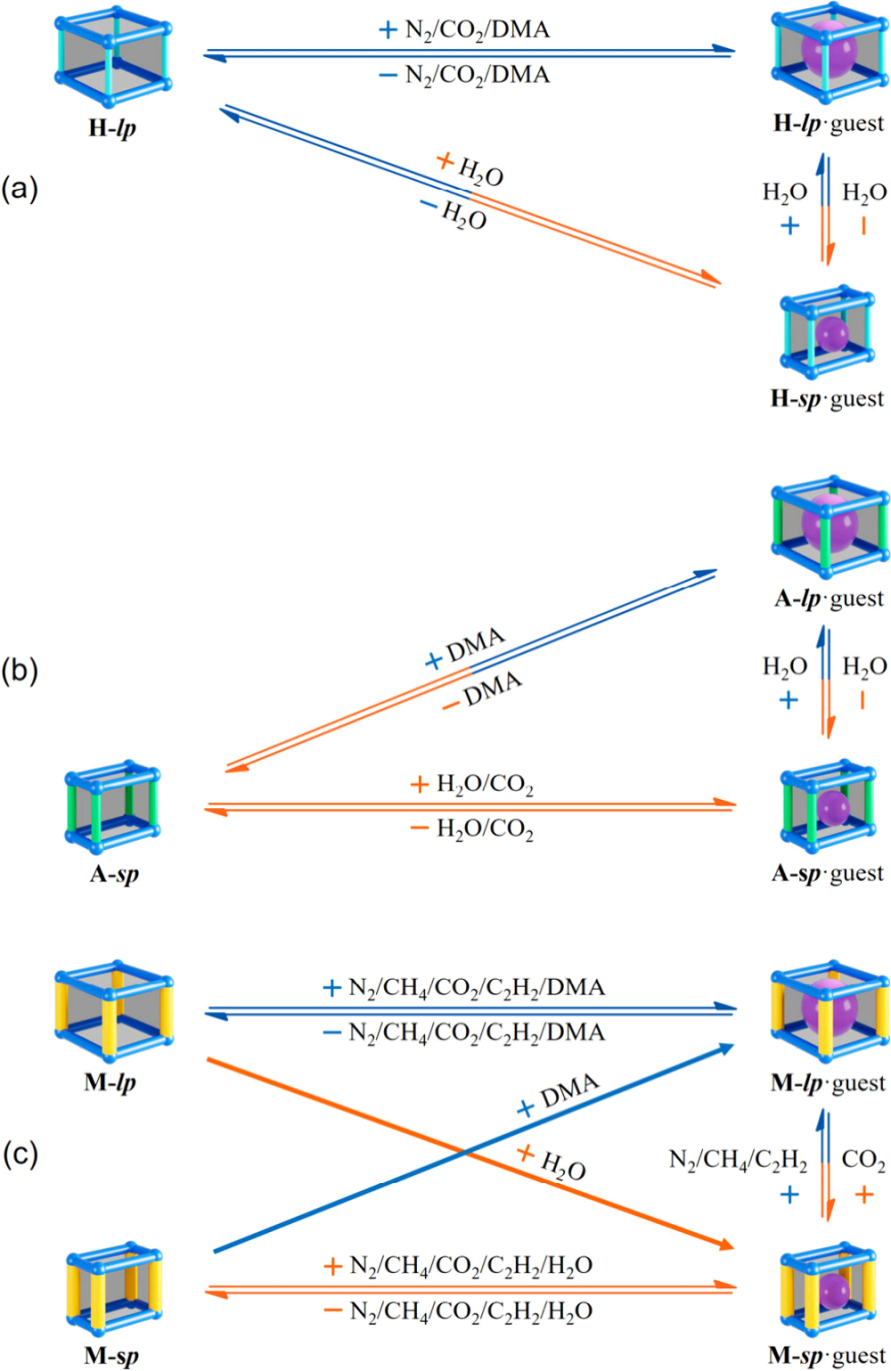
Guest responses of isostructural flexible frameworks with elastic/plastic pores. (a) Elastic behaviors of **H**, (b) elastic behaviors of **A**, and (c) plastic behaviors of **M**.

Thermogravimetry and PXRD of **H-*****lp***·DMA, **A-*****lp***·DMA, and **M-*****lp***·DMA showed that complete removal of DMA molecules gave guest-free frameworks **H-*****lp***, **A-*****sp***, and **M-*****lp***, respectively (Figures S7–S13 and Table S2). H_2_O vapor adsorption/desorption isotherms of **H-*****lp***, **A-*****sp***, and **M-*****lp*** confirmed tunable flexibility (Figures 5 and S14). They showed either one or two plateaus with uptakes of ca. 2 and 5 H_2_O molecules per formula unit (Figures S15–S16), where the host frameworks adopted the *sp* and *lp* structures, respectively (Figures S3–S5). At low pressures (*P*/*P*_0_ < 0.04), the adsorption isotherm of **A-*****sp*** showed type-I characteristics, while those of **M-*****lp*** and **H-*****lp*** showed inflection points indicative of framework contractions to **M-*****sp*** and **H-*****sp***, respectively. The framework contraction pressure for **M** is lower than that for **H**, and that of **A** can be considered as zero (because guest-free **A** is already contracted as **A-*****sp***), meaning a framework contraction trend of **A** > **M** > **H**.

In situ variable-temperature PXRD showed that, during sequential removal of H_2_O by increasing temperature, **H-*****lp***·5H_2_O transformed to **H-*****sp***·2H_2_O and then to **H-*****lp***, while **A-*****lp***·5H_2_O transformed to **A-*****sp***·2H_2_O and then to **A-*****sp*** (Figure S16), meaning that the guest-induced structural transformations are completely reversible, which is characteristic for conventional flexible adsorbents possessing elastic pores. In contrast, after complete removal of H_2_O in **M-*****sp***·2H_2_O at 100 °C, the host framework remained **M-*****sp*** rather than expanding back to **M-*****lp*** (Figure S16), meaning that both the *lp* and *sp* structures of **M** can be retained after guest removal, being characteristic for plastic pores. **M-*****sp*** started to expand to **M-*****lp*** at a relatively high temperature of 150 °C^19,25−27^ and cannot finish at the decomposition temperature of 360 °C (Figure S16), indicating that **M-*****lp*** is the thermodynamically stable phase and there is a high energy barrier for the spontaneous transformation. The incomplete phase transition at very high temperature may also indicate that the energy difference between the *lp* and *sp* states is very small (very small thermodynamic driving force).

Besides the as-synthesized, DMA-included *lp* states, we also succeeded to determine the crystal structures of all guest-free and H_2_O-included states, including **H-*****sp***·2H_2_O, **A-*****sp***·2H_2_O, **H-*****lp***·5H_2_O, **A-*****lp***·5H_2_O, and **M-*****sp***·2H_2_O, by using either SCXRD or PXRD (Figures S17–S19 and Tables S1). Similar to **A** and **H**,^31^ the *sp*/*lp* transformations of **M** also involve coordination bond reconstruction (Figure 4). By comparing the *sp* and *lp* structures of the three isostructural materials (Figure S20), it is evident that −H groups are too small to interact with other framework components, and the host backbone (omitting the role of ligand side groups) is thermodynamically stable in the *lp* state. In contrast, −NH_2_ groups provide strong intraframework attractive hydrogen bonding in the *sp* state, which lowers the energy of the *sp* state to inverse of the relative stability of *sp* and *lp* states. With low polarity, −CH_3_ groups can form very weak hydrogen bonds, which lower the energy of the *sp* state but are not enough to inverse the thermodynamic trend. Computational simulations gave *sp*–*lp* energy differences of 25.5, −19.8, and 2.8 kJ mol^–1^ for **H**, **A**, and **M**, respectively (Table S3), which was consistent with the experimental trends and structural analyses.

Since the *sp*/*lp* transformations of the isostructural **H**/**A**/**M** system involve coordination bond reconstruction, they should possess relatively high energy barriers, as compared with common examples. However, **H** and **A** do not exhibit plastic-pore or shape-memory pore behaviors, indicating that their energy barriers are still not sufficiently high. Compared with −H and −NH_2_, the larger −CH_3_ can impose greater steric hindrance to increase the energy barrier. In addition, when the relative energy of a metastable state (such as the *sp* state of **H**/**A**/**M**) is decreased, either by loading more and more guest^31^ or by isostructural framework tailoring (e.g., −H groups are replaced by −CH_3_ groups or −NH_2_ groups to increase intraframework attractive hydrogen bonds), the energies of adjacent structural conformations located at the transformation pathway should decrease simultaneously but with smaller magnitudes. Meanwhile, the energy barriers for the two opposite transformation directions increase and decrease, respectively, and become equal when the relative energy is zero, at which the spontaneous transformation (energy decrease) has the highest energy barrier (Figure S21). Considering that the *sp*–*lp* energy differences of **H** and **A** (25.5 and −19.8 kJ mol^–1^) are small enough for guest-induced structural transformations, that of **M** (2.8 kJ mol^–1^) can be regarded as negligibly small, which not only can minimize the thermodynamic driving force for the phase transition but also could be beneficial to attain a high energy barrier.

### Single-Component Gas Adsorption Behaviors

Low-temperature single-component N_2_/CO_2_ sorption isotherms with in situ PXRD showed that (Figures S12 and S22–S26) **M** adopts the *lp* state in N_2_ and a mixed *lp*/*sp* state in CO_2_ at sufficiently high pressures, respectively, regardless of the initial state (*lp* or *sp*) of the sample, which aligns with the thermodynamic principle (*lp* has a larger pore volume).^32^ After N_2_ desorption, the *lp* state was retained. In contrast, **M-*****lp*** transformed to **M-*****sp*** after CO_2_ desorption. These results demonstrated that CO_2_ and N_2_ can shape the pore to the *lp* and *sp* states, respectively. Furthermore, the **M-*****lp*** sample showed breathing behavior during N_2_/CO_2_ pressure increase, i.e., **M-*****lp*** first partially/fully transforms to **M-*****sp*** and finally fully/partially transforms to **M-*****lp*** (Figures S23–S24). The incomplete pore expanding in CO_2_ and pore shrinking in N_2_ can be attributed to the nonuniform crystal size.^19^ The breathing behavior requires the ***sp*** state to have a significantly stronger guest affinity than the ***lp*** state or a very small energy difference between the *sp* and *lp* states.^32^**M**-***sp*** shows only a slightly stronger N_2_ affinity than **M**-***lp***, which further indicates the extremely small energy difference between the two phases. For CO_2_, the guest affinity difference is much larger so that **M-*****lp*** transforms to **M-*****sp*** at a lower pressure (Figure S22).

Crystal size can influence the energy profile of the framework.^19^ We found that through repeated solvent exchange between DMA and H_2_O, the crystal size of **M** can be significantly reduced (Figures S27–S29). While literature examples typically demonstrated increased energy barriers,^21^ small-sized **M** showed a rare case of inversed thermodynamic stability (Figures S30–S31),^33−35^ further indicating the extremely small energy difference of the two phases in the as-synthesized sample. Moreover, small-sized **M** loses the plastic-pore characteristics, showing only the common elastic-pore and/or rigid-pore behaviors (Figures S32–S38), which highlight the critical roles of energy difference and energy barrier. The solvent exchange processes not only cause mechanical fatigue/alterations to crystallite size but also introduced defects (Figures S32–S34), which can also modify the energy profile.^36−38^

**Figure 6:**
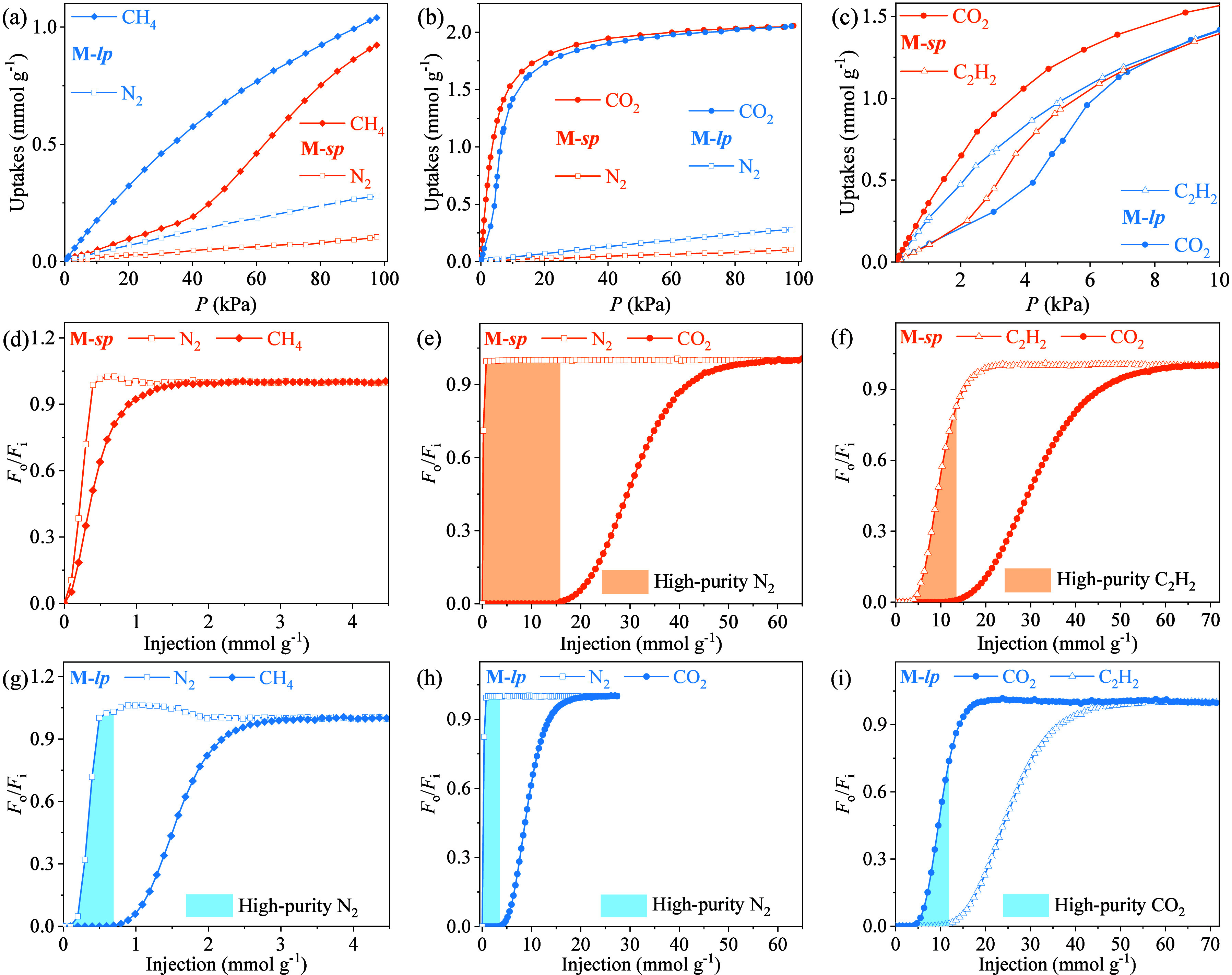
Gas adsorption and separation behaviors of the plastic-pore adsorbent. (a–c) Comparison of the single-component adsorption isotherms of **M-*****lp*** and **M-*****sp***. (a) CH_4_/N_2_, (b) CO_2_/N_2_, and (c) CO_2_/C_2_H_2_. (d–i) Comparison of the mixture breakthrough curves of **M-*****lp*** and **M-*****sp*** using different inputs. (d,g) 20:80 CH_4_/N_2_, (e,h) 1:99 CO_2_/N_2_, and (f,i) 1:1:98 CO_2_/C_2_H_2_/Ar. *F*_i_ and *F*_o_ are the flow rates of each gas at the inlet and outlet, respectively.

To investigate the adsorption/flexibility behaviors for practical applications, single-component N_2_, CO_2_, and CH_4_ sorption isotherms for **M-*****lp*** and **M-*****sp*** were measured at 298 K (Figures 6a-b, S13, and S39–S49). According to the isotherm shapes and in situ PXRD, **M-*****sp*** kept unchanged in CO_2_ and **M-*****lp*** kept unchanged in N_2_ or CH_4_, up to 8 bar. **M-*****sp*** partially transformed to **M-*****lp*** in N_2_ above 4 bar or in CH_4_ above 40 kPa, and a complete transformation was observed in CH_4_ above 2 bar. **M-*****lp*** partially and completely transformed to **M-*****sp*** in CO_2_ above 5 kPa and 4 bar, respectively. For comparison, **A-*****sp*** and **H-*****lp*** showed rigid behaviors for these gases (Figures S11 and S50),^31^ which highlights the negligibly small energy difference between **M-*****sp*** and **M-*****lp***. According to the thermodynamic principle,^32^ structural transformation of the host is driven by the increased host–guest interactions, including the increase of guest uptake and the change of guest binding affinity. The diverse structural transformation phenomena for N_2_, CO_2_, and CH_4_ adsorption indicate interestingly different adsorption affinities and effective pore sizes for these host–guest combinations.

Computational simulations showed that each pore cavity of **M-*****sp*** can accommodate 1 N_2_, 1 CO_2_, or 1 CH_4_ molecule, while that of **M-*****lp*** can accommodate 3 N_2_, 2 CO_2_, or 2 CH_4_ molecules (Figure S51). The N_2_, CO_2_, and CH_4_ adsorption enthalpies change from −28.1, −47.1, and −37.8 kJ mol^–1^ for **M-*****sp*** to −26.9, −35.9, and −33.2 kJ mol^–1^ for **M-*****lp***, respectively, in which only the CO_2_ adsorption weakens significantly from **M-*****sp*** to **M-*****lp***. According to the simulated host–guest structures, the shape and size of CO_2_ are very similar to those of the pore cavity of **M-*****sp***, whereas N_2_ and CH_4_ are obviously shorter than the pore cavity (Figure S52). Therefore, in CO_2_, the framework expansion to **M-*****lp*** is difficult and contraction to **M-*****sp*** is easy (Figures S24, S26, S44, and S48), while in CH_4_ or N_2_, the trend is opposite (Figures S23, S25, S41, S47, and S49).

### Gas Mixture Adsorption/Separation Behaviors

After CO_2_/CH_4_-induced structural transformations at 298 K and high pressures, the resultant *sp*/*lp* states can be retained after desorption. In other words, under practical conditions for adsorptive separation, the plastic pore can be shaped by the target gases. More importantly, for the target gases, the shaped pores exhibit improved adsorption affinities (Figures 1d and S1), which mimic the application logic of conventional plastics. For example, from the CH_4_-shaped to the CO_2_-shaped state, the level of CO_2_ uptake at 1 kPa (a typical pressure/concentration for CO_2_ capture in confined space) increases from 0.11 to 0.36 mmol g^–1^. Conversely, from the CO_2_-shaped state to the CH_4_-shaped state, the CH_4_ uptake at 20 kPa (a typical pressure/concentration for upgrading coalmine CH_4_) increases from 0.10 to 0.32 mmol g^–1^.

The functional switchability of plastic pores was then demonstrated by CH_4_/N_2_ and CO_2_/N_2_ mixture separation experiments at ambient temperature and pressure. Quantitative breakthrough curves using 20:80 CH_4_/N_2_ and 1:99 CO_2_/N_2_ mixture inputs were measured for **M** (Figures 6d,e,g,h and S53–S66, and Tables S4–S5), for which a single adsorbent sample was used, and **M-*****sp*** and **M-*****lp*** were obtained by in situ pore-shaping operations using high-pressure CO_2_ and CH_4_, respectively. Both states of **M** exhibit CH_4_/N_2_ and CO_2_/N_2_ selectivity because N_2_ has an obviously lower boiling point, yet their breakthrough curves differ significantly. For CH_4_/N_2_ separation, **M-*****lp*** outperforms **M-*****sp***. **M-*****lp*** showed CH_4_/N_2_ uptake and selectivity of 0.28/0.07 mmol g^–1^ and 16, respectively. Meanwhile, 0.43/0.27 mmol g^–1^ N_2_ and 0.18/0.15 mmol g^–1^ CH_4_ with 99.9%/99.99% purity can be collected in a single adsorption and desorption process, respectively. In contrast, **M-*****sp*** showed a CH_4_/N_2_ selectivity of just 8, and the productivities of high-purity N_2_/CH_4_ were lower than 0.02 mmol g^–1^. For CO_2_/N_2_ separation, the CO_2_/N_2_ selectivity of **M-*****sp*** was 7 times that of **M-*****lp***, and the high-purity CO_2_/N_2_ productivities were approximately 3–4 times that of **M-*****lp***.

Some adsorbents may show high performances for several applications (e.g., CO_2_ capture and CH_4_ upgrading),^39−43^ but only plastic pores can show inverse adsorption selectivity to meet different practical requirements (Figures 1d and S1). The *sp* and *lp* states of **M** can be obtained by CO_2_ and C_2_H_2_ shaping, respectively (Figures 6c and S44–S45). Quantitative breakthrough experiments using a CO_2_/C_2_H_2_/Ar mixture showed that the CO_2_-shaped and C_2_H_2_-shaped states exhibit CO_2_/C_2_H_2_ and C_2_H_2_/CO_2_ selectivities of 3.2 and 2.6 under the same conditions (Figures 6f,i, S67–72, and Table S6), which are beneficial for purification of C_2_H_2_ and CO_2_, respectively.^44^ The inversed selectivities arise from the different guest recognition abilities of **M-*****sp*** (47.1/38.2 kJ mol^–1^) and **M-*****lp*** (35.9/49.6 kJ mol^–1^) toward CO_2_/C_2_H_2_, which can be visualized from the computational simulated host–guest structures (Figures S51–S52). Although there have been some previous examples for inversion of adsorption selectivity, they require the use of more than one material (obtained by either direct syntheses, postsynthetic modifications, or other irreversible treatments)^45−52^ and/or the change of conditions (temperature, pressure, and/or guest compositions).^31,53,54^

## Conclusion

We demonstrated that pore structures can exhibit not only rigid and elastic but also plastic behaviors. Being more advantageous than rigid and elastic pores, in principle, plastic pores allow not only on-demand switching of adsorption/separation functions of a given porous material but also spontaneous optimization of the adsorption performances. The structural optimization process is reminiscent of the template effect during adsorbent syntheses. However, it is difficult to precisely control the pore size/shape by templates, and gas templates remain an unexplored strategy, due to the competition of solvent molecules and other molecular/ionic components.

As a straightforward requirement for plastic pores, suitably high energy barriers can be rationally achieved by crystal engineering strategies. For a particular framework prototype, the energy barrier may not be an independent parameter but is connected to others such as the energy difference. Since the ideal pore-shaping conditions (e.g., high temperature/pressure) should be far away from those of adsorption/separation operations and the pore-shaping processes (guest adsorption) automatically decrease the energy barrier, one may not need to be concerned about the energy barriers being excessively high. On the other hand, increasing the number (much larger than two) of metastable states with near-zero energy difference is also a key target/challenge toward ideal plastic pores.

## Supporting Information Available

Experimental details, additional figures/tables (PDF)
**M-*lp***·DMA, **M-*sp***·2H_2_O, **M-*lp***, **M-*sp***, and **A-*sp***·2H_2_O (CIF)

## Supplementary Material

oc4c02155_si_001.pdf

oc4c02155_si_002.cif
