# Supplementary material for: Plastic Pores for Switchable and Optimized Adsorption Behaviors
Source: ACS Cent Sci. 2025 Mar 13;11(3):479–85. doi: 10.1021/acscentsci.4c02155 (PMC11950851; doi:10.1021/acscentsci.4c02155)
Supplement: Supplementary file 1 — oc4c02155_si_001.pdf [file oc4c02155_si_001.pdf]

**Supporting Information for**

# Plastic Pores for Switchable and Optimized Adsorption Behaviors

Xue-Wen Zhang, Rong-Hua Wang, Jie-Peng Zhang*, and Xiao-Ming Chen

*MOE Key Laboratory of Bioinorganic and Synthetic Chemistry, School of Chemistry and Chemical Engineering, IGCME, Sun Yat-Sen University, Guangzhou 510275, China*

*E-mail: zhangjp7@mail.sysu.edu.cn

# Supplementary Index


## Methods (pages S1-S2)

**Fig. S1 (page S3).** Comprehensive adsorption and structural transformation behaviors of rigid/flexible pores.

**Fig. S2 (page S4).** The framework and pore structures at *lp* and *sp* states.

**Figs S3-S6 (pages S5-S8).** Conventional PXRD patterns of **H**, **A**, and **M**.

**Fig. S7 (page S9).** Thermogravimetry curves of the as-synthesized samples.

**Figs S8-S10 (pages S10-S12).** Variable-temperature PXRD patterns of the as-synthesized samples.

**Figs S11-S13 (pages S13-S15).** Detailed illustration of all adsorption/flexibility behaviors.

**Fig. S14 (page S16).** $H_2O$ adsorption/desorption isotherms.

**Fig. S15 (page S17).** Thermogravimetry curves of the $H_2O$-adsorbed samples.

**Fig. S16 (page S18).** Variable-temperature PXRD patterns of the $H_2O$-adsorbed samples.

**Figs S17-S19 (pages S19-S21).** Pawley and Rietveld refinements for the $H_2O$-adsorbed samples.

**Fig. S20 (page S22).** Comparison of the intraframework hydrogen-bonding distances.

**Fig. S21 (page S23).** Typical evolution trends of the energy profile of a bistable system.

**Figs S22-S26 (pages S24-S28).** *In-situ* sorption–PXRD measurements of **M** at 77/195 K.

**Figs S27-S38 (pages S29-S39).** Purity, stability, and porosity/adsorption characterizations for the small-sized **M.**

**Figs S39-S46 (pages S40-S47).** *In-situ* sorption–PXRD measurements of **M** at 298 K**.**

**Fig. S47 (page S48).** PXRD patterns of **M-*lp*** and **M-*sp*** in high-pressure $N_2$ at 298 K.

**Fig. S48 (page S49).** PXRD patterns of **M-*lp*** and **M-*sp*** in high-pressure $CO_2$ at 298 K.

**Fig. S49 (page S50).** PXRD patterns of **M-*lp*** and **M-*sp*** in high-pressure $CH_4$ at 298 K.

**Fig. S50 (page S51).** $N_2/CO_2/CH_4$ adsorption/desorption isotherms of **H-*lp*** and **A-*sp***.

**Fig. S51 (page S52).** Simulated structures of $CO_2/N_2/CH_4/C_2H_2$-saturated **M-*sp*** and **M-*lp***.

**Fig. S52 (page S53).** Detailed host-guest interactions of $CO_2/CH_4/N_2/C_2H_2$-loaded **M-*sp*** and **M-*lp***.

**Fig. S53 (page S54).** The column breakthrough experiment design for *in-situ* pore-shaping.

**Fig. S54 (page S55).** Breakthrough curves for determination of dead-space volume.

**Figs S55-S72 (pages S56-S73).** Detailed breakthrough curves for mixture separation.

**Table S1 (page S74).** Key parameters and behaviors of different types of flexibility.

**Table S2 (page S75).** Crystallographic data and structural refinement details.

**Table S3 (page S76).** Comparison of framework energies.

**Tables S4-S6 (pages S76-S77).** Comparison of the separation performances of **M-*sp*** and **M-*lp***.

Methods

**General methods.** All reagents and solvents were commercially purchased and used without further purification. High-purity He (99.999%), Ar (99.999%), $CH_4$ (99.999%), $CO_2$ (99.999%), $N_2$ (99.999%), and/or $C_2H_2$ (99.9%) were used directly or after mixing in the pipeline for isotherm and column breakthrough experiments. Elemental analyses (C, H, N) were performed on a Vario EL Elemental Analyzer. Thermogravimetry analyses were carried out on a TA-Q50 system under $N_2$ flow with a heating rate of 10 $^{o}$C min$^{-1}$. Conventional power X-ray diffraction (PXRD) data were collected using a Bruker D8 advance X-ray powder diffractometer (Cu-K*α*) at room temperature with a scanning speed of 0.02$^{o}$/step and 10$^{o}$/min. *In-situ* variable-temperature (5 $^{o}$C min$^{-1}$) and variable-pressure (above 1 bar) PXRD data were collected (15 min after reaching the target temperature/pressure) using a Rigaku SmartLab X-ray powder diffractometer (Cu-Kα) with a scanning speed of 0.02$^{o}$/step and 10$^{o}$/min. PXRD-synchronized adsorption measurements were carried out using a Rigaku SmartLab with Cu-Kα radiation connected to MicrotracBEL Max adsorption equipment, in which each PXRD pattern was started to scan (0.02$^{o}$/step and 8$^{o}$/min) just after the corresponding isotherm data point was recorded.

**Syntheses.** $[Zn_3(OH)_2(pzdc)(mtz)]$·DMA (**M-*lp***·DMA): A mixture of $ZnSO_4$·$7H_2O$ (0.034 g, 0.12 mmol), $H_3pzdc$ (0.007 g, 0.04 mmol), Hmtz (0.003 g, 0.04 mmol), DMA (4 mL), and $H_2O$ (4 mL) was stirred for 15 min in air, then transferred and sealed in a 15-mL Teflon reactor. After heated at 140 °C for 72 hours, the reactor was cooled to room temperature at a rate of 5 °C h$^{-1}$. The resulting colorless crystals were filtered, washed successively by DMA and MeOH, and then dried in air (Yield *ca*. 55%). EA for $[Zn_3(OH)_2(C_5HO_4N_2)(C_3H_4N_3)]$·$C_4H_9NO$ (%): calcd: C, 26.07; H, 2.90; N, 15.20; found: C, 26.32; H, 2.82; N, 15.06. IR (4000−400 cm$^{-1}$): 3626(w), 3014(w), 2934(w), 1640(s), 1583(s), 1520(m), 1429(m), 1374(s), 1219(m), 1065(m), 895(w), 833(m), 806(w), 784(m), 452(w).
$[Zn_3(OH)_2(pzdc)(tz)]$·DMA (**H-*lp***·DMA): The same reaction method for **M-*lp***·DMA was used except that the ligand was replaced by Htz.[30] EA for $[Zn_3(OH)_2(C_5HO_4N_2)(C_2H_2N_3)]$·$C_4H_9NO$ (%): calcd: C, 24.56; H, 2.42; N, 15.63; found: C, 24.55; H, 2.48; N, 15.61.
$[Zn_3(OH)_2(pzdc)(atz)]$·DMA (**A-*lp***·DMA): The same reaction method for **M-*lp***·DMA was used except that the ligand was replaced by Hatz.[30] EA for $[Zn_3(OH)_2(C_5HO_4N_2)(C_2H_3N_4)]$·$C_4H_9NO$ (%): calcd: C, 23.85; H, 2.71; N, 17.71; found: C, 24.00; H, 2.45; N, 17.72.

**Single-crystal X-ray crystallography.** Diffraction intensities were collected on a Pilatus XtaLAB P300DS or a Rigaku Oxford SuperNova diffractometer with graphite monochromated Cu-Kα radiation. The structures were solved by the direct method and refined with the full-matrix least-squares technique using the SHELXTL program package. Anisotropic thermal parameters were applied to all non-hydrogen atoms. Hydrogen atoms were generated geometrically. Detailed structure determination parameters and crystallographic data were summarized in Table S1.

**Power X-ray diffraction crystallography.** PXRD patterns for Pawley or Rietveld refinements of **H-*sp***·$2H_2O$, **H-*lp***·$5H_2O$, and **A-*lp***·$5H_2O$ were collected on a Bruker D8 advance X-ray powder diffractometer (Cu-Kα) using the capillary transmission mode with a scanning speed of 0.02$^{o}$/step and 0.5$^{o}$/min. The Pawley and Rietveld refinements were carried out using the Reflex plus module of Materials Studio 5.5. The initial structures of **H-*sp***·$2H_2O$, **H-*lp***·$5H_2O$, and **A-*lp***·$5H_2O$ were built according to those of **M-*sp***·$2H_2O$ and **H-*lp*** and then optimized by the Dmol$^3$ module. The pseudo-Voigt profile parameters, background parameters, unit-cell parameters, zero point of the diffraction pattern, global isotropic atom displacement parameters, Berar–Baldinozzi asymmetry correction parameters, and March–Dollase preferred orientation correction parameters were optimized step by step to improve the agreements between the calculated and the experimental patterns.

**Single-component gas adsorption.** Gas adsorption/desorption isotherms were measured with automatic volumetric adsorption apparatuses (BELSORP max II or ASAP 2020M). The measurement temperature (298 K) was controlled by a water bath (298 K) or a semiconductor refrigeration system. Before the sorption experiments, the samples (100-200 mg) were treated in high vacuum for 2 h at 373/573 K.

**Mixture breakthrough experiments.** The design of column breakthrough experiments with *in-situ* pore shaping is shown in Fig. S55. The total input flow rate for all processes was 2 $cm^3$(STP) $min^{-1}$. Microcrystalline sample of guest-free **M** (0.9012 g) was packed in a stainless-steel column with a length of 10 cm and an internal diameter of 0.46 cm ($V$ = 1.66 $cm^3$). To carry out pore shaping, i.e. switch the sample structure/state, introduce a flow of $CH_4$ or $CO_2$ at 298 K and 8 bar for 2 h. Before each breakthrough experiment, the column was activated by passing Ar and heated at 373 K for 2 h, and then cooled to the measurement temperature of 298 K. The inlet pressure, being dependent on the sample state and gas composition, was monitored throughout the whole breakthrough experiment, which kept ca. 1.05 atm. The outlet gas stream passed through a six-way valve, and the collected gas (amount instead of concentration) was analyzed online by a gas chromatography (Agilent 7890A) with a TCD detector (G3440A) and a PLOT/Q or Cp-Molsieve 5 Å column. The gas retention in the breakthrough manifold was calculated by integrating the breakthrough curve based on flow rate. The equilibrium retention of non-adsorbing gas He was regarded as the dead space of the breakthrough manifold at the experimental conditions.

**Computational simulations.** All simulations were performed in the Materials Studio 5.5 package. The saturation uptakes were simulated by the GCMC method through the Fixed pressure task in the Sorption module at 100 kPa and the corresponding boiling point. The host and guest were both set rigid. The simulation box consisted of one unit-cell and the Metropolis method based on the Dreiding forcefield was used. The QEq partial charges and Mulliken charges were employed to the atoms of the framework and guest molecules, respectively. The cutoff radius was chosen as 15.5 Å for the Lennard-Jones potential, and the equilibration steps and production steps were both set as $1 \times 10^7$.

The energies of hosts, guest molecules, and host-guest systems were obtained by the periodic density functional theory (PDFT) optimization through the $Dmol^3$ module. The host and guest were both set rigid. The widely used generalized gradient approximation (GGA) with the Perdew-Burke-Ernzerhof (PBE) functional and the double numerical plus d-functions (DNP) basis set were used for the non-metal atoms. The effective core potentials (ECP) were employed for the metal atoms. The DFT including the long-range dispersion correction (DFT-D) was also taken into account using the Grimme (G06) semiempirical method to describe the long-range van der Waals interactions. For all the DFT-D calculations, the energy, gradient and displacement convergence criterions were set as $1 \times 10^{-5}$ Ha, $2 \times 10^{-3}$ Ha and $5 \times 10^{-3}$ Å, respectively. The adsorption enthalpy $\Delta H = E_{\text{host+guest}} - E_{\text{host}} - E_{\text{guest}}$, where $E_{\text{host+guest}}$ and $E_{\text{host}}$ ($E_{\text{guest}}$) are the energies of the final host-guest system and the isolated host (guest), respectively.

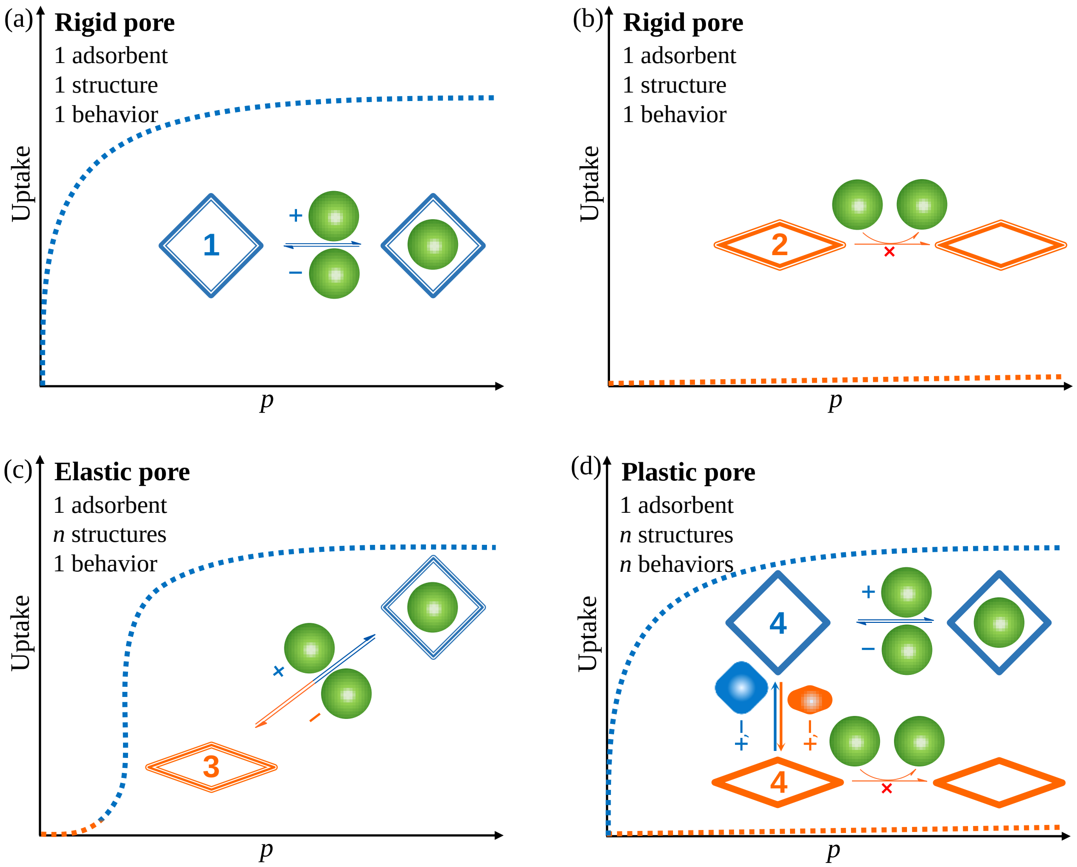


**Figure S1.** Adsorption and structural transformation behaviors of rigid/flexible pores in the large-guest cases (the adsorbate is too large for the *sp* adsorbents but small enough for the *lp* adsorbents). (a,b) Rigid pore, (c) elastic pore, and (d) plastic pore for large guests. Figure 1 show the small-guest cases.

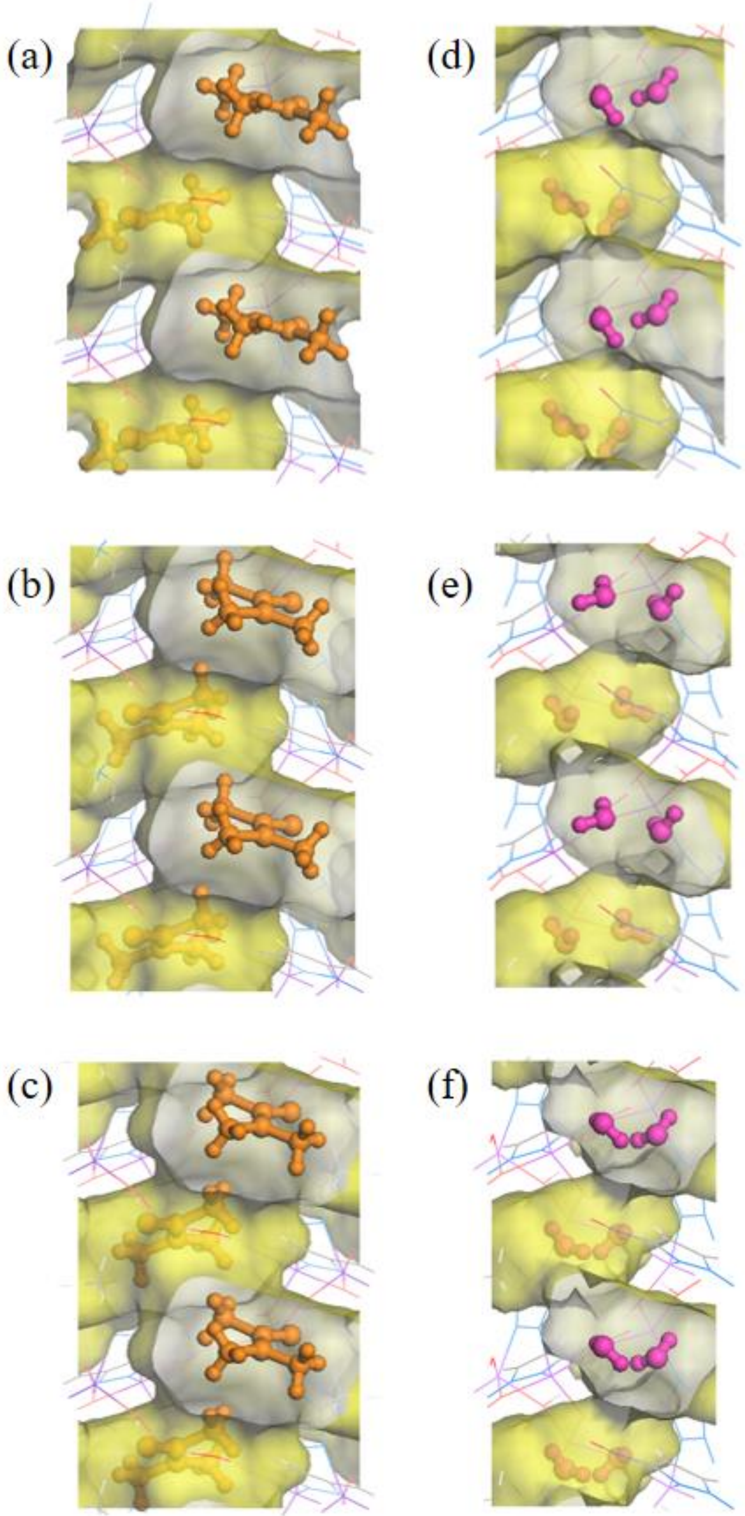


**Figure S2.** The framework (lines) and pore (surfaces) structures at *sp* and *lp* states. (a) **H-*lp*** ·DMA, (b) **A-*lp*** ·DMA, (c) **M-*lp*** ·DMA, (d) **H-*sp*** ·2$H_2O$, (e) **A-*sp*** ·2$H_2O$, and (f) **M-*sp*** ·2$H_2O$. Guest molecules are drawn in the ball-and-stick mode.

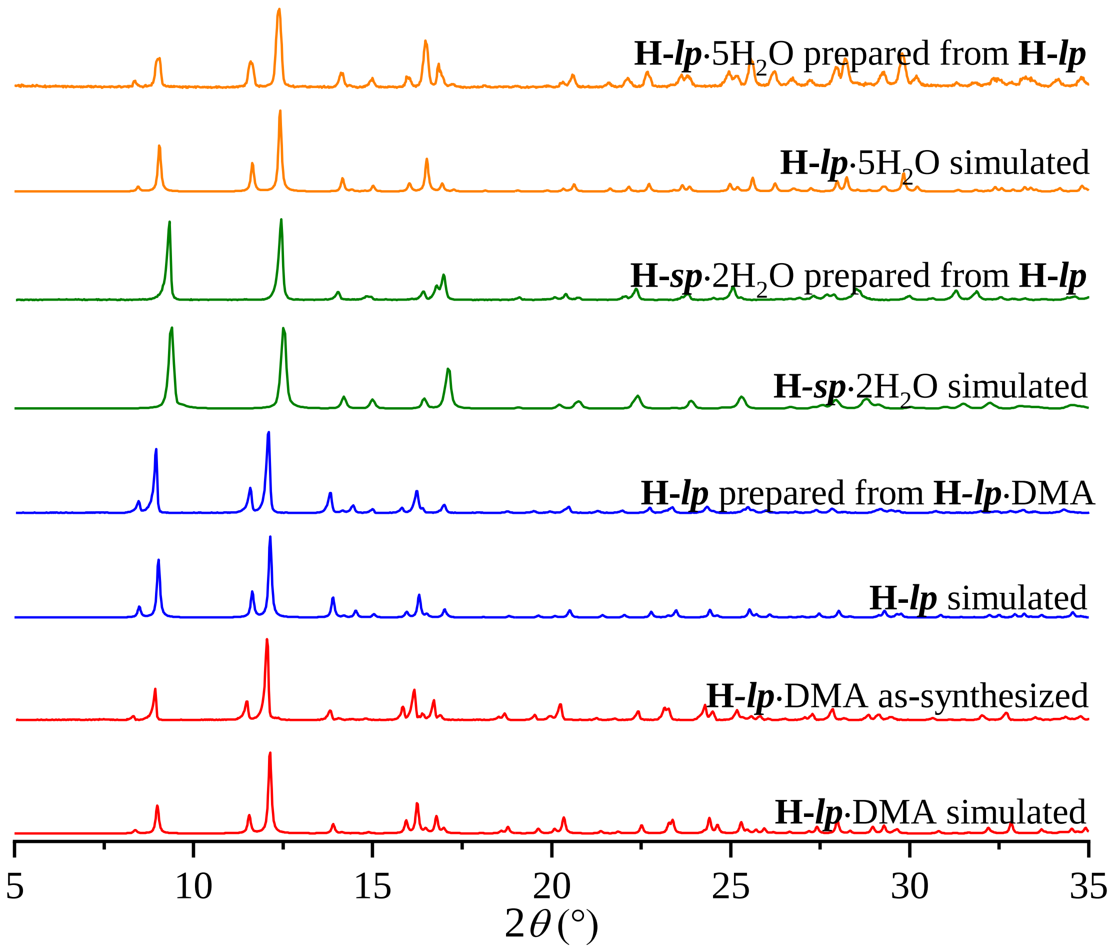


**Figure S3.** Conventional PXRD patterns of **H** (**H-*lp*** ·DMA, **H-*lp***, **H-*sp*** ·2$H_2O$, and **H-*lp*** ·5$H_2O$). In air (RH ~ 60%), **H-*lp*** first transformed to **H-*sp*** within 0.5 h, and then expand back to **H-*lp*** after ca. 1 h in water.

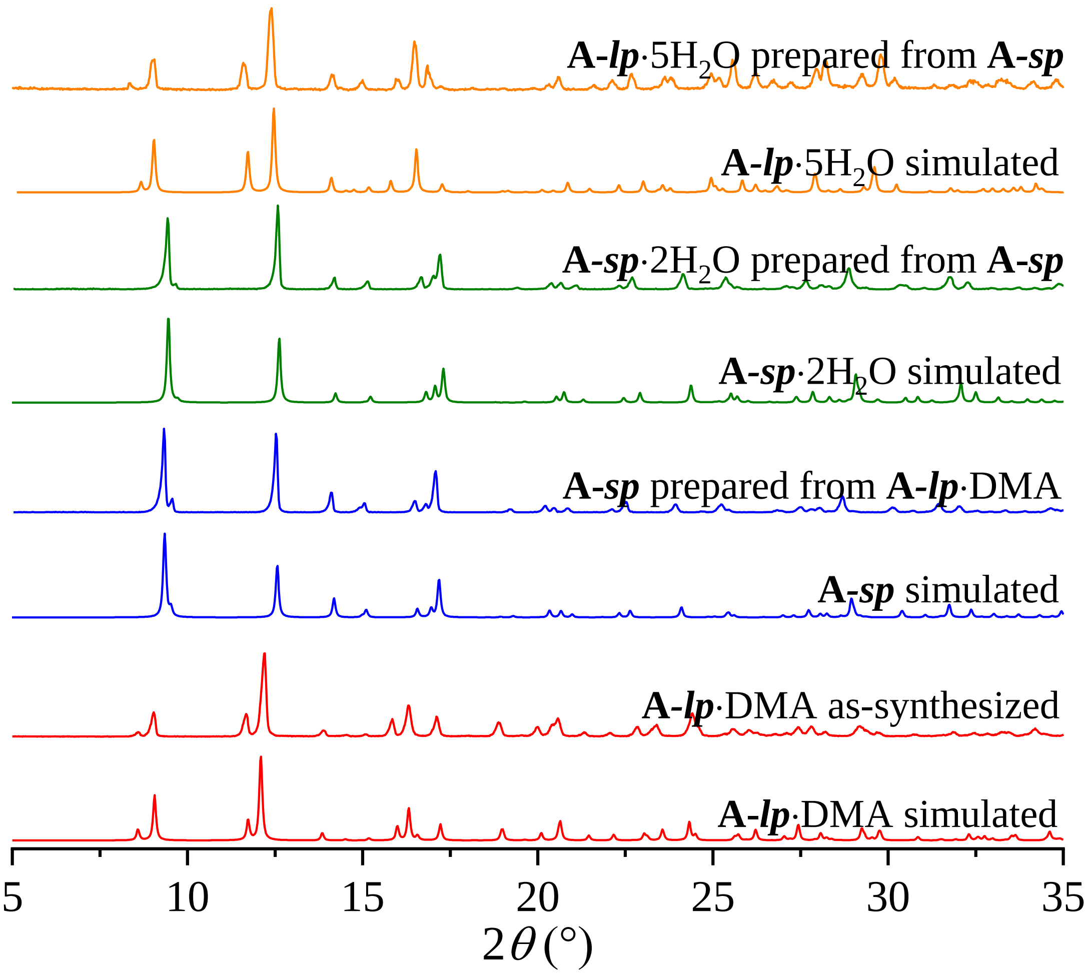


**Figure S4.** Conventional PXRD patterns of **A** (**A-*lp*** ·DMA, **A-*sp***, **A-*sp*** ·$2H_2O$, and **A-*lp*** ·$5H_2O$). In air (RH ~ 60%), **A-*sp*** can keep the host structure for at least 24 h, and it can expand back to the **A-*lp*** structure in water.

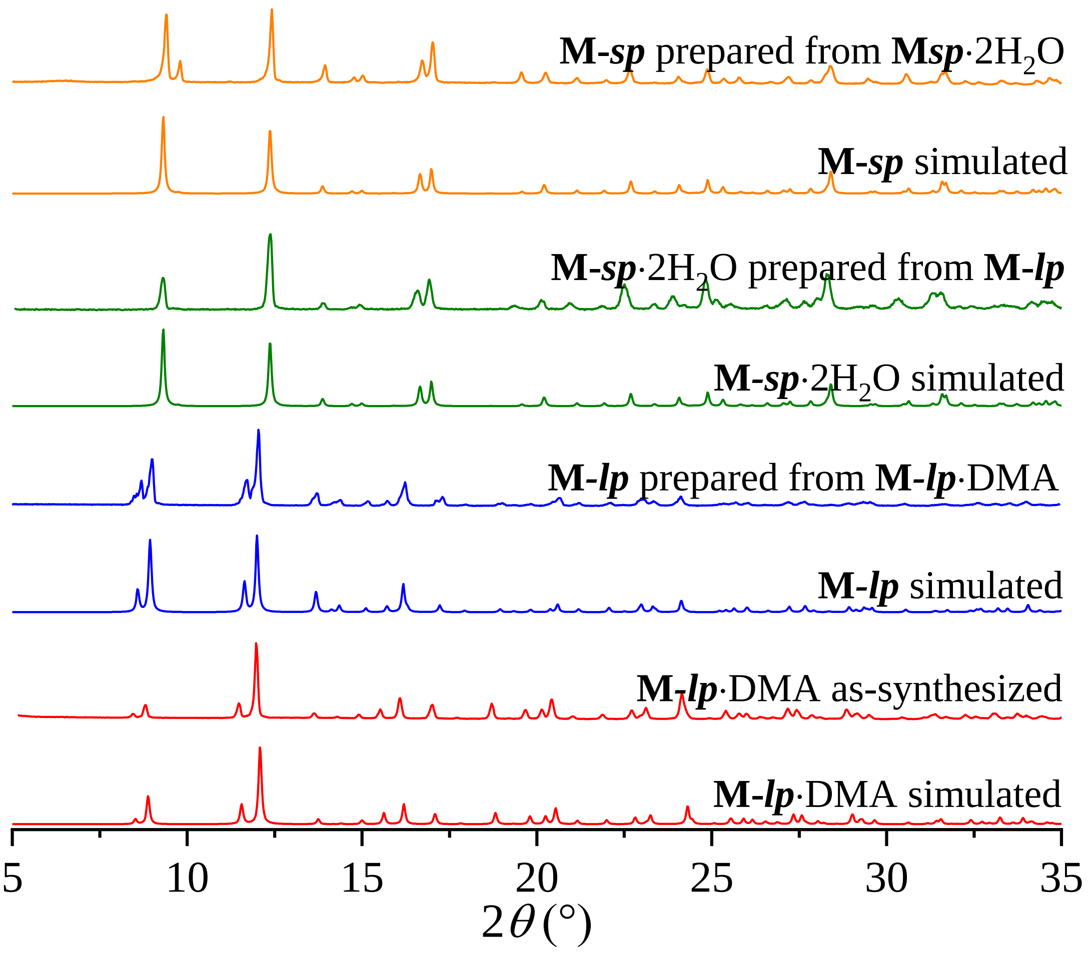


**Figure S5.** Conventional PXRD patterns of **M** (**M-*lp*** ·DMA, **M-*lp***, **M-*sp*** ·2$H_2O$, and **M-*sp***). In air (RH ~ 60%) or water, **M-*lp*** transformed to **M-*sp*** ·2$H_2O$.

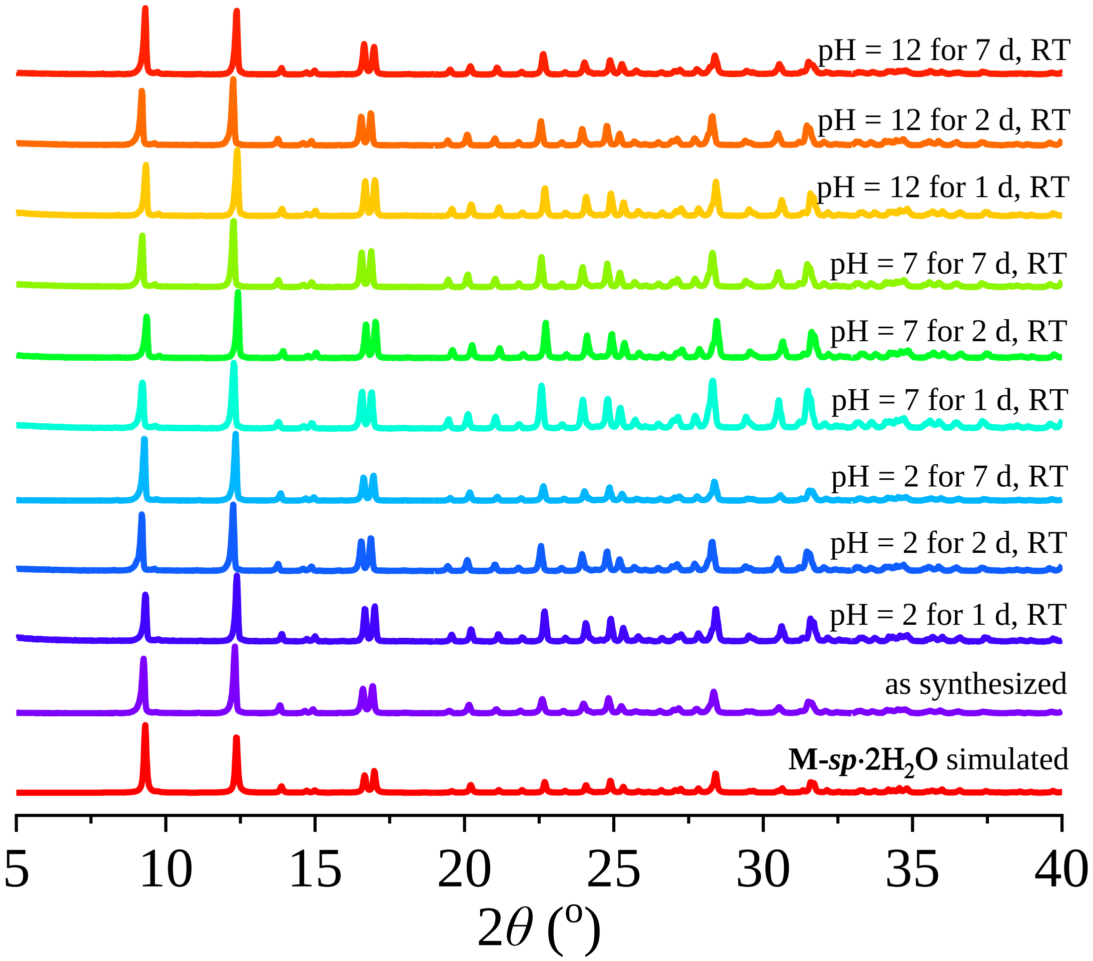


**Figure S6.** PXRD patterns of **M** (dissolves at pH 1 and pH 13) after immersed in $H_2O$ at room temperature (RT) for 7 days.

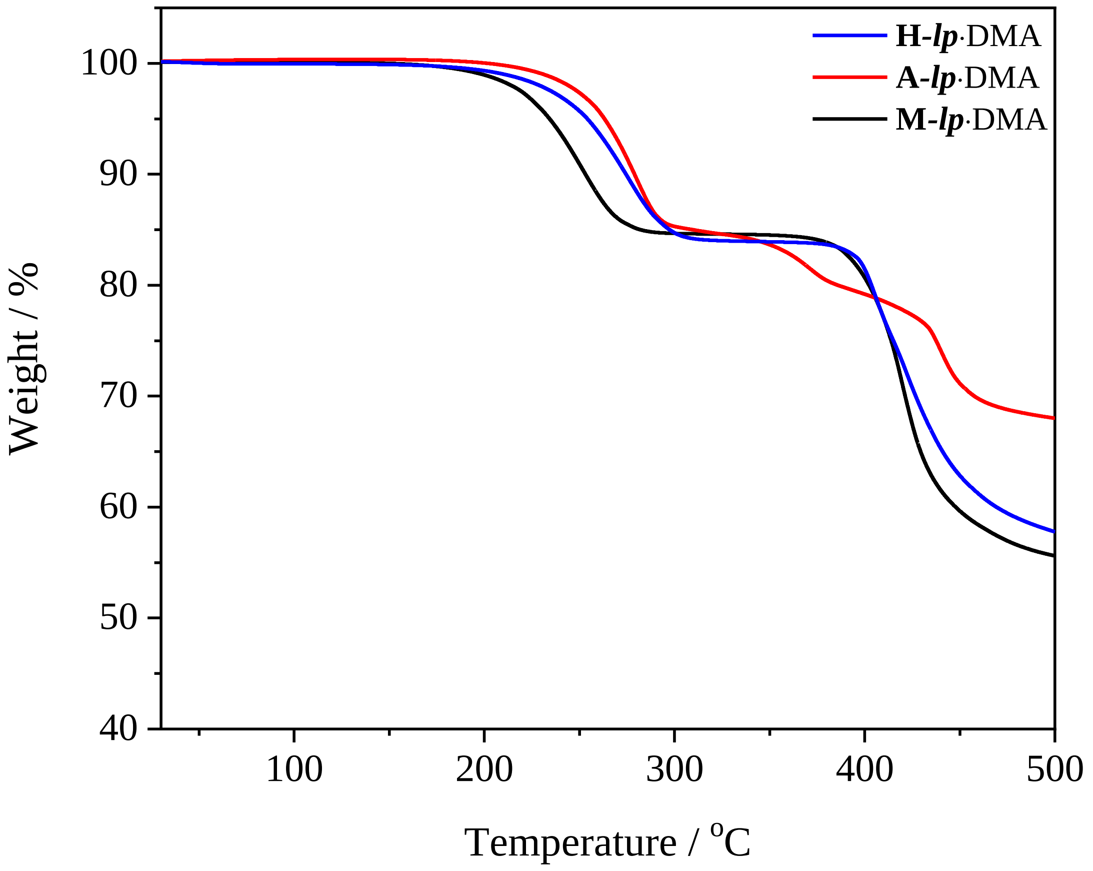


**Figure S7.** TG curves of the as-synthesized samples **H-*lp*** ĐMA, **A-*lp*** ĐMA, and **M-*lp*** ĐMA.

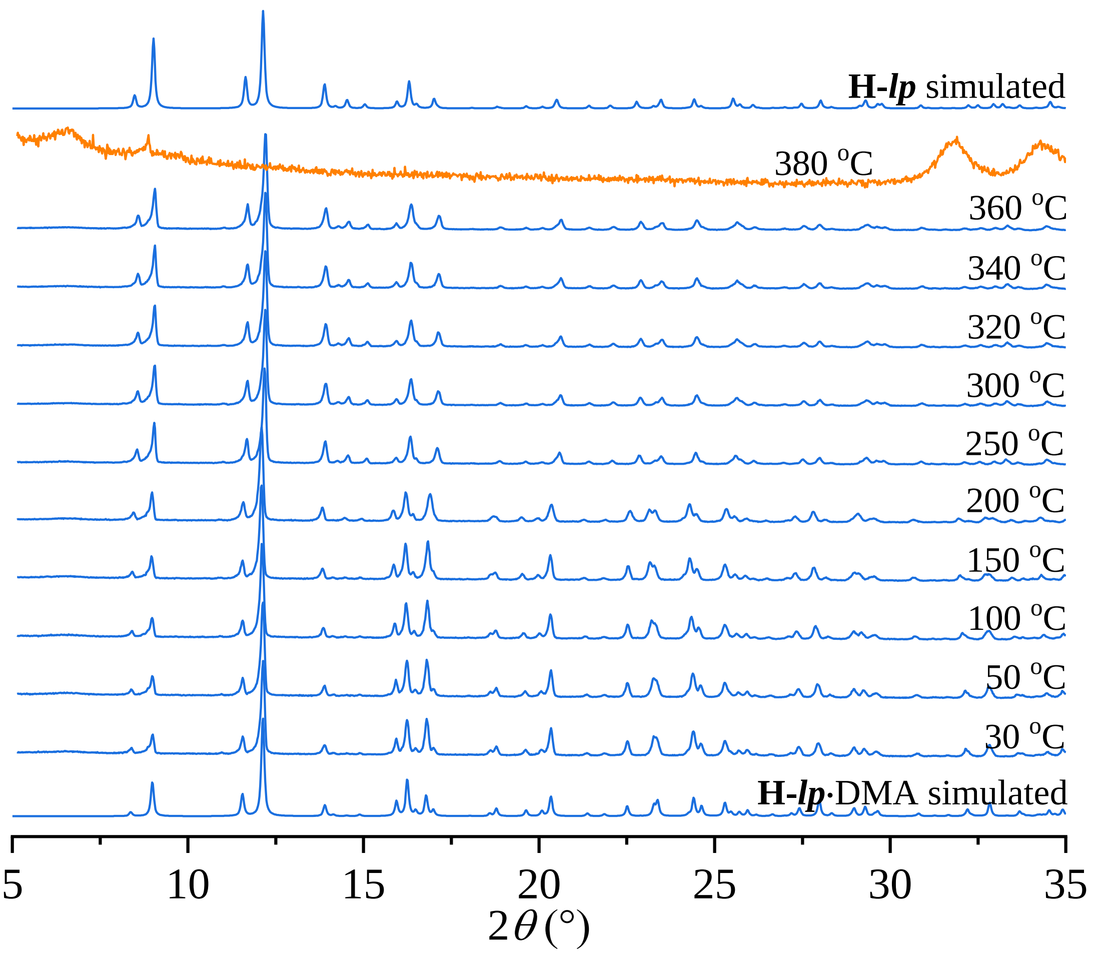


**Figure S8.** *In-situ* variable-temperature PXRD patterns of **H-*lp*** ·DMA.

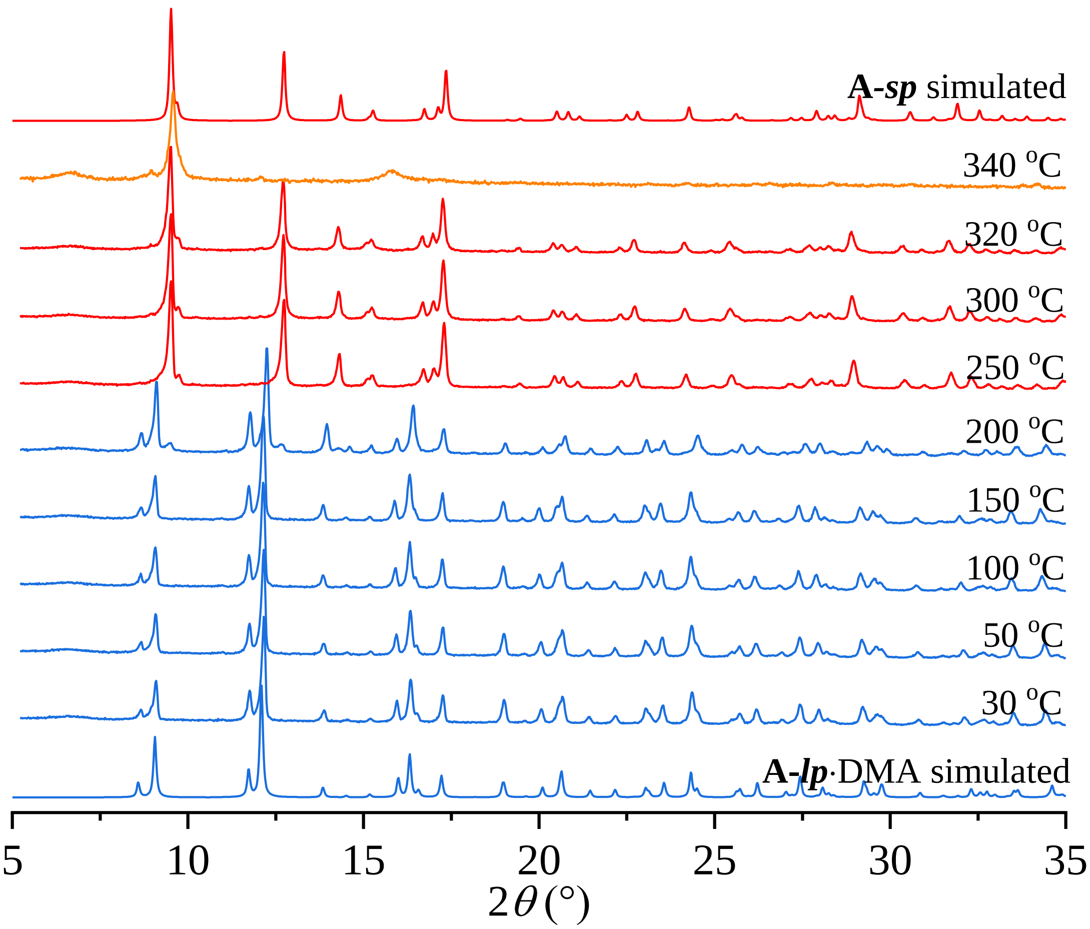


**Figure S9.** *In-situ* variable-temperature PXRD patterns of **A-*lp*** ·DMA.

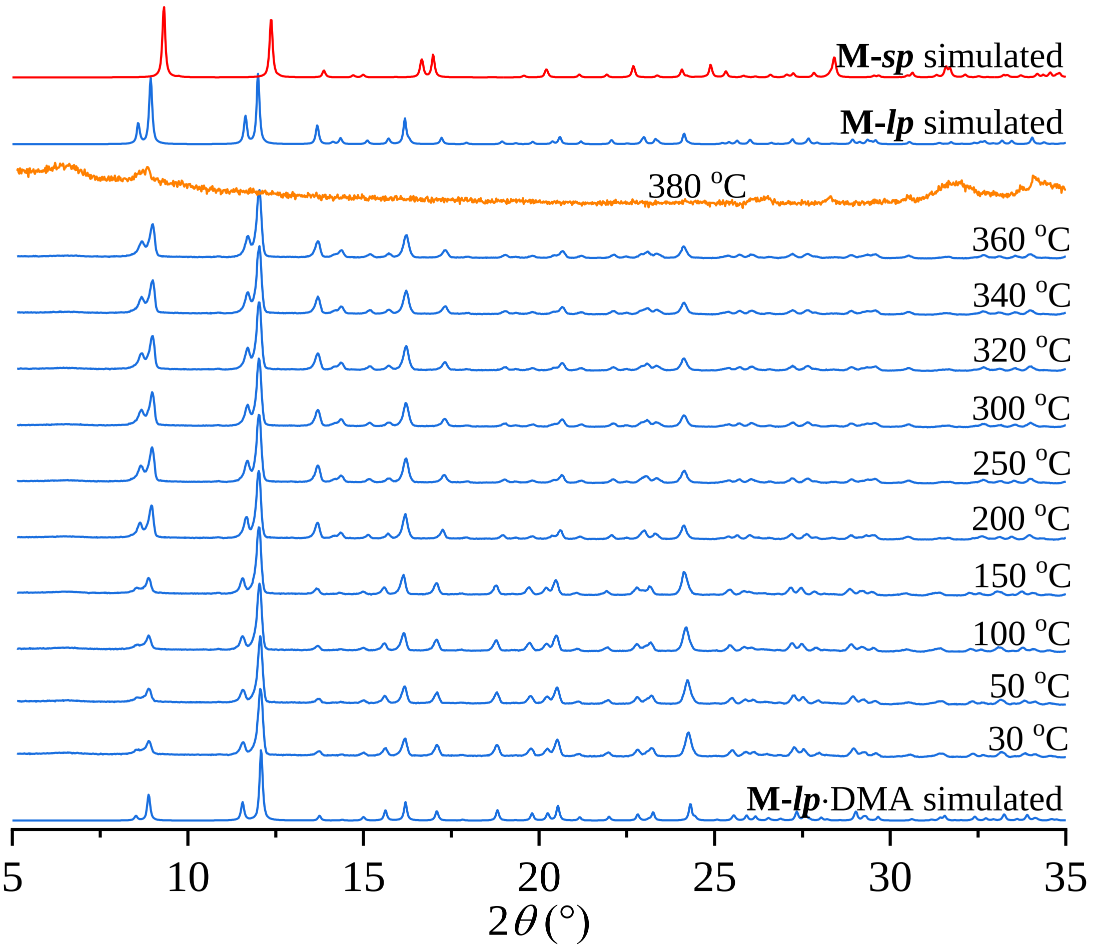


**Figure S10.** *In-situ* variable-temperature PXRD patterns of **M-*lp*** ·DMA.

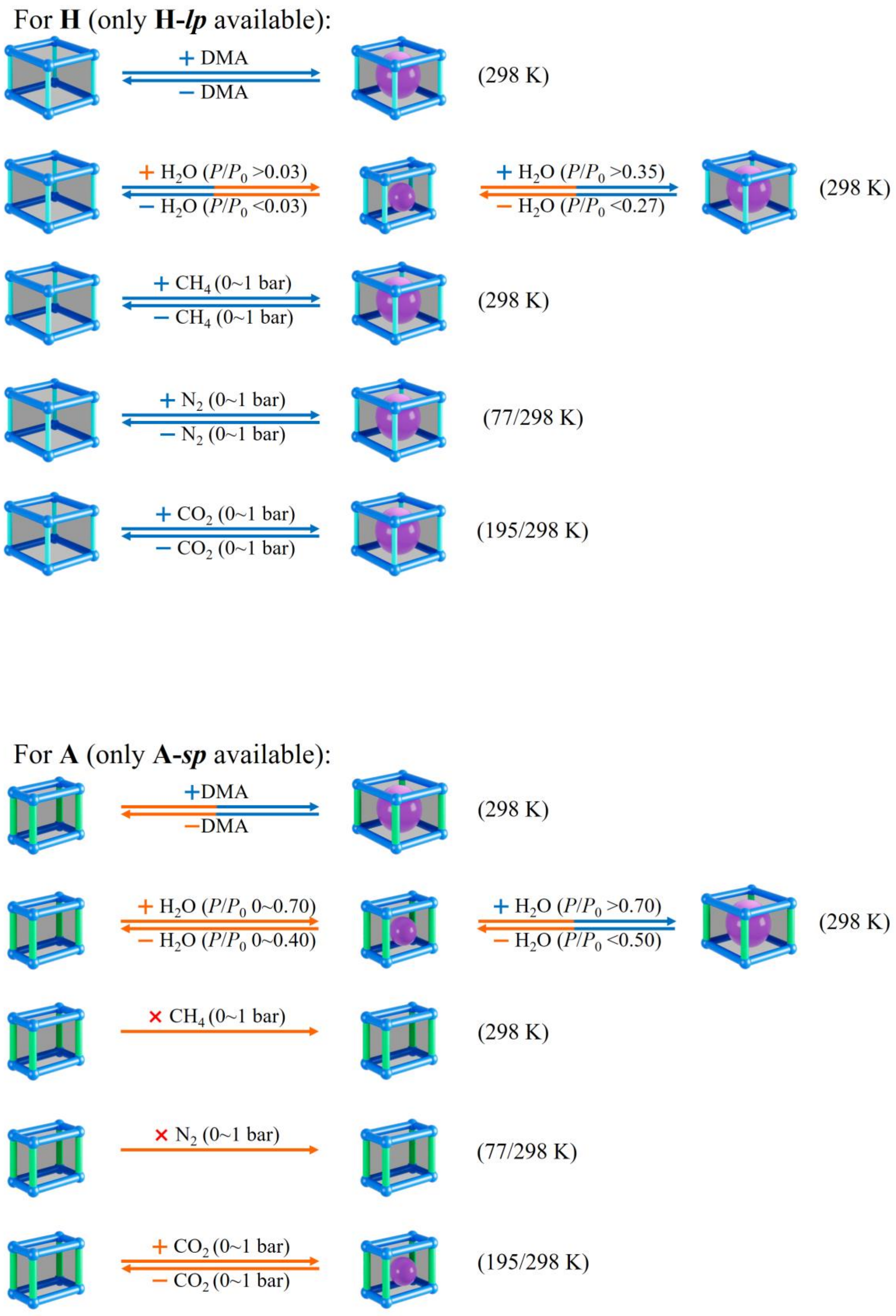


**Figure S11.** Detailed illustration of all adsorption and flexibility behaviors of **H** and **A**.

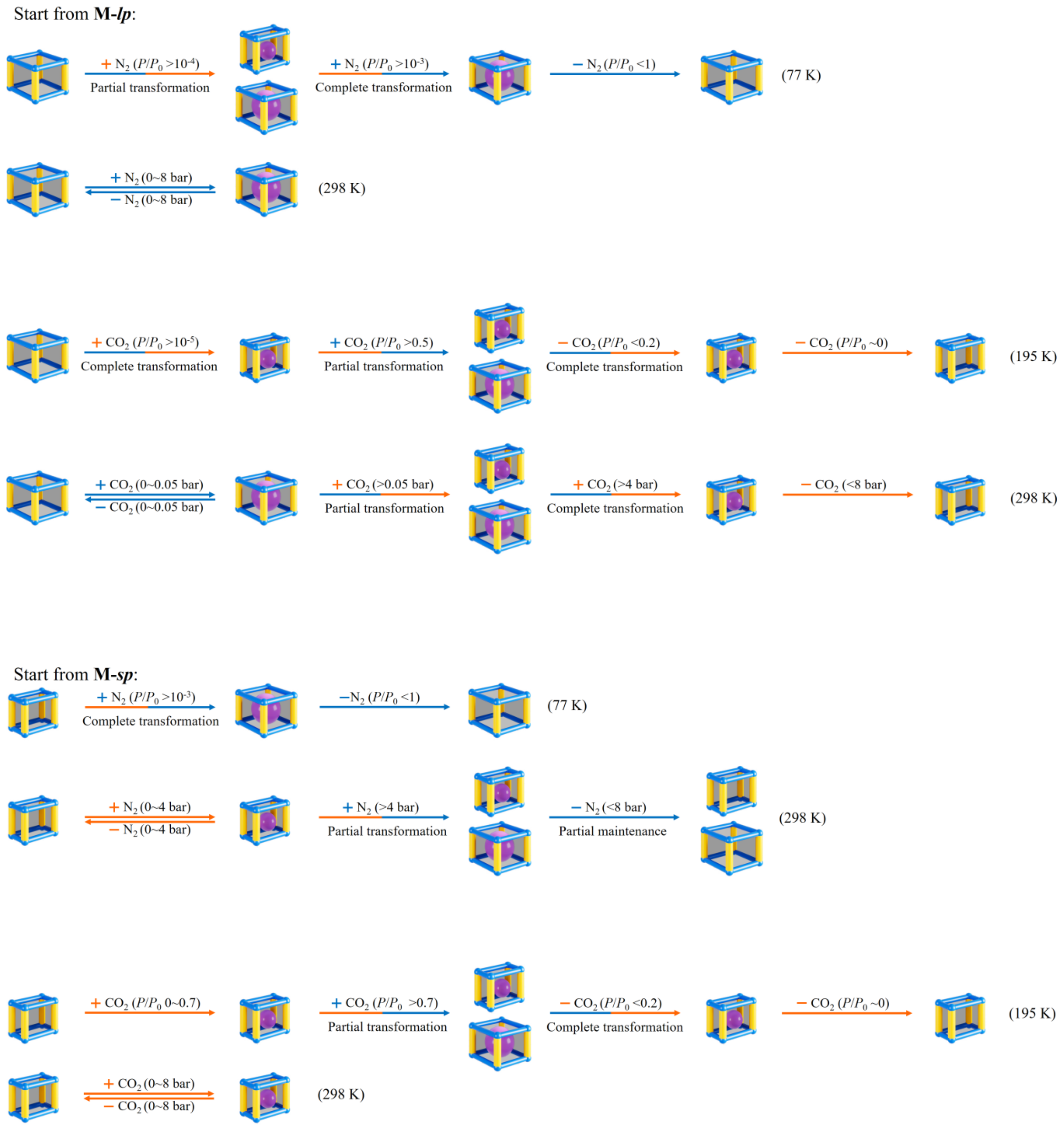


**Figure S12.** Detailed illustration of all adsorption and flexibility behaviors of **M** for $CO_2$ and $N_2$ at different temperatures.

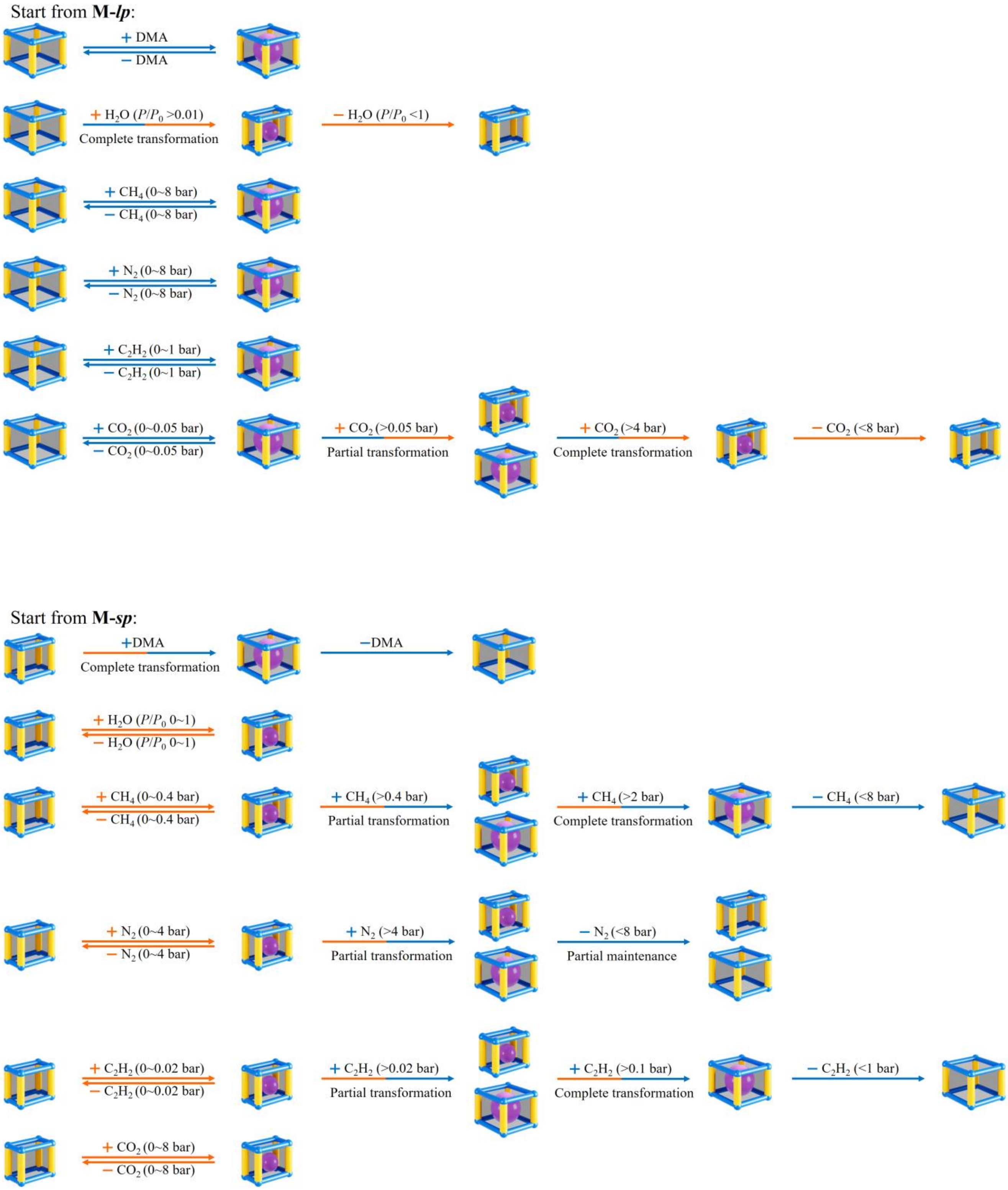


**Figure S13.** Detailed illustration of all adsorption and flexibility behaviors of **M** for various guest molecules at room temperature.

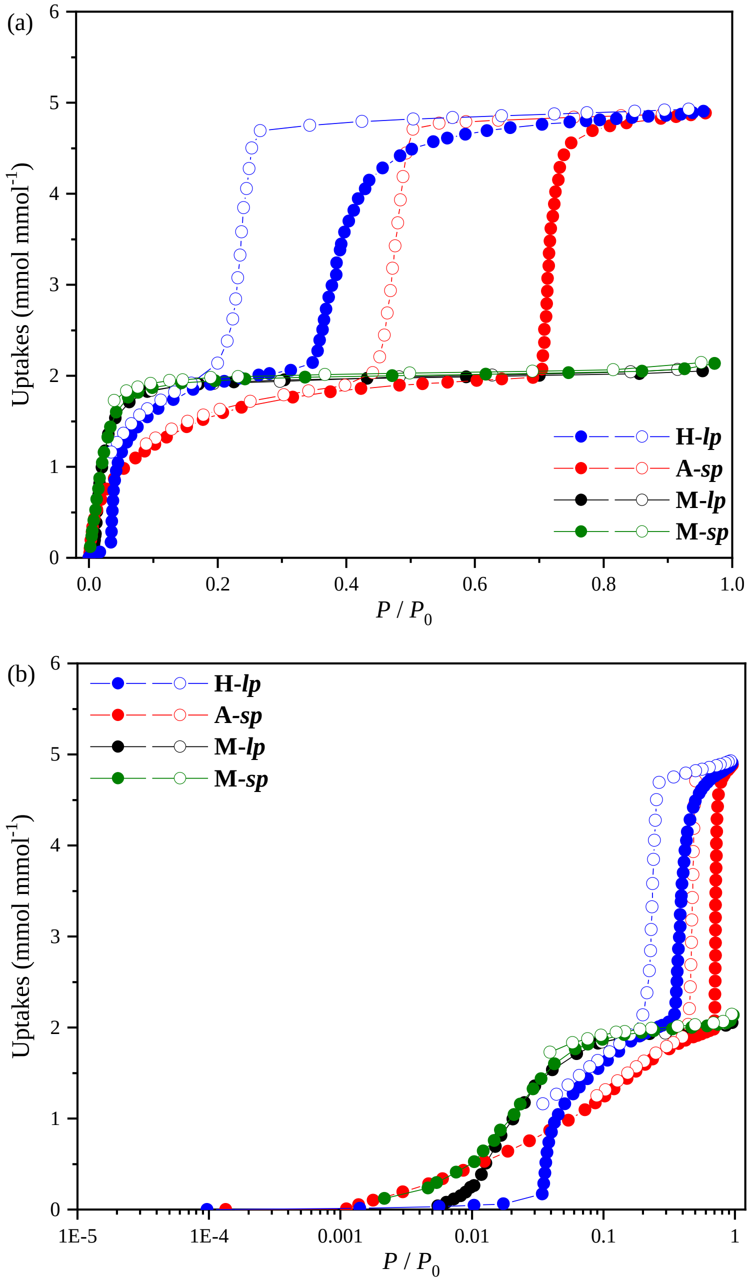


**Figure S14.** $H_2O$ adsorption (solid) and desorption (open) isotherms at 298 K. (a) Linear abscissa. (b) Logarithmic abscissa. The isotherms of **M-*sp*** superimposed with those of **M-*lp***, except for the absence of an inflection point at low pressure because there was no **M-*lp***-to-**M-*sp*** transformation. At high pressures ($P/P_0 > 0.2$), the second isotherm plateau indicated that **H-*sp*** and **A-*sp*** can expand to **H-*lp*** and **A-*lp***, respectively, following a framework expansion trend of **H** > **A**. According the framework contraction/expansion trends, **M-*sp*** is expected to expand to **M-*lp*** at a pressure between those of **H** and **A**. However, the isotherm and additional adsorption experiments showed that **M-*sp*** cannot transform to **M-*lp*** even in liquid water (Supplementary Figure 5), which could be attributed to the relatively high hydrophobicity of **M** functionalized by the $–CH_3$ group.

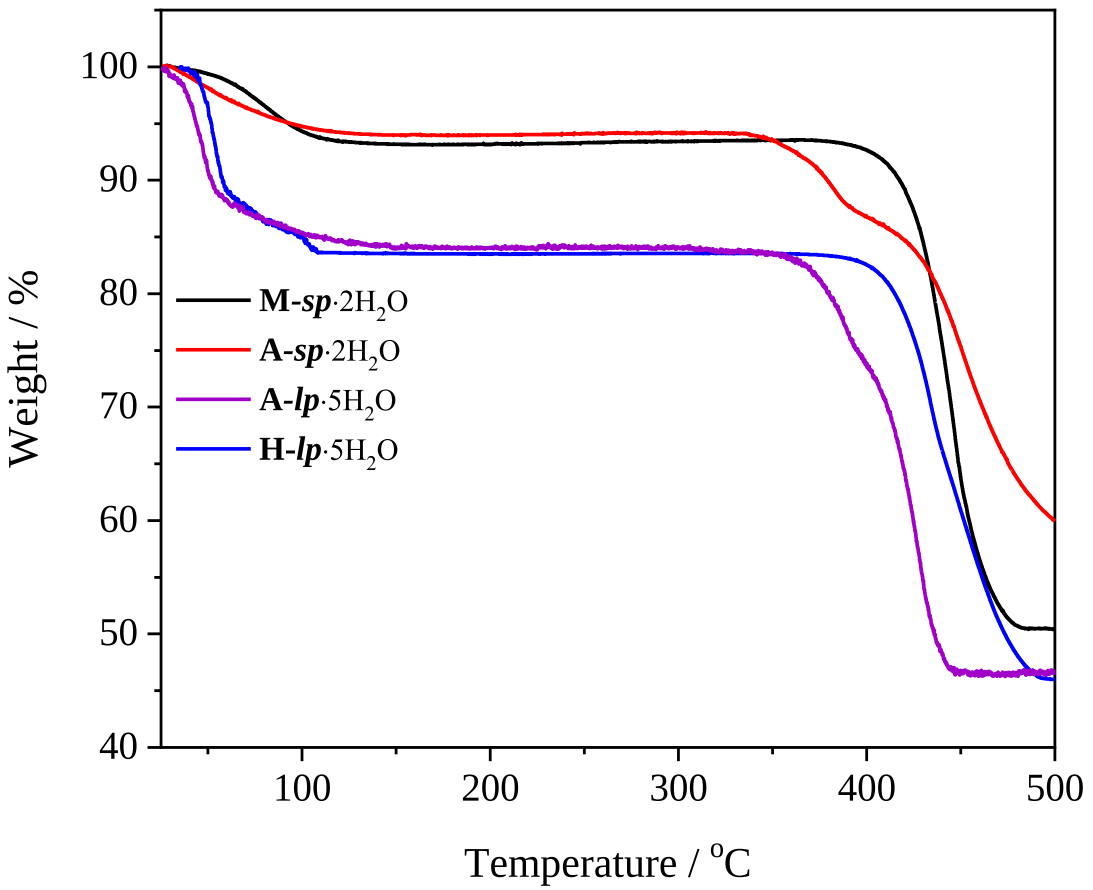


**Figure S15.** TG curves of the $H_2O$-adsorbed samples. **H-*lp*** $5H_2O$, **A-*sp*** $2H_2O$, **A-*lp*** $5H_2O$, and **M-*sp*** $2H_2O$ showed weight losses of 16.5%, 6.1%, 16.0%, and 6.8% below 150 °C. The theoretical weight losses for removal of 5.0, 2.0, 5.0, 2.0 $H_2O$ molecules per formula unit in these samples can be calculated from the crystal structures as 16.6%, 7.2%, 16.2%, and 7.2%, respectively. Further, **H-*lp*** $5H_2O$/**A-*lp*** $5H_2O$ showed two-step dehydration with weight losses of 9.9%/9.6% and 6.6%/6.4%, corresponding to the removal of 3 and 2 $H_2O$ molecules per formula unit (theoretical weight losses of 10.0%/9.7% and 6.6%/6.5%), respectively. As shown in the $H_2O$ adsorption isotherms, **H-*sp*** $2H_2O$ is not stable in air and **M-*lp*** $5H_2O$ does not existed, so that they cannot be used for TG analyses.

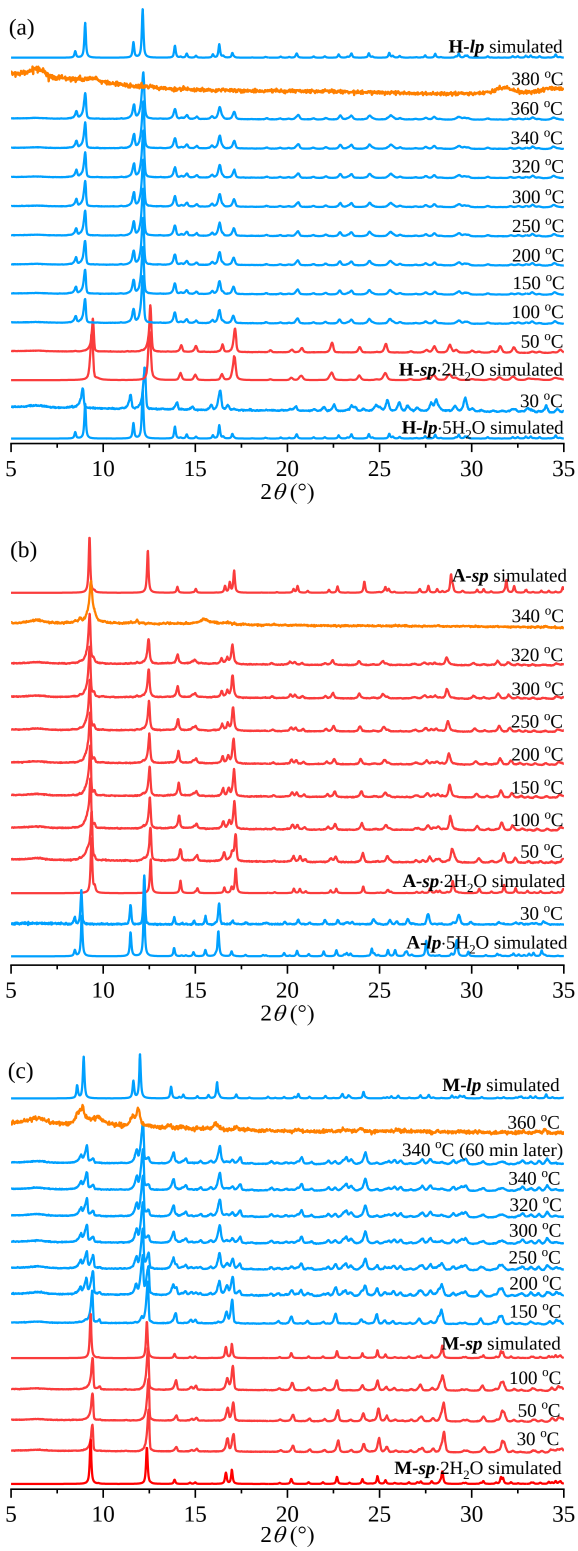


**Figure S16.** *In-situ* variable-temperature PXRD patterns of (a) **H-*lp*** $5H_2O$, (b) **A-*sp*** $2H_2O$, and (c) **M-*sp*** $2H_2O$.

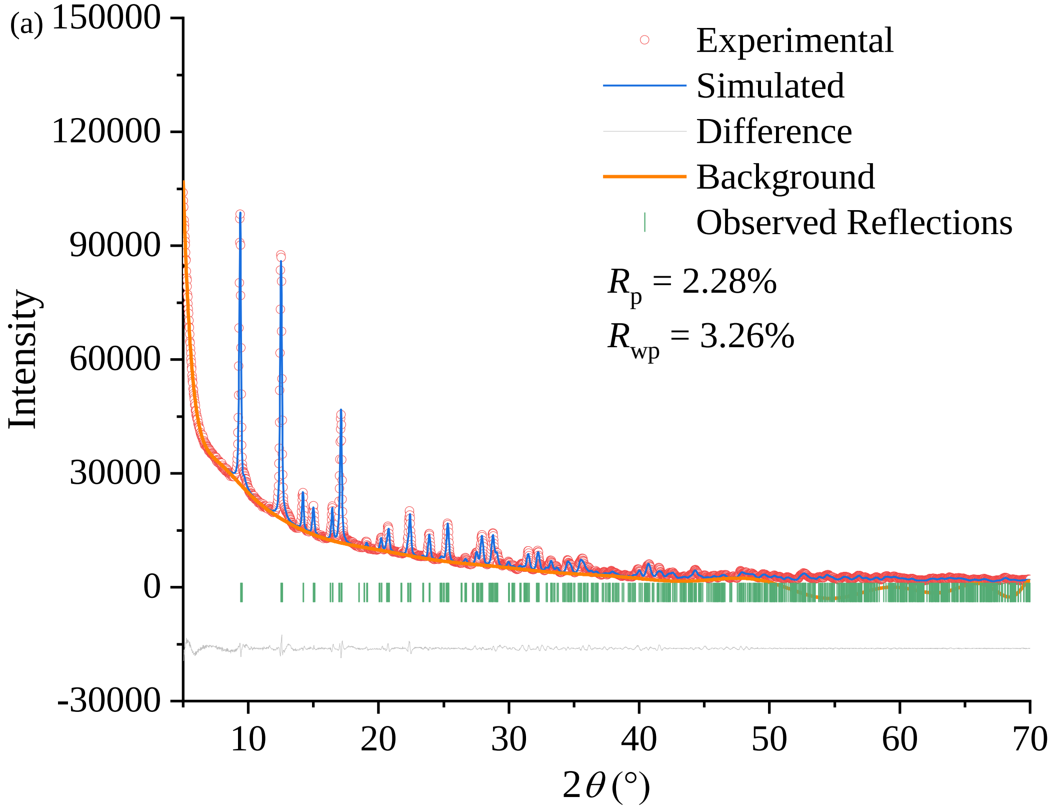


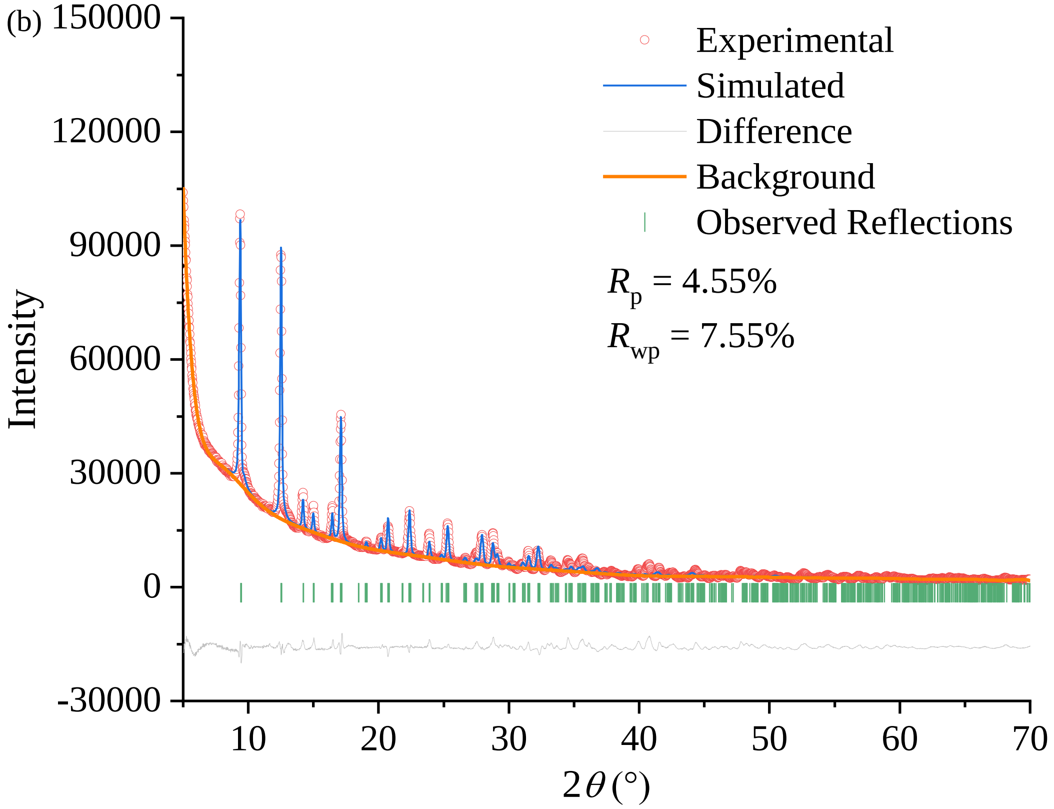


**Figure S17.** (a) Pawley and (b) Rietveld refinements for **H-*sp***$\cdot 2H_2O$.

(a)

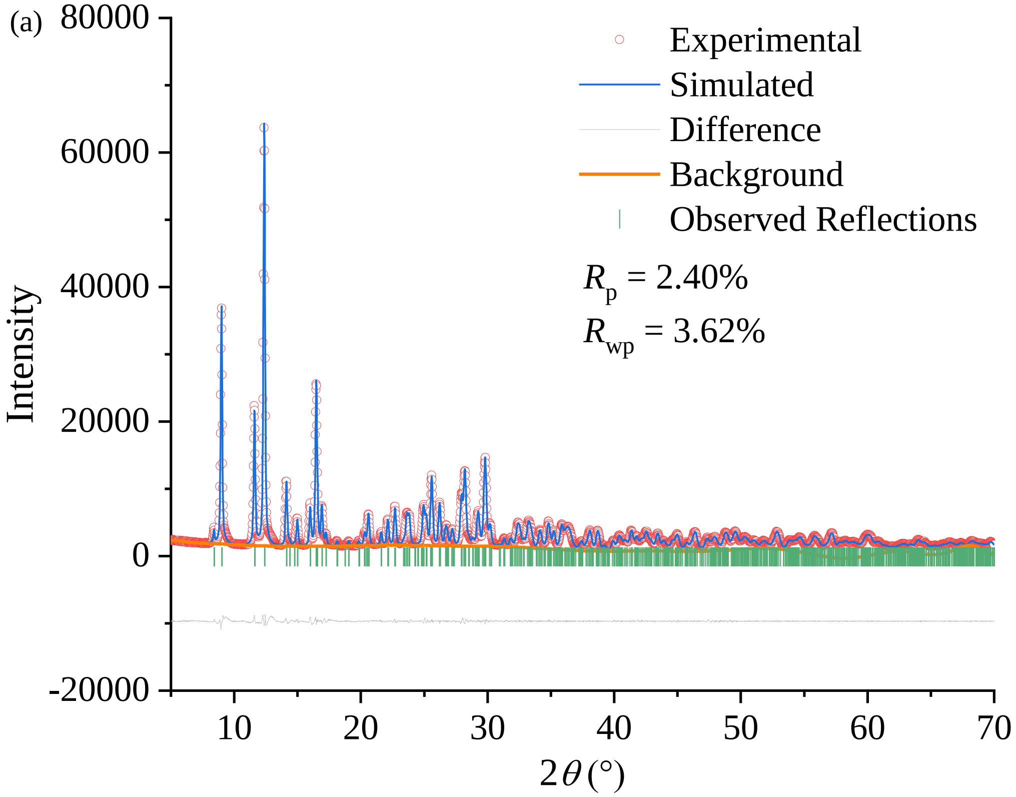


(b)

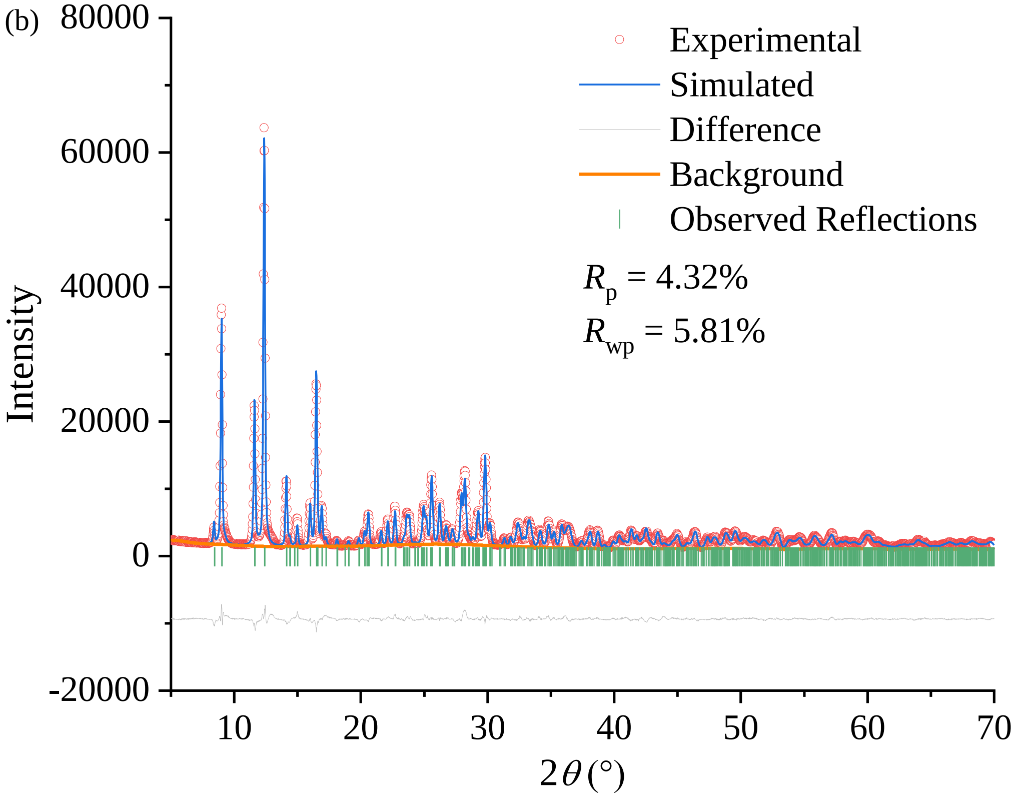


**Figure S18.** (a) Pawley and (b) Rietveld refinements for **H-*lp***·$5H_2O$.

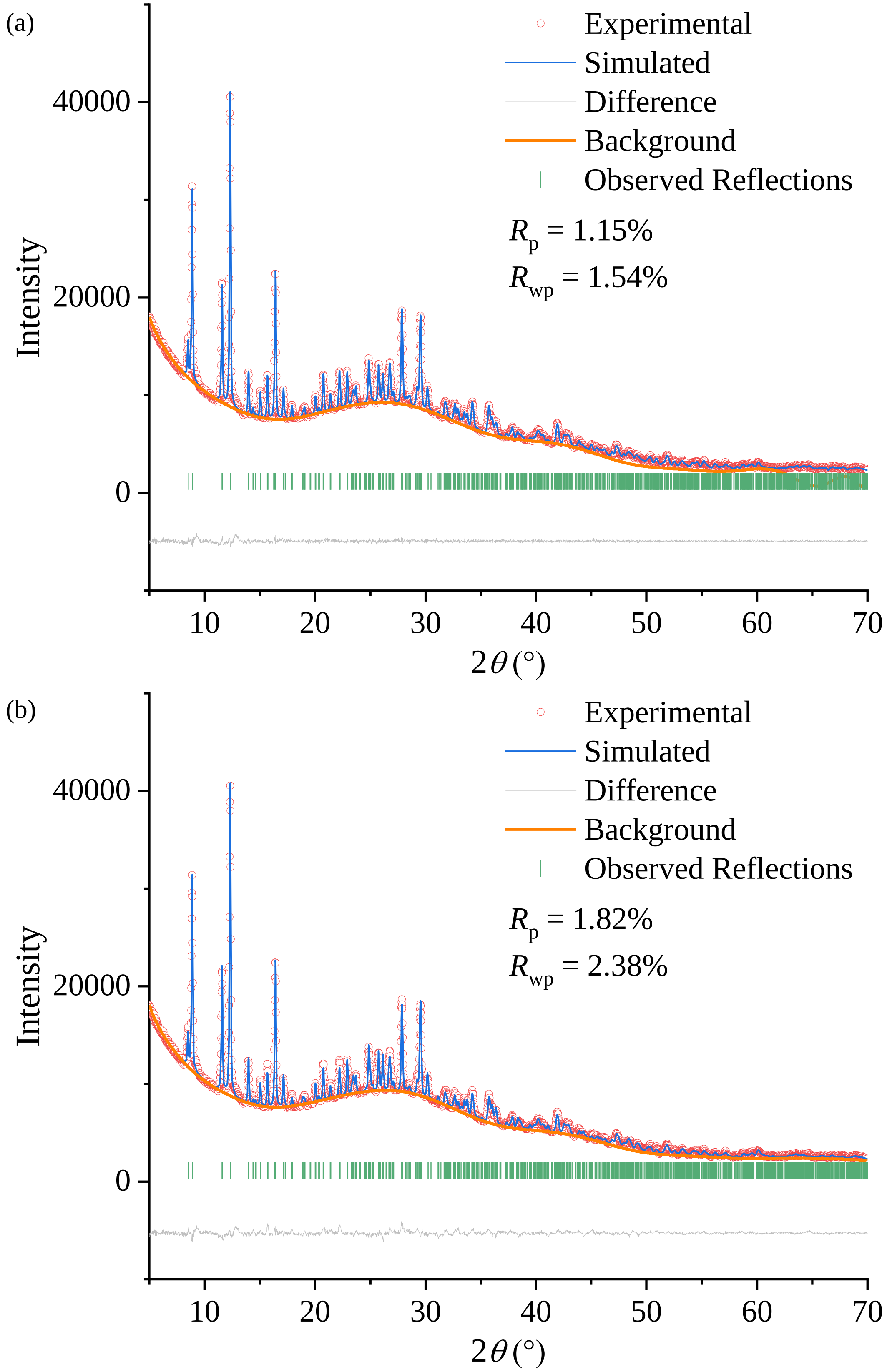


**Figure S19.** (a) Pawley and (b) Rietveld refinements for **A-*lp***$\cdot 5H_2O$.

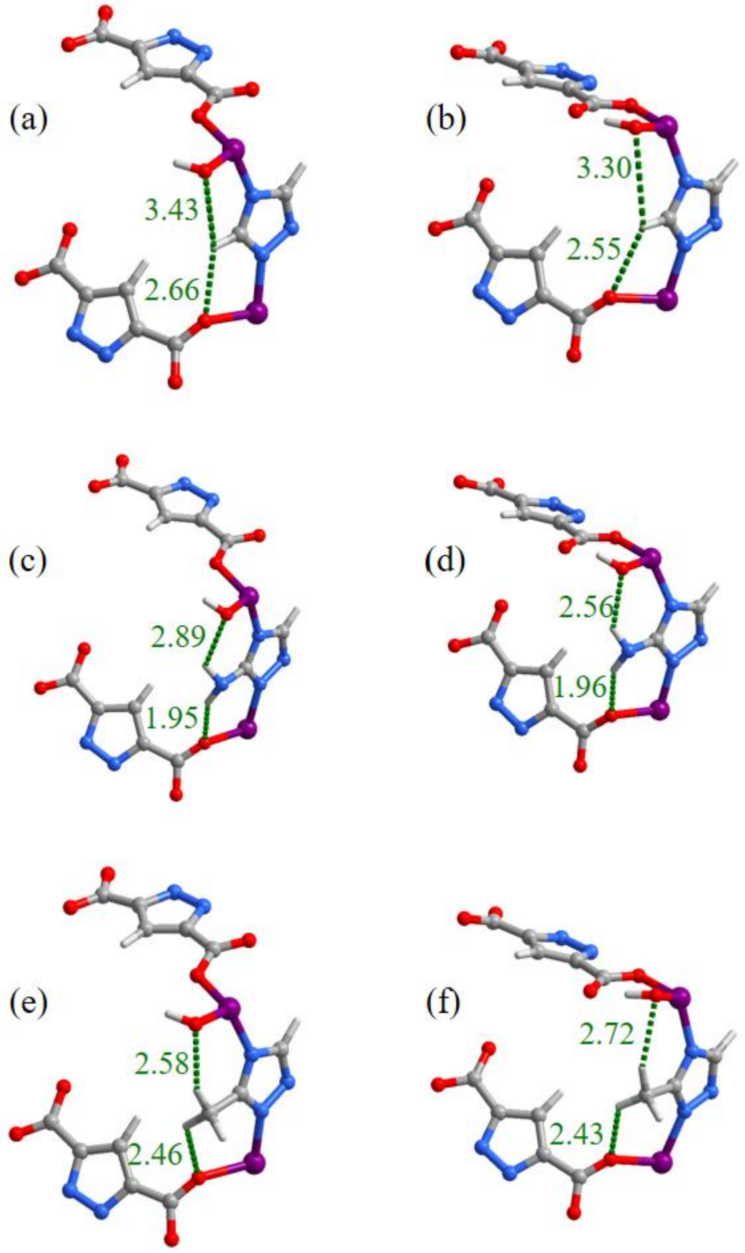


**Figure S20.** Comparison of the hydrogen-bonding distances. (a) **H-*lp***, (b) **H-*sp***, (c) **A-*lp***, (d) **A-*sp***, (e) **M-*lp***, (f) **M-*sp***. The hydrogen-bonding distances in **A** are much shorter than those in **H** and **M** (strong hydrogen-bonding ability of $–NH_2$), especially in the *sp* phase (strong attractive interactions to lower the energy of the *sp* phase and inverse the relative stability of the *sp*/*lp* phases). These distances in **H** are all very long (weak hydrogen-bonding ability of aromatic –H and small size of –H), meaning that hydrogen-bonding interactions are negligible (without hydrogen bonding interaction, the *lp* phase of **H**/**A**/**M** should be thermodynamically more stable). For **M**, these distances are still long (weak hydrogen-bonding ability of $–CH_3$), but should provide non-negligible hydrogen-bonding interactions to lower the energy of the *sp* phase.

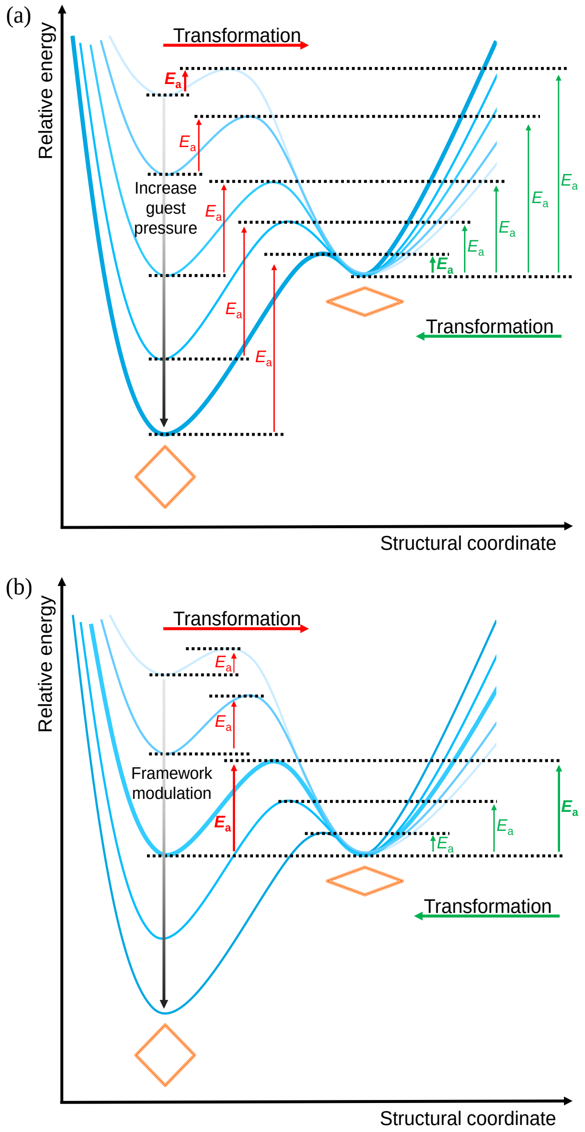


**Figure S21.** Typical evolution trends of the energy profile of a bistable system. (a) Evolution of the energy profile of a host-guest system by changing guest pressure. For simplicity, *lp* and *sp* states here are porous and nonporous for the guest, respectively (pore-opening/closing transformations). During the adsorption process, as the guest pressure increases, $\Delta E$ and $E_a$ from *sp* to *lp* gradually decrease. During the desorption process, as the guest pressure decreases, $\Delta E$ and $E_a$ from *lp* to *sp* also gradually decrease. Structural transformations always occur at $\Delta E < 0$ and $E_a < xkT$. (b) Evolution of the energy profile of a framework by isostructural modulation. Without external stimulus, spontaneous transformation decreases the system energy, for which the energy barrier reaches its maximum when the energy difference between the two states is zero.

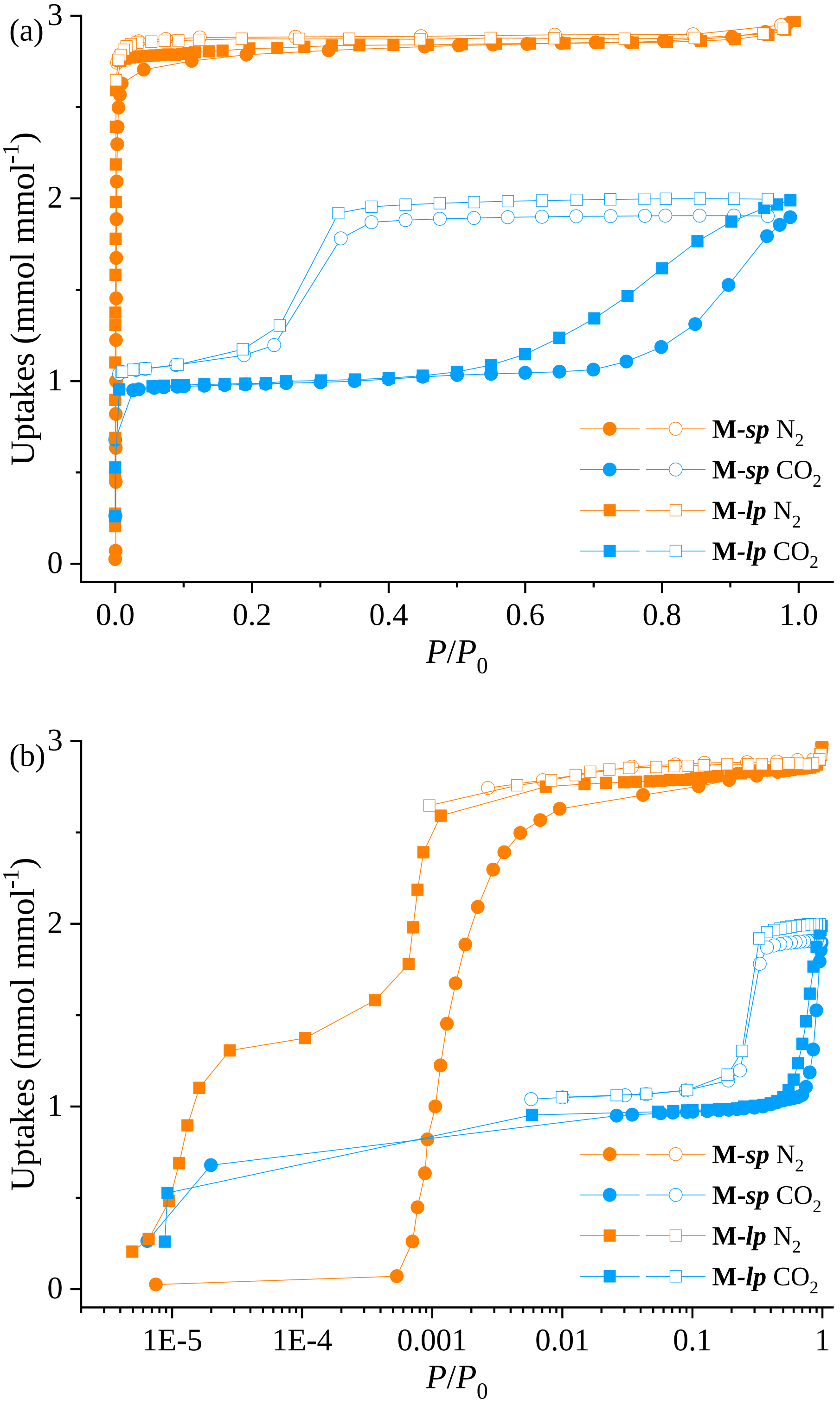


**Figure S22.** $N_2$ (77 K) and $CO_2$ (195 K) adsorption/desorption isotherms of **M-*sp*** and **M-*lp***. (a) Linear and (b) logarithmic abscissa.

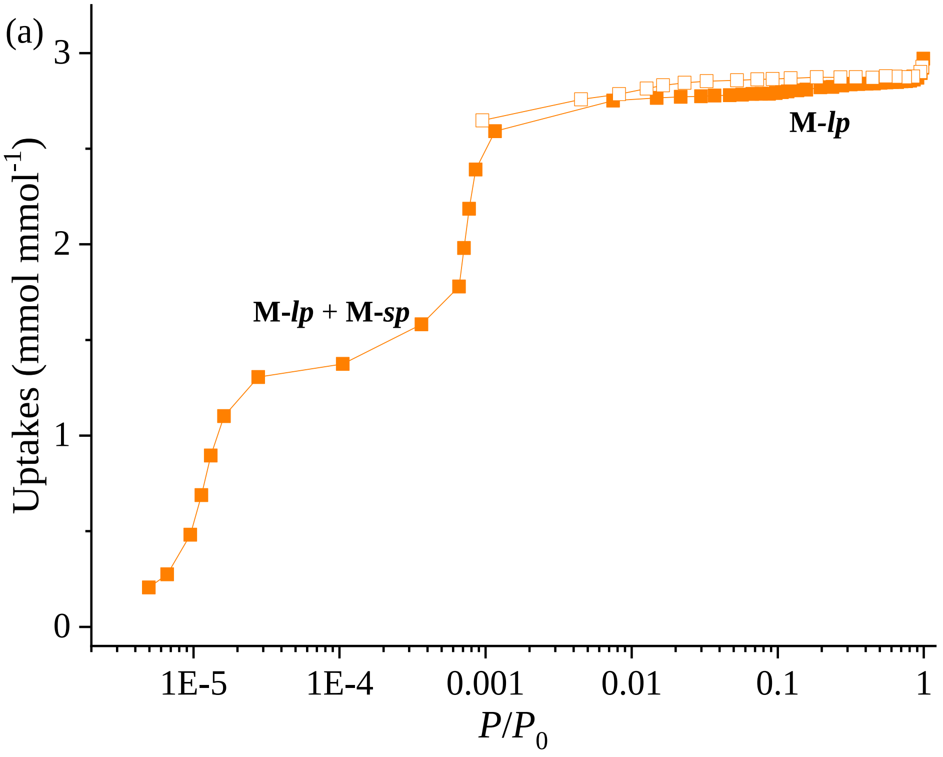


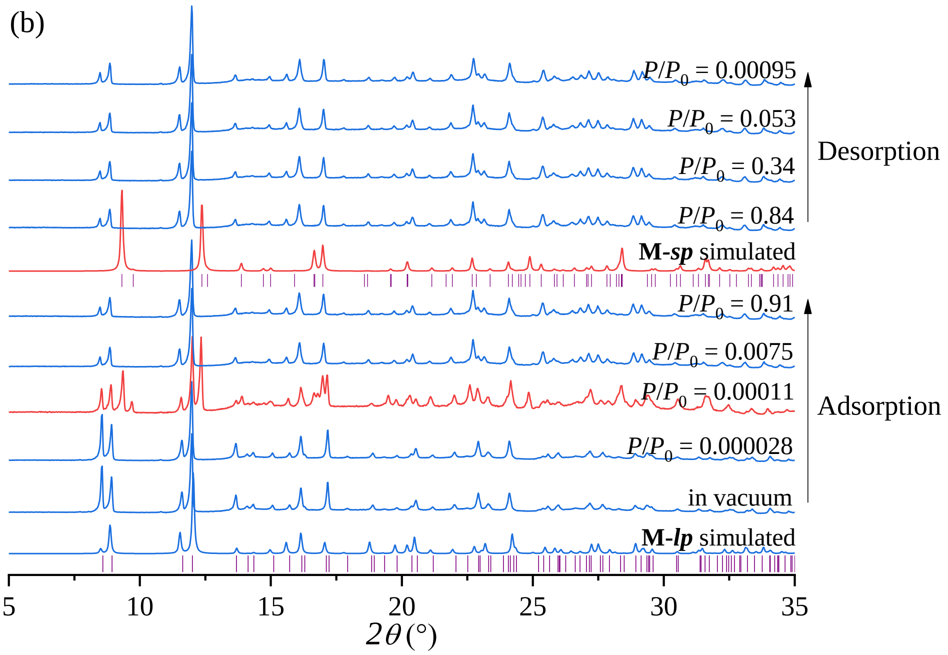


**Figure S23.** *In-situ* PXRD patterns of **M-*lp*** in $N_2$ at 77 K. (a) Adsorption and desorption isotherms. (b) PXRD patterns at equilibrium conditions. Complete desorption was not achieved here due to limitations of the measurement principle. Note that the room-temperature high-pressure adsorption/desorption experiments in Figure S49 confirmed that the *lp* state in $N_2$ can be maintained after complete $N_2$ desorption.

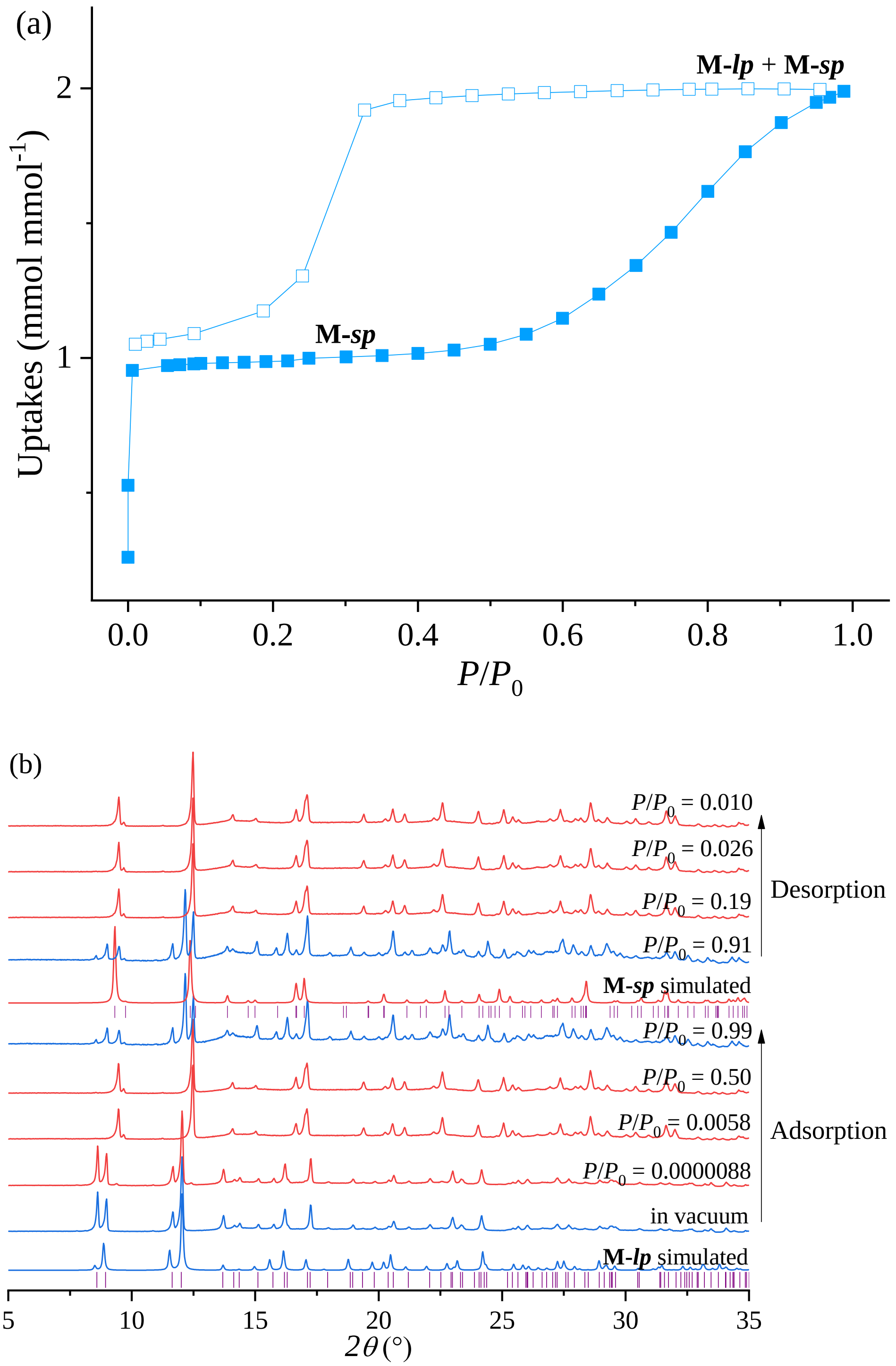


**Figure S24.** *In-situ* PXRD patterns of **M-*lp*** in $CO_2$ at 195 K. (a) Adsorption and desorption isotherms. (b) PXRD patterns at equilibrium conditions. Complete desorption was not achieved here due to limitations of the measurement principle. Note that the room-temperature high-pressure adsorption/desorption experiments in Figure S50 confirmed that the *sp* state in $CO_2$ can be maintained after complete $CO_2$ desorption.

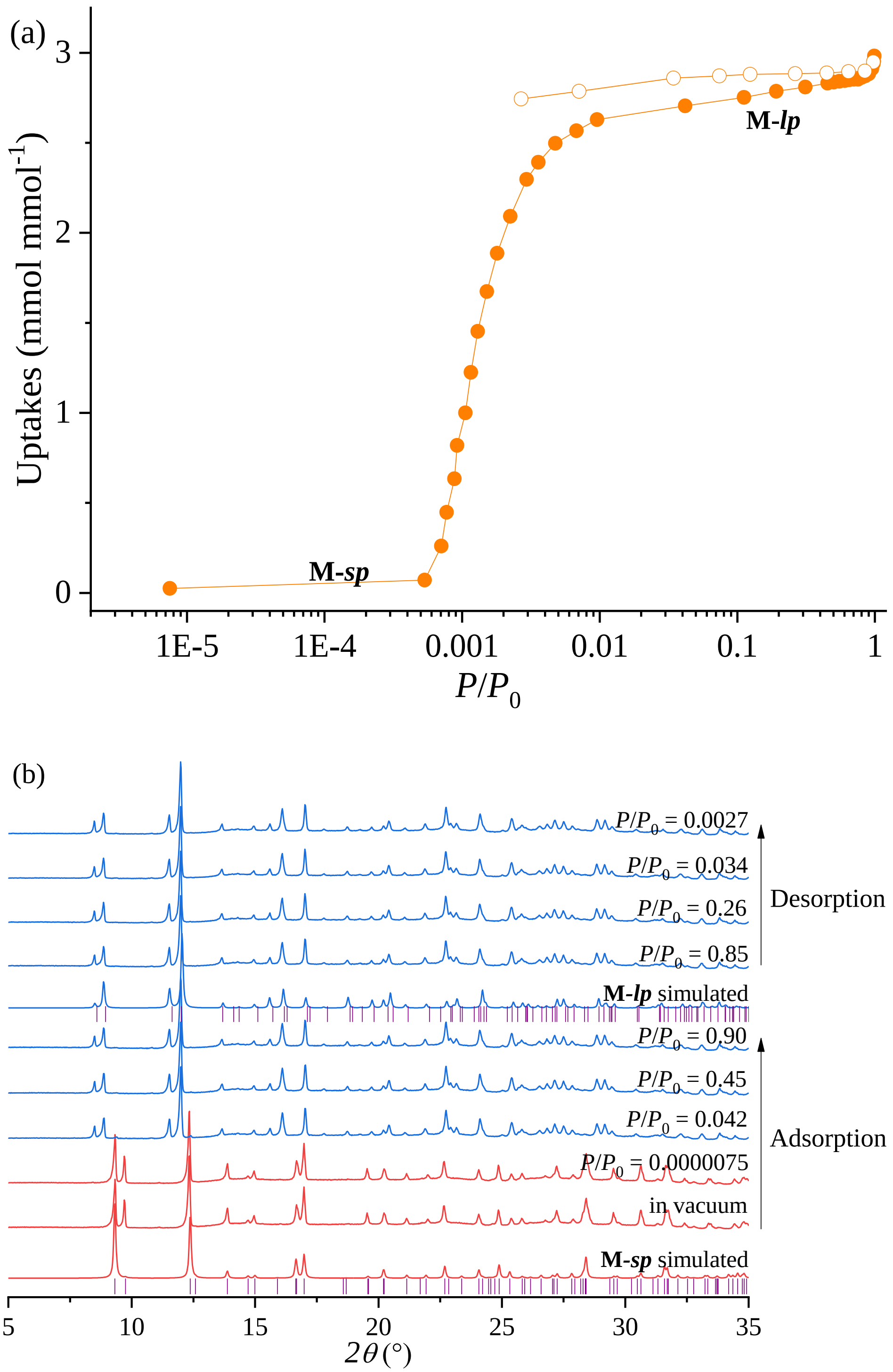


**Figure S25.** *In-situ* PXRD patterns of **M-*sp*** in $N_2$ at 77 K. (a) Adsorption and desorption isotherms. (b) PXRD patterns at equilibrium conditions. Complete desorption was not achieved here due to limitations of the measurement principle. Note that the room-temperature high-pressure adsorption/desorption experiments in Figure S49 confirmed that the *lp* state in $N_2$ can be maintained after complete $N_2$ desorption.

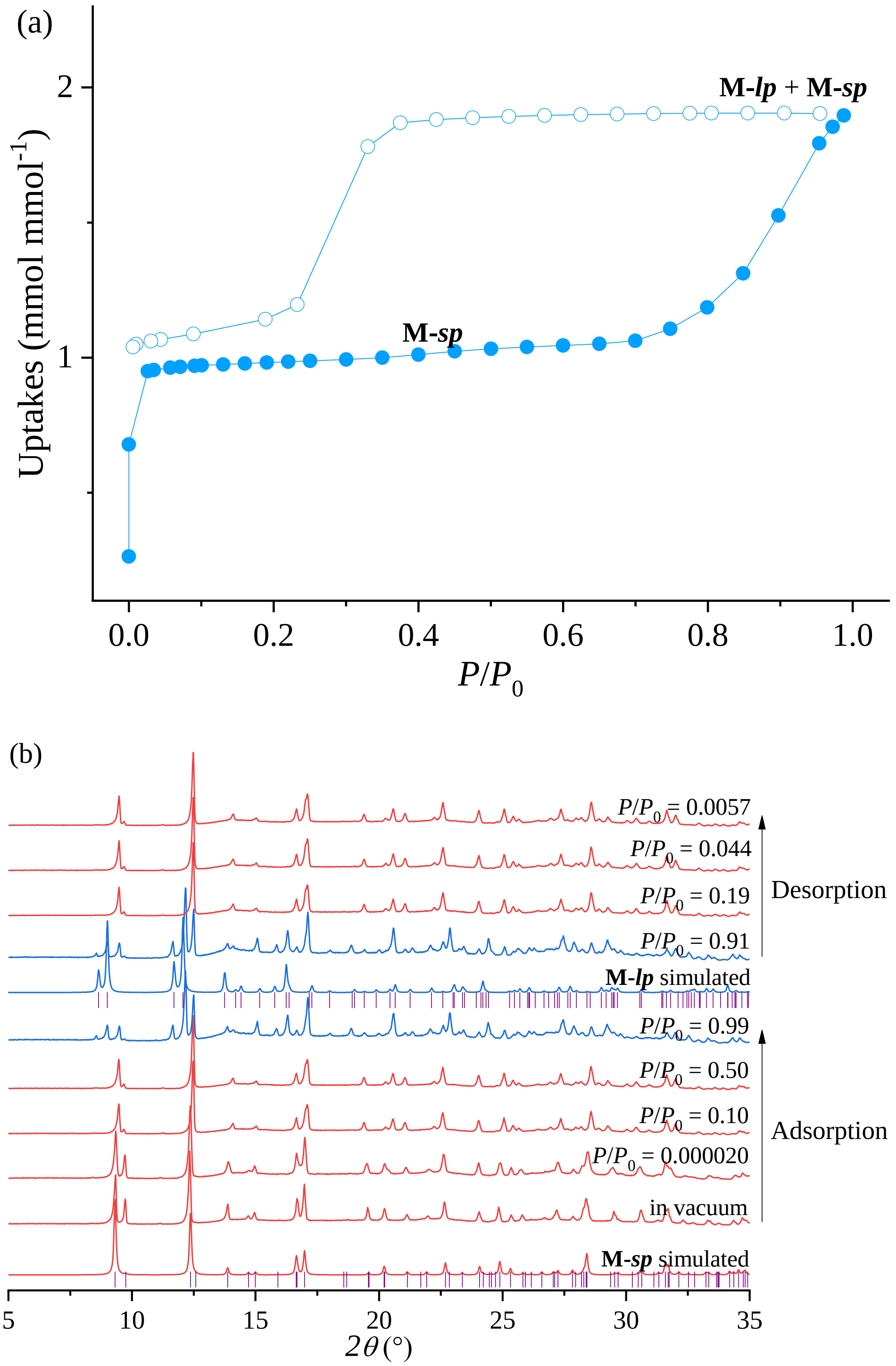


**Figure S26.** *In-situ* PXRD patterns of **M-*sp*** in $CO_2$ at 195 K. (a) Adsorption and desorption isotherms. (b) PXRD patterns at equilibrium conditions. Complete desorption was not achieved here due to limitations of the measurement principle. Note that the room-temperature high-pressure adsorption/desorption experiments in Figure S50 confirmed that the *sp* state in $CO_2$ can be maintained after complete $CO_2$ desorption.

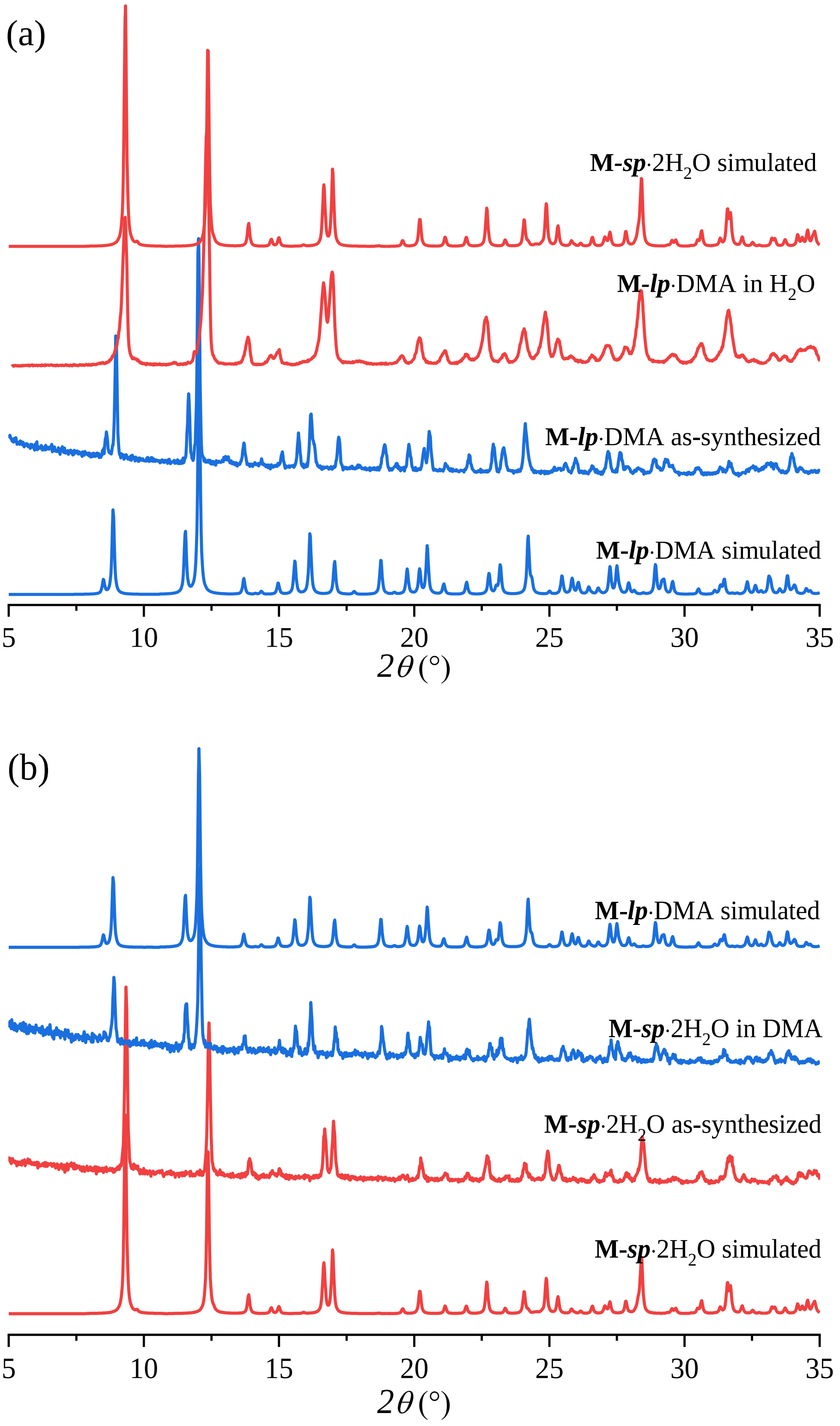


**Figure S27.** PXRD patterns of (a) **M-*lp*** ·DMA and (b) **M-*sp*** ·$2H_2O$ soaked in water and DMA, respectively.

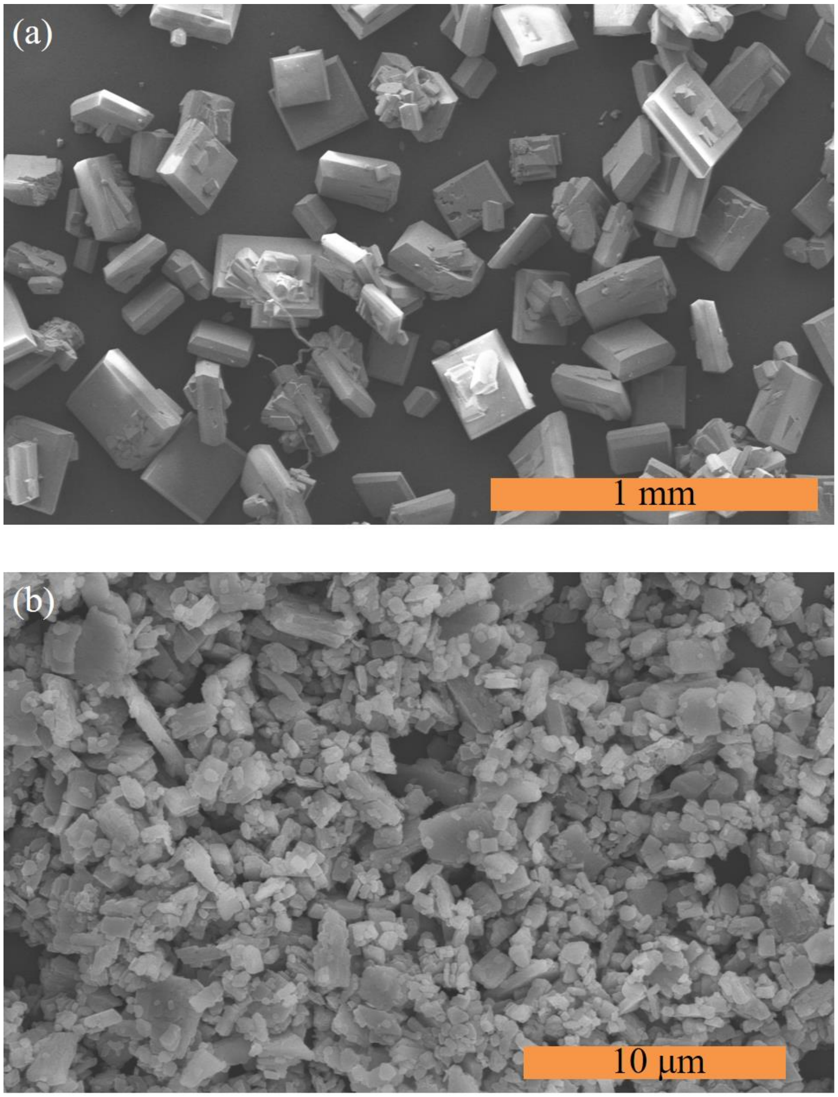


**Figure S28.** SEM images of **M-*lp***·DMA (a) before and (b) after repeated solvent exchange.

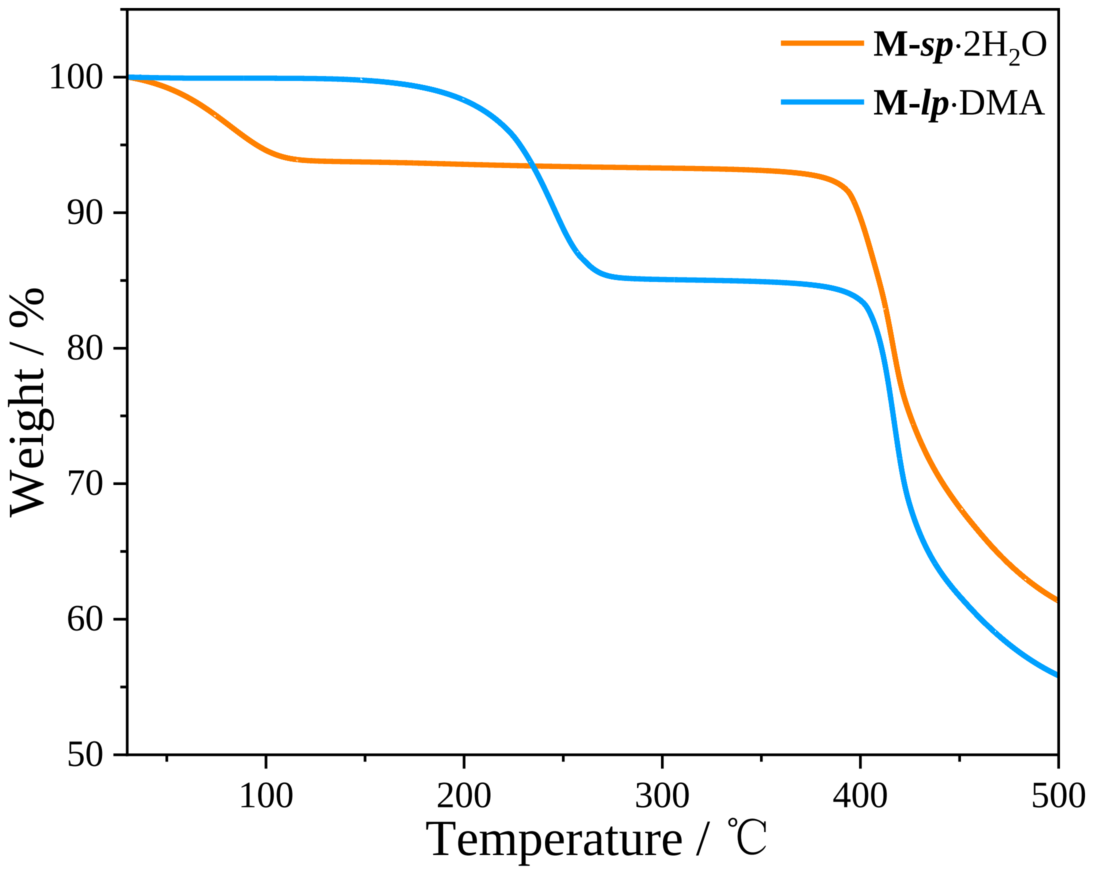


**Figure S29.** TG curves of the small-sized **M-*sp*** ·$2H_2O$ and **M-*lp*** ·DMA.

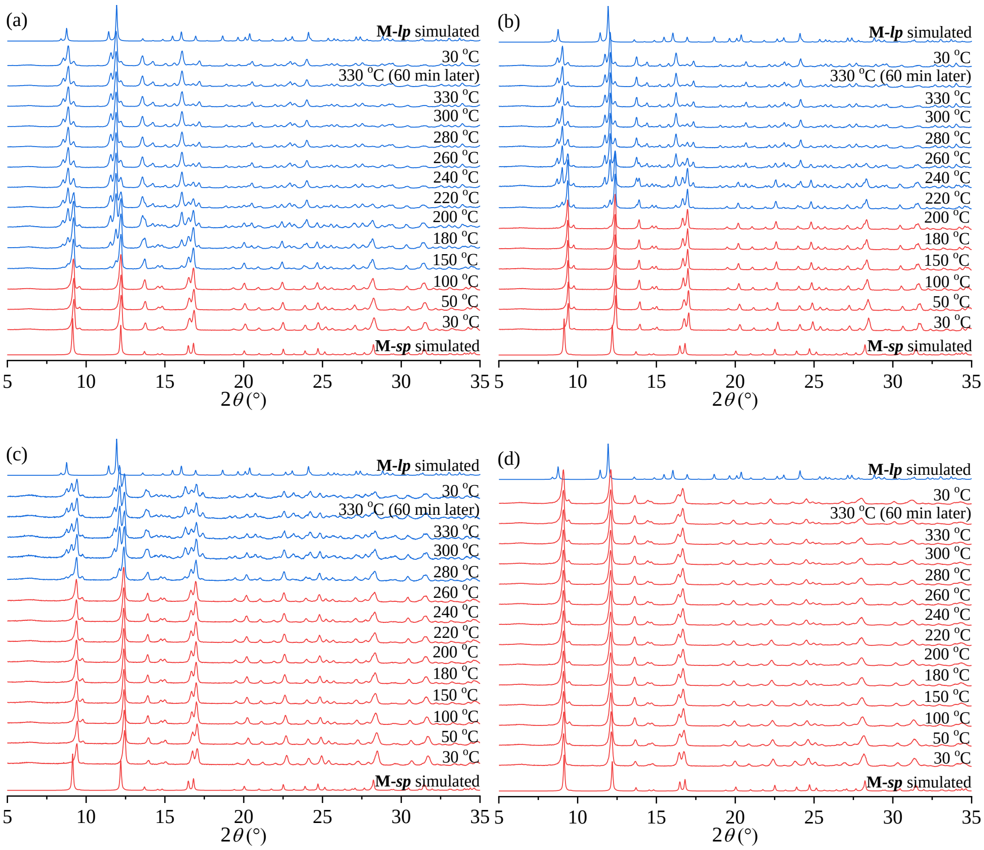


**Figure S30.** *In-situ* variable-temperature PXRD patterns of **M-*sp*** ·2$H_2O$ (a) before and after (b) 5, (c) 10 cycles and (d) 20 cycles of DMA adsorption/desorption and $H_2O$ adsorption/desorption in turn.

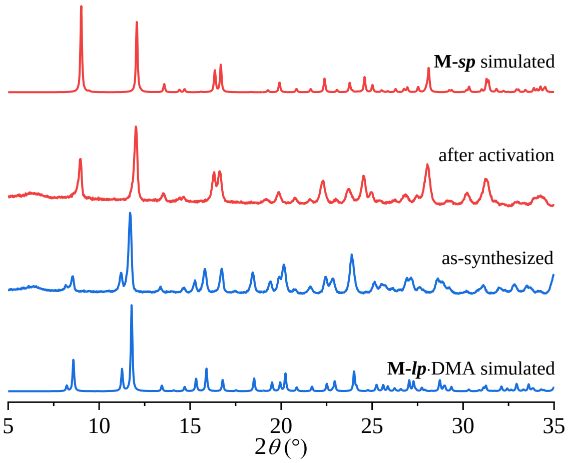


**Figure S31.** PXRD patterns of the small-sized **M-*lp*** ·DMA before and after activation. The spontaneous transformation after guest removal indicates that the small-sized sample is thermodynamically stable in the *sp* phase and lose the plastic-pore characteristics, exhibiting only the common elastic-pore behavior (for solvent molecule).

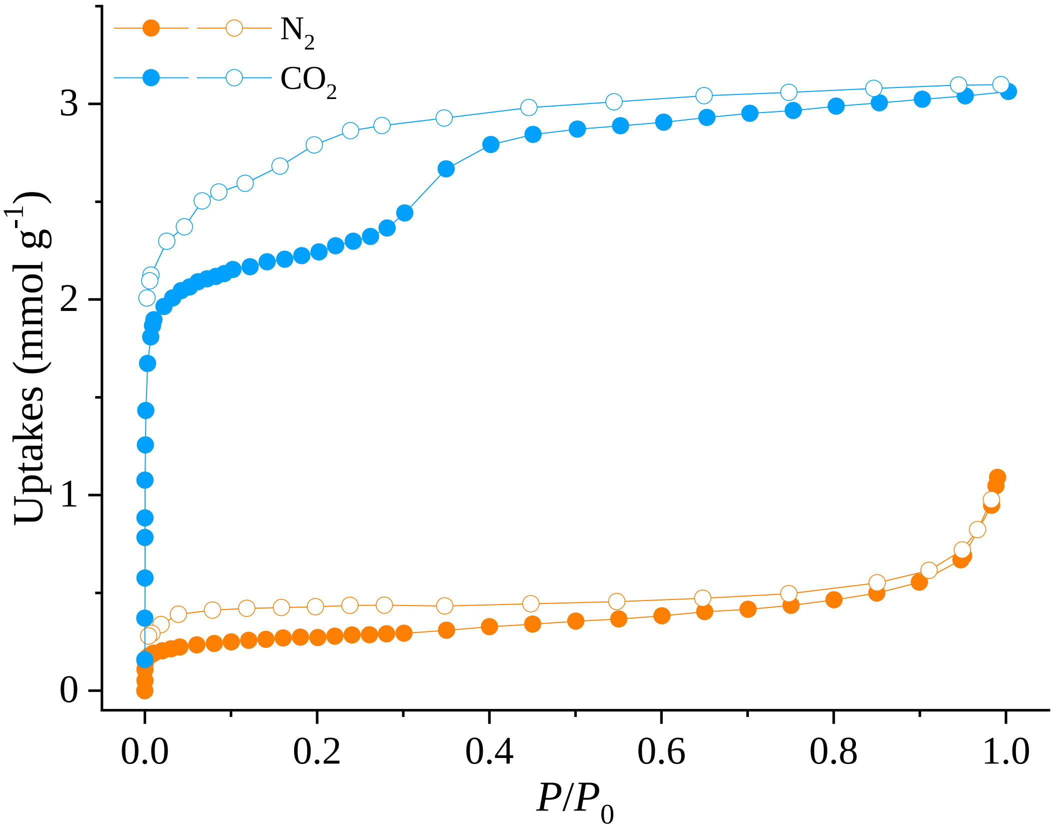


**Figure S32.** 77 K $N_2$ and 195 K $CO_2$ adsorption and desorption isotherms of the small-sized **M-*sp***. The $CO_2$ adsorption capacity at the first platform is 2.2 mmol $g^{-1}$, which is consistent with that of **M-*sp*** (2.2 mmol $g^{-1}$). $N_2$ and $CO_2$ saturated uptakes are 0.5 and 2.9 mmol $g^{-1}$, respectively, which are 0.4 and 0.6 mmol $g^{-1}$ higher than the corresponding uptakes of **M-*sp*** (0.1 and 2.3 mmol $g^{-1}$), respectively, which may be attributed to crystal defects generated by the repeated solvent exchange processes.

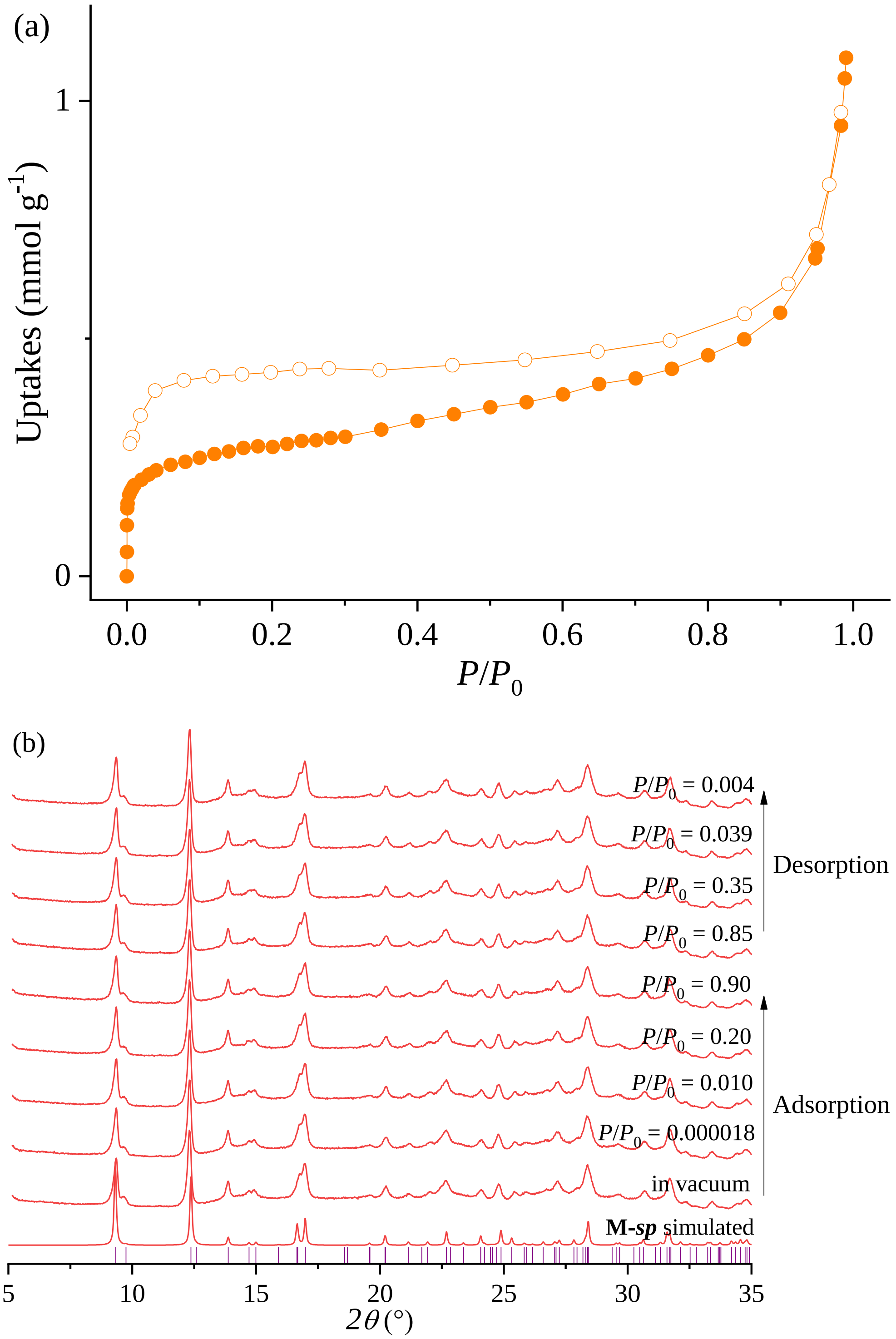


**Figure S33.** *In-situ* PXRD patterns of the small-sized **M-*sp*** in $N_2$ at 77 K. (a) Adsorption and desorption isotherms. (b) PXRD patterns at equilibrium conditions. This confirmed that low-temperature $N_2$ cannot induce the *sp*-to-*lp* transformation.

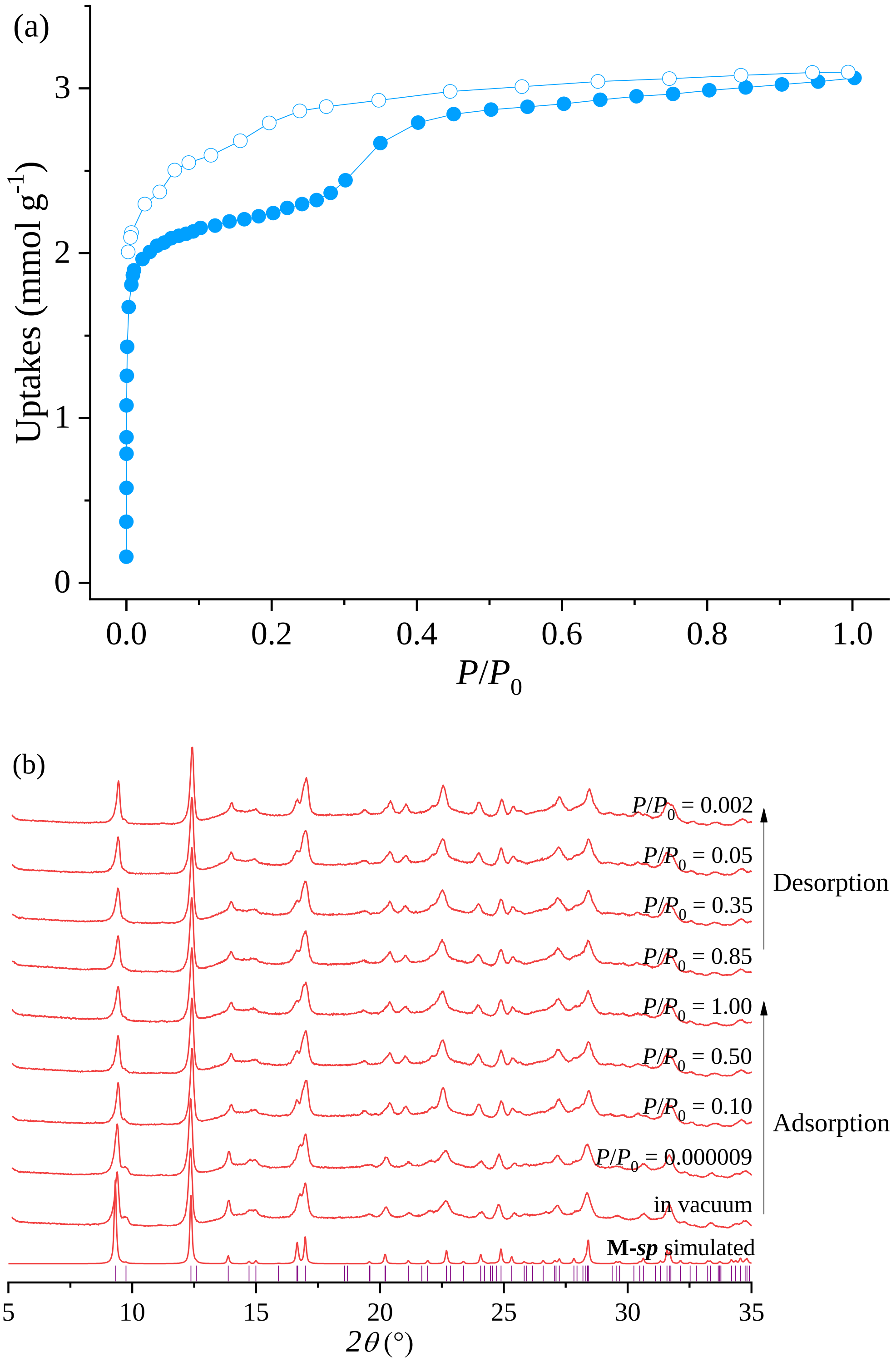


**Figure S34.** *In-situ* PXRD patterns of the small-sized **M-*sp*** in $CO_2$ at 195 K. (a) Adsorption and desorption isotherms. (b) PXRD patterns at equilibrium conditions. This confirmed that low-temperature $CO_2$ cannot induce the *sp*-to-*lp* transformation, indicating that the additional adsorption step arises from crystal defects.

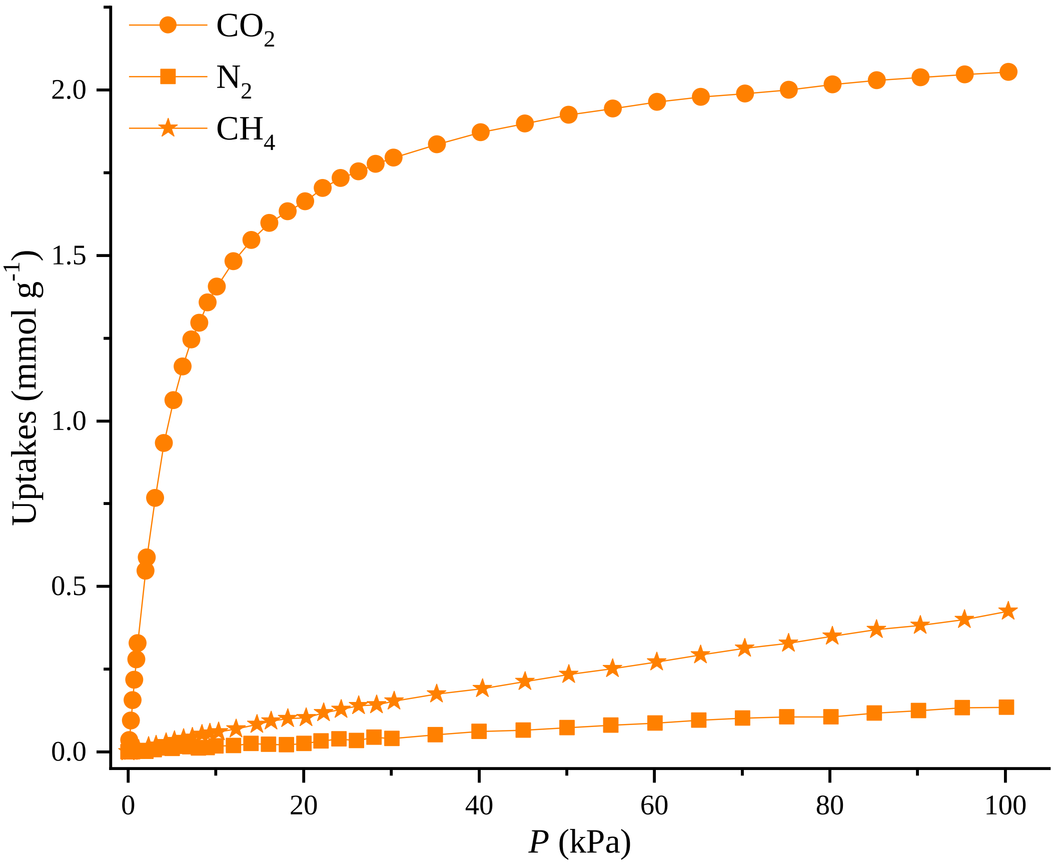


**Figure S35.** $N_2$, $CH_4$, and $CO_2$ adsorption isotherms of the small-sized **M-*sp*** at 298 K. All isotherms show type-I behavior, further indicating that the small-sized sample lose the plastic-pore characteristics. It becomes a rigid pore structure for these gases under the given condition.

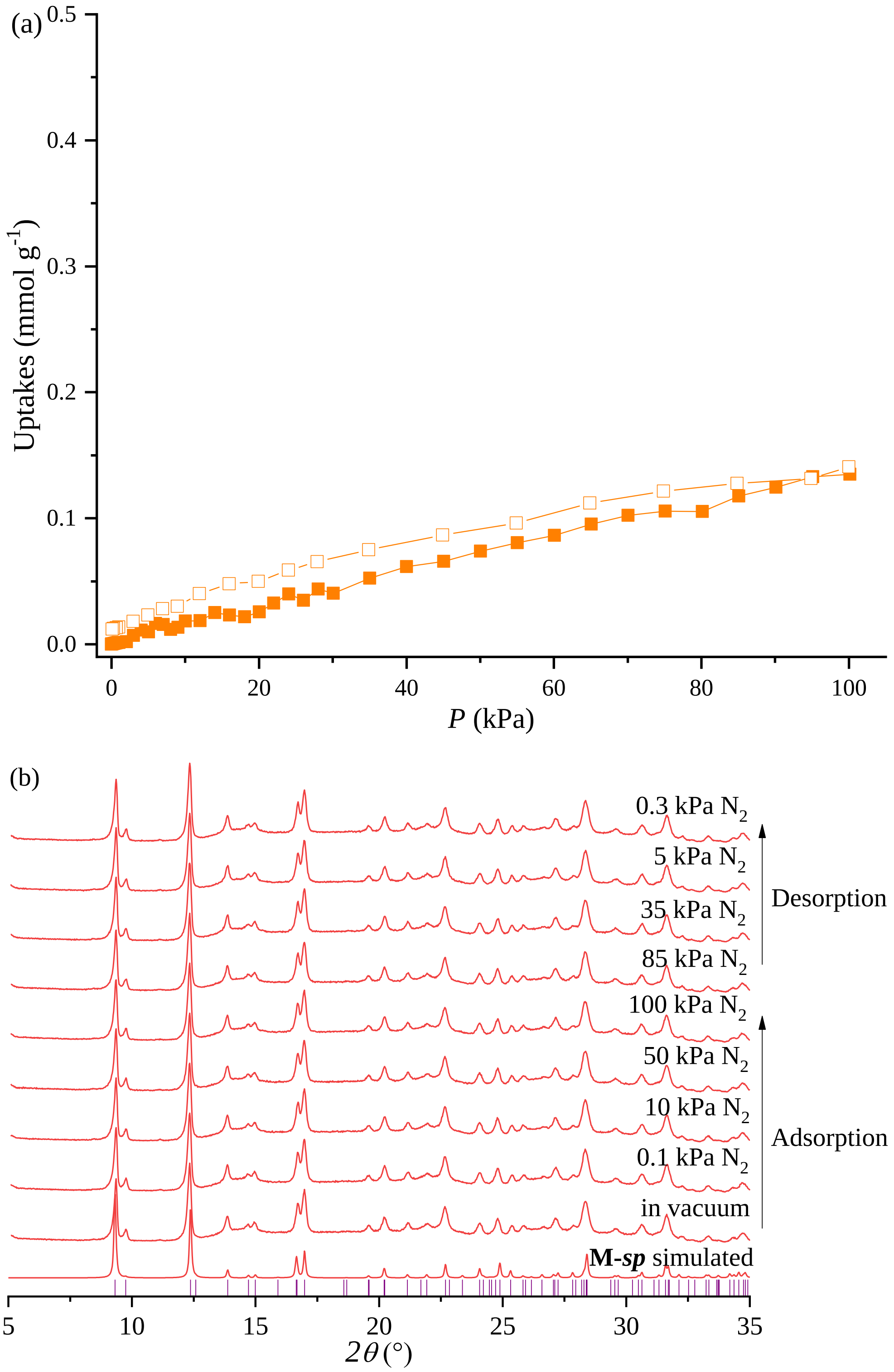


**Figure S36.** *In-situ* PXRD patterns of the small-sized ***M-sp*** in $N_2$ at 298 K. (a) Adsorption and desorption isotherms. (b) PXRD patterns at equilibrium conditions. This confirmed that, under the given condition, $N_2$ cannot induce the structural transformation.

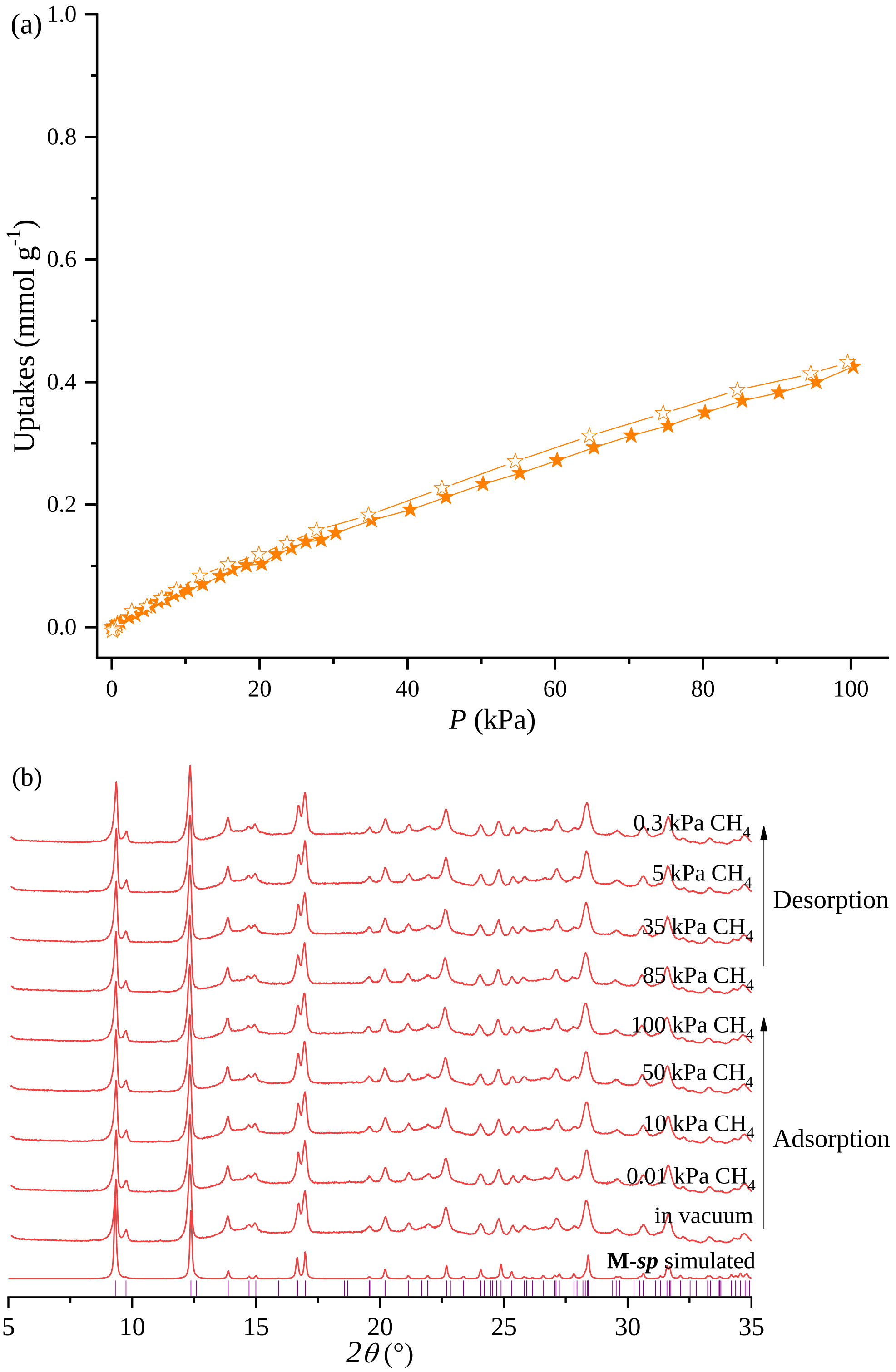


**Figure S37.** *In-situ* PXRD patterns of the small-sized **M-*sp*** in $CH_4$ at 298 K. (a) Adsorption and desorption isotherms. (b) PXRD patterns at equilibrium conditions. This confirmed that, under the given condition, $CH_4$ cannot induce the structural transformation.

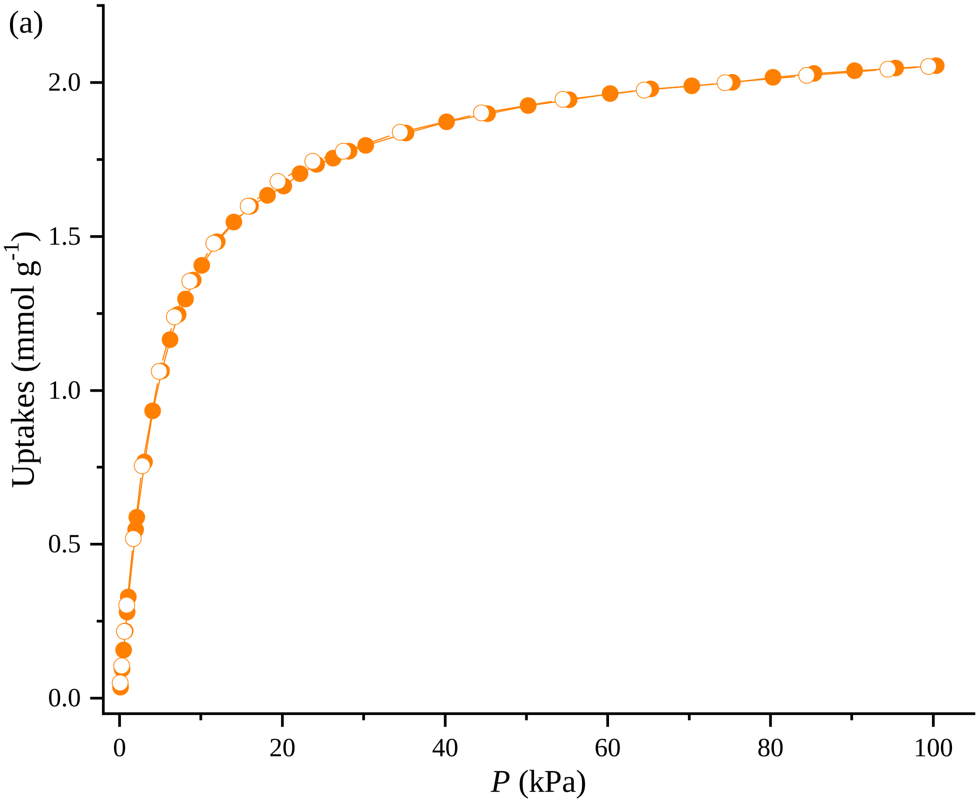


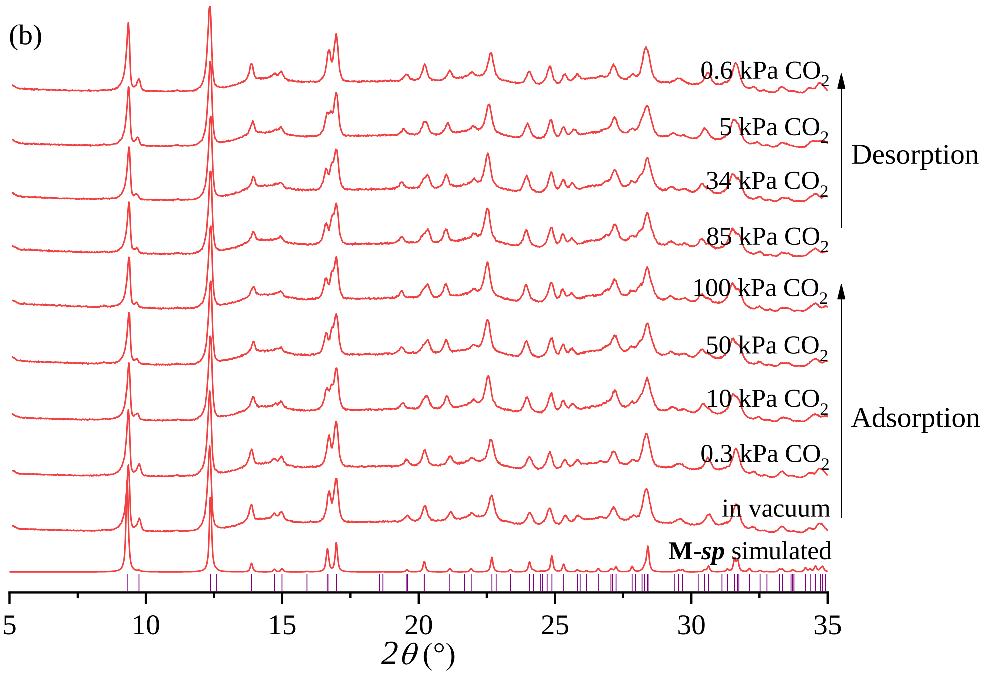


**Figure S38.** *In-situ* PXRD patterns of the small-sized **M-*sp*** in $CO_2$ at 298 K. (a) Adsorption and desorption isotherms. (b) PXRD patterns at equilibrium conditions. This confirmed that, under the given condition, $CO_2$ cannot induce the structural transformation.

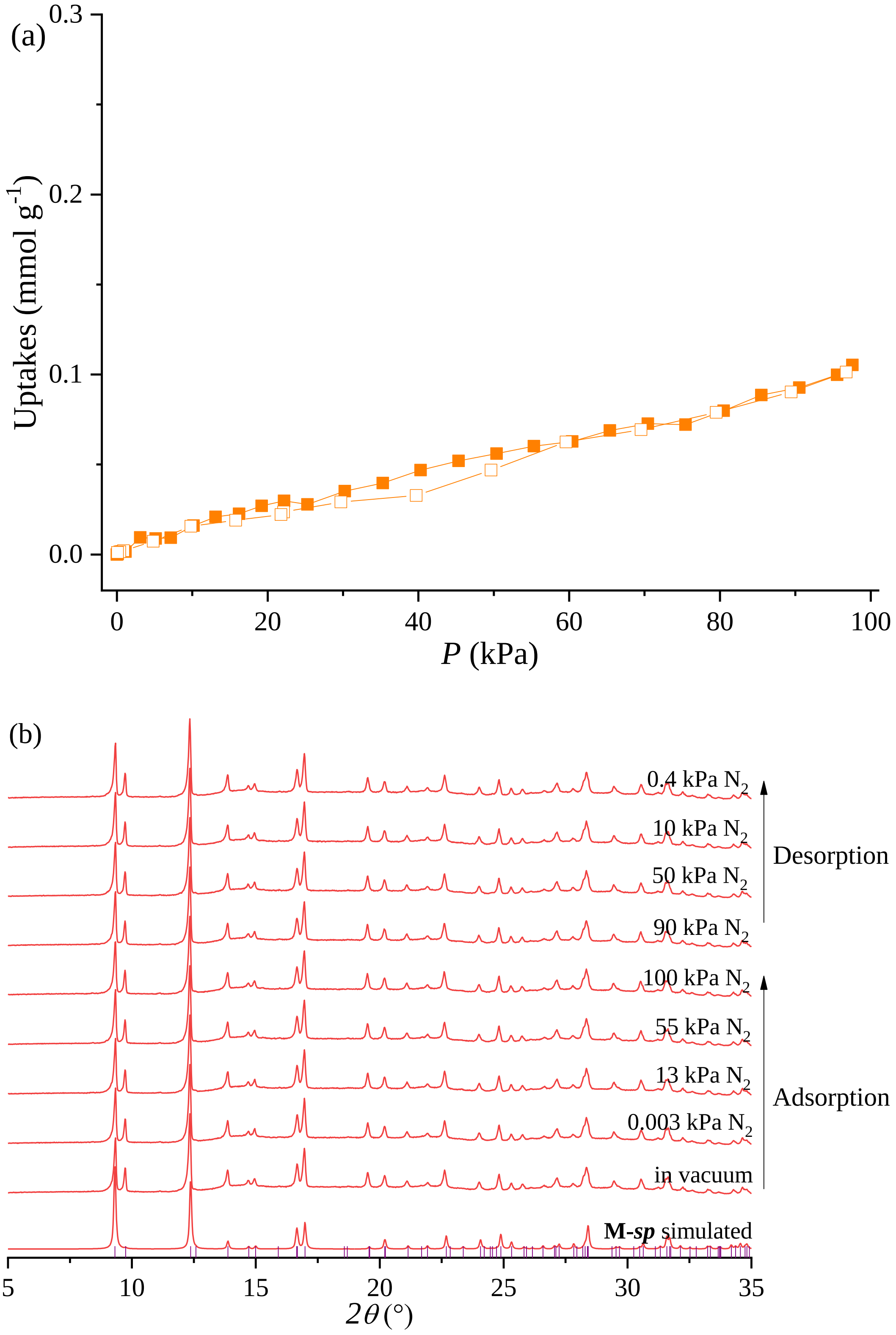


**Figure S39.** *In-situ* sorption-PXRD measurements of **M-*sp*** for $N_2$ at 298 K. (a) Adsorption and desorption isotherms. (b) PXRD patterns.

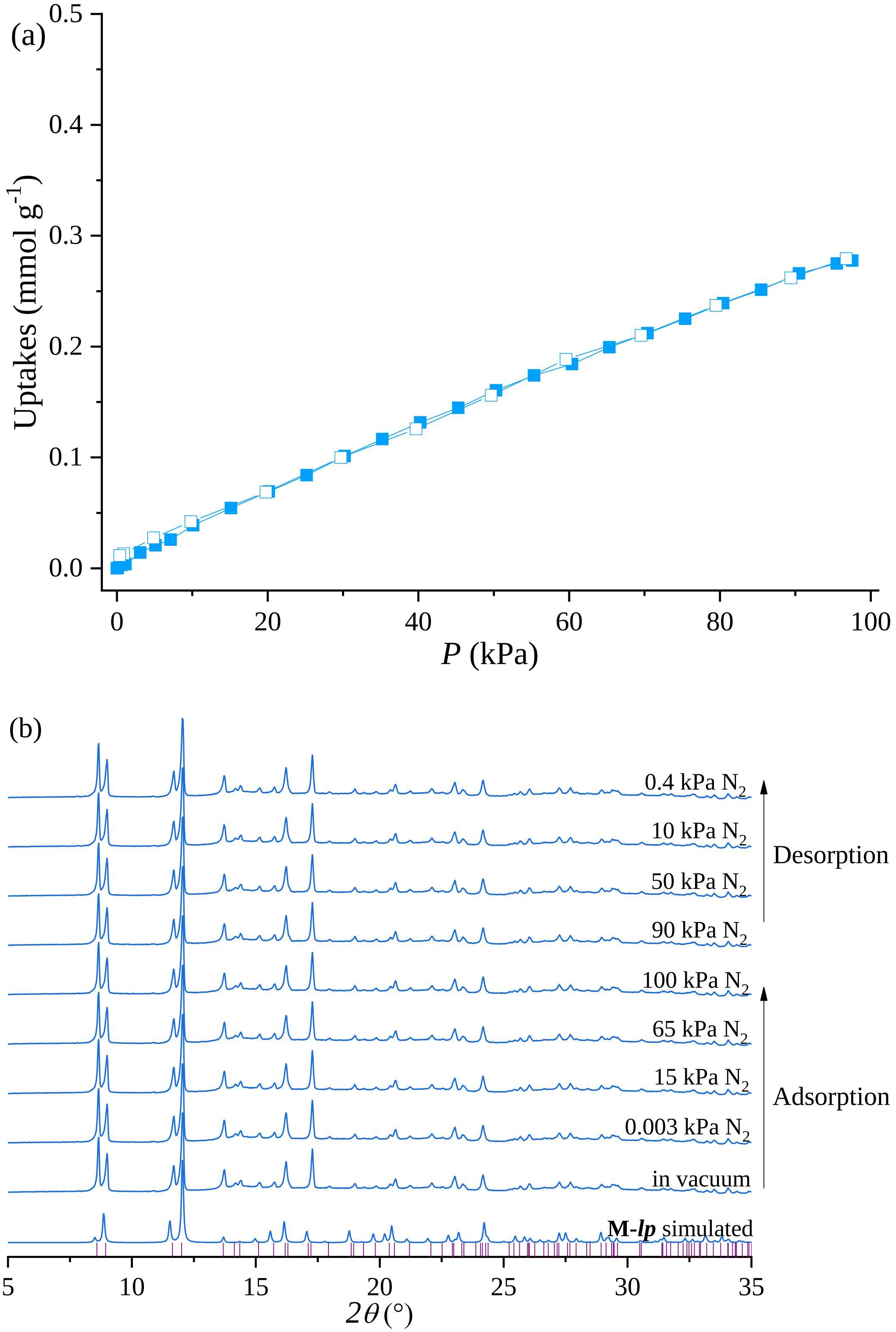


**Figure S40.** *In-situ* sorption-PXRD measurements of **M-*lp*** for $N_2$ at 298 K. (a) Adsorption and desorption isotherms. (b) PXRD patterns.

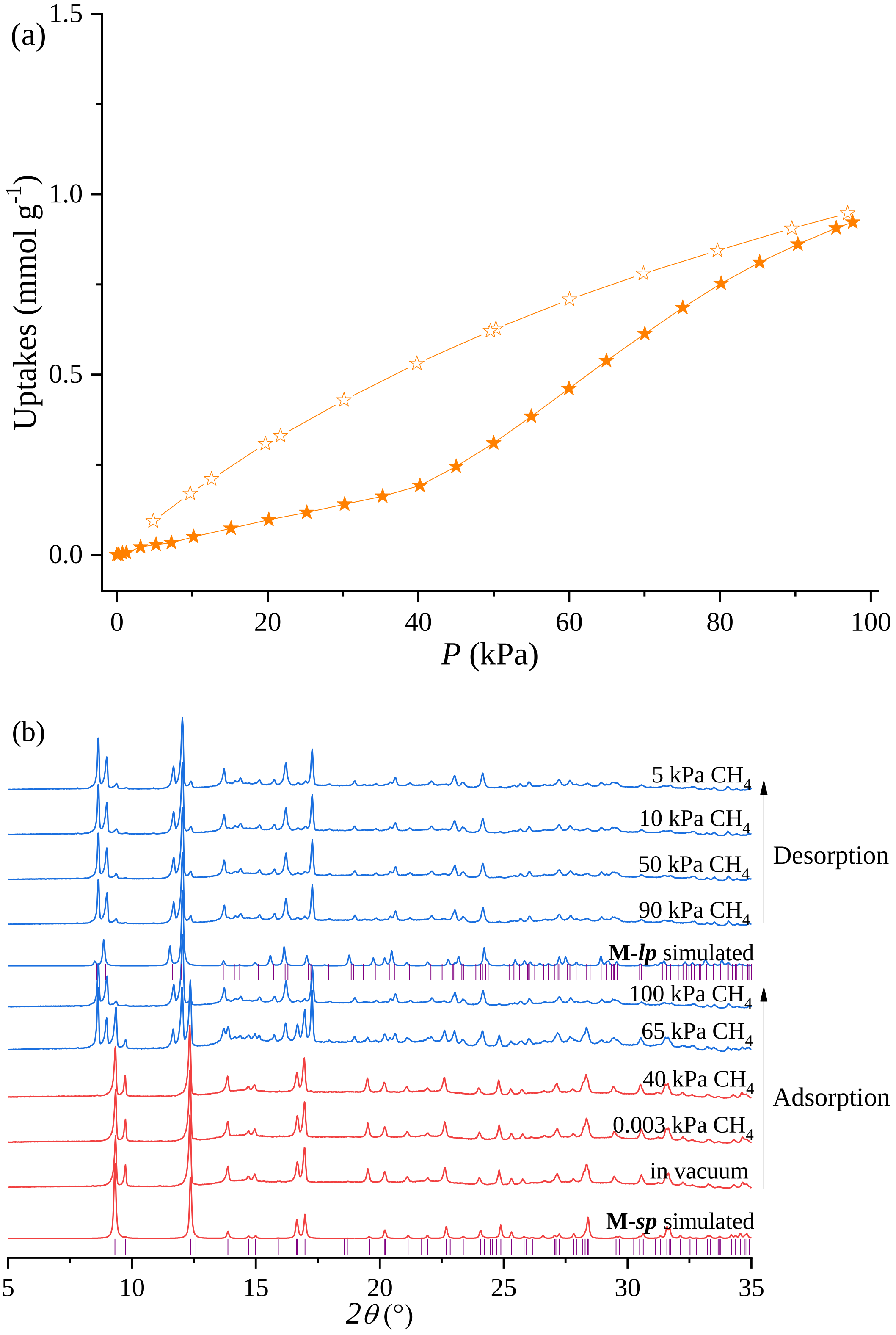


**Figure S41.** *In-situ* sorption-PXRD measurements of **M-*sp*** for $CH_4$ at 298 K. (a) Adsorption and desorption isotherms. (b) PXRD patterns.

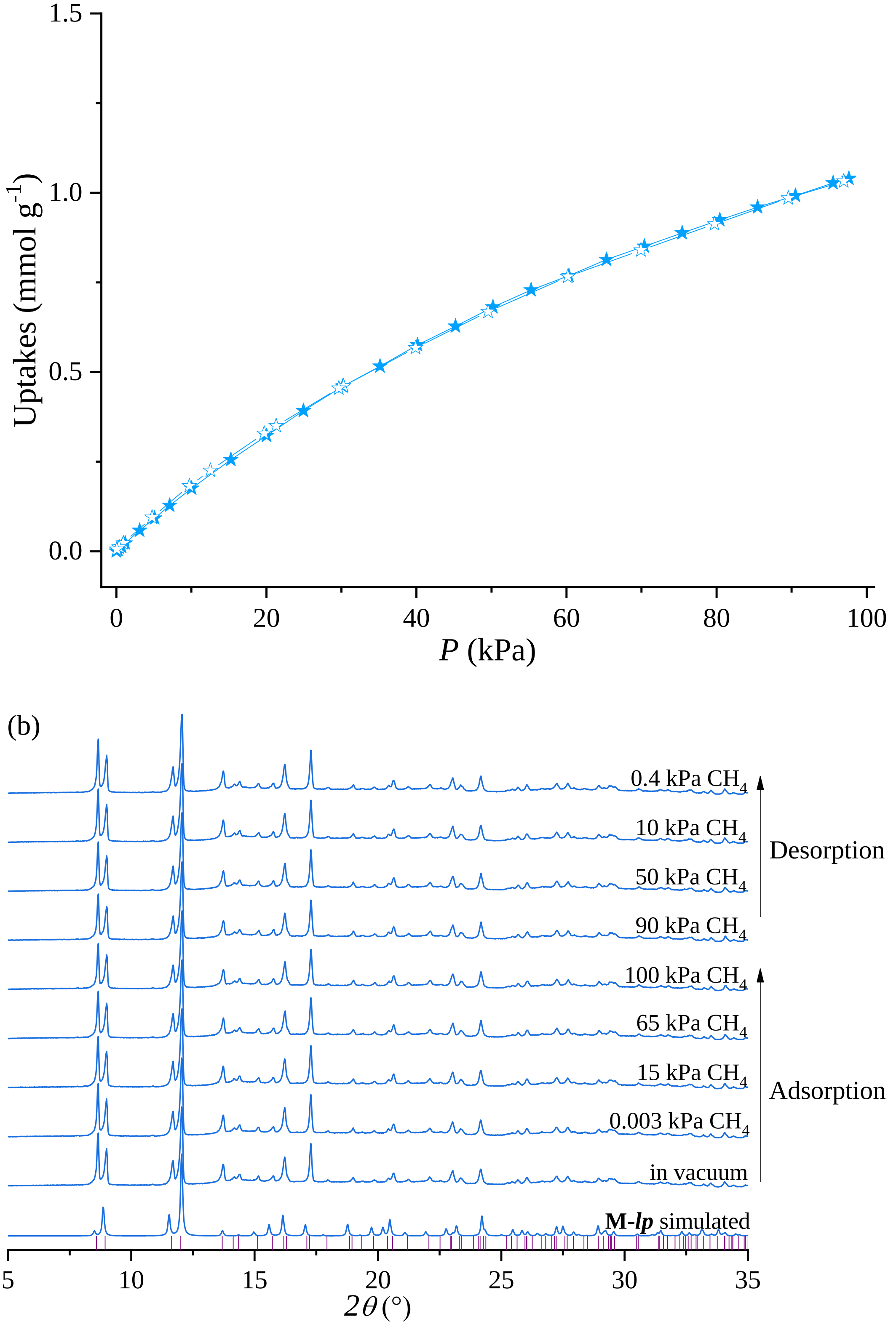


**Figure S42.** *In-situ* sorption-PXRD measurements of **M-*lp*** for $CH_4$ at 298 K. (a) Adsorption and desorption isotherms. (b) PXRD patterns.

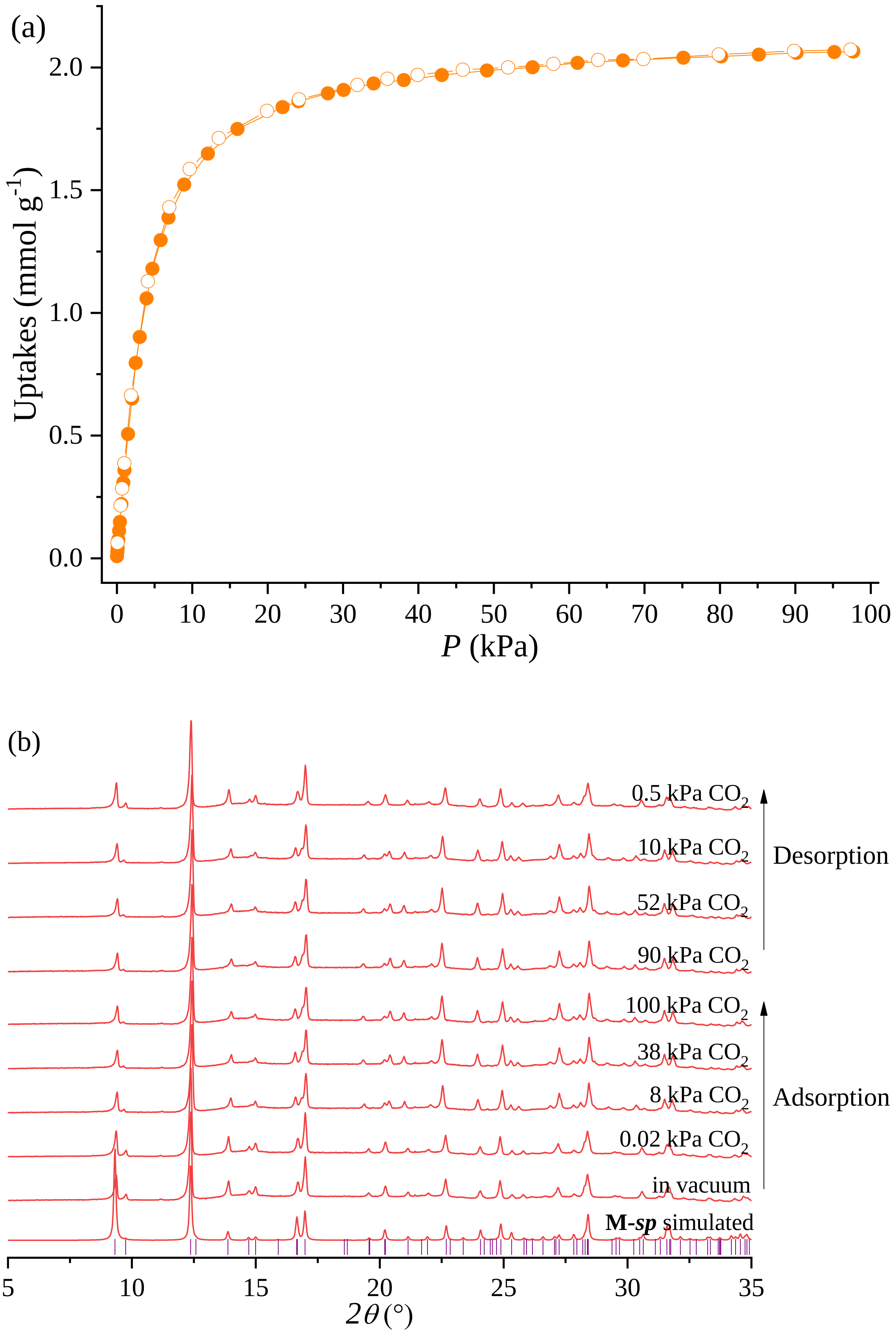


**Figure S43.** *In-situ* sorption-PXRD measurements of **M-*sp*** for $CO_2$ at 298 K. (a) Adsorption and desorption isotherms. (b) PXRD patterns.

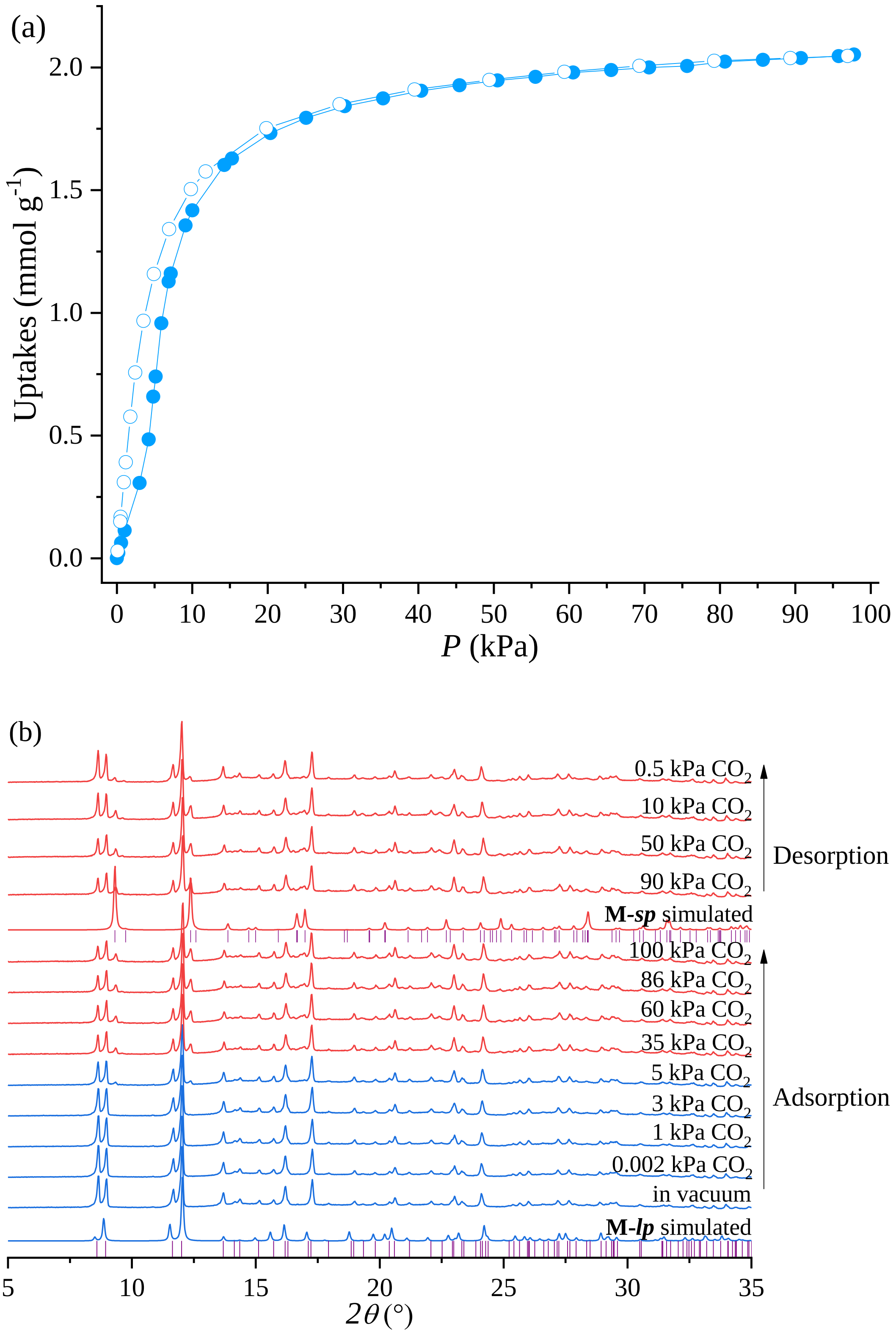


**Figure S44.** *In-situ* sorption-PXRD measurements of **M-*lp*** for $CO_2$ at 298 K. (a) Adsorption and desorption isotherms. (b) PXRD patterns.

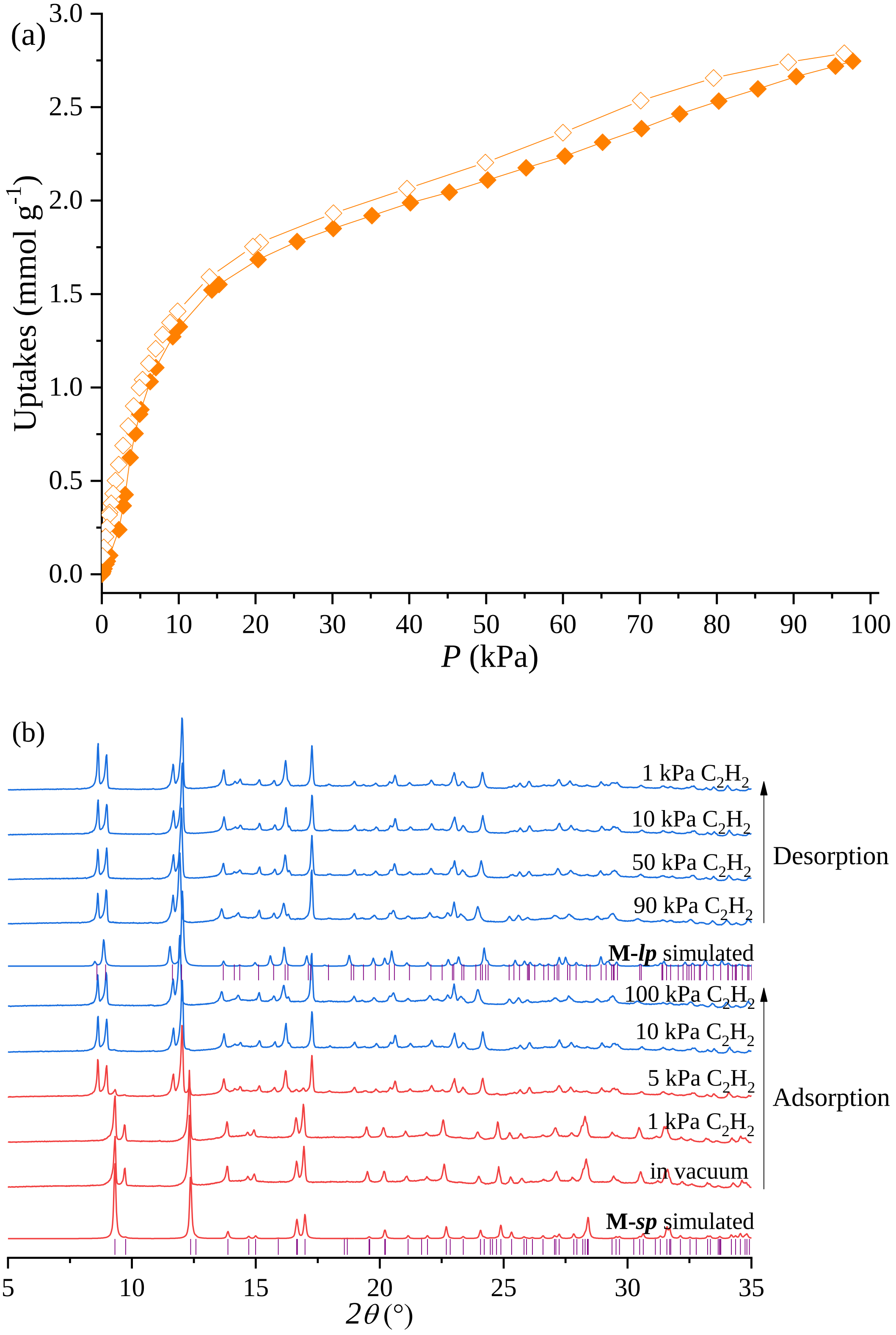


**Figure S45.** *In-situ* sorption-PXRD measurements of **M-*sp*** for $C_2H_2$ at 298 K. (a) Adsorption and desorption isotherms. (b) PXRD patterns.

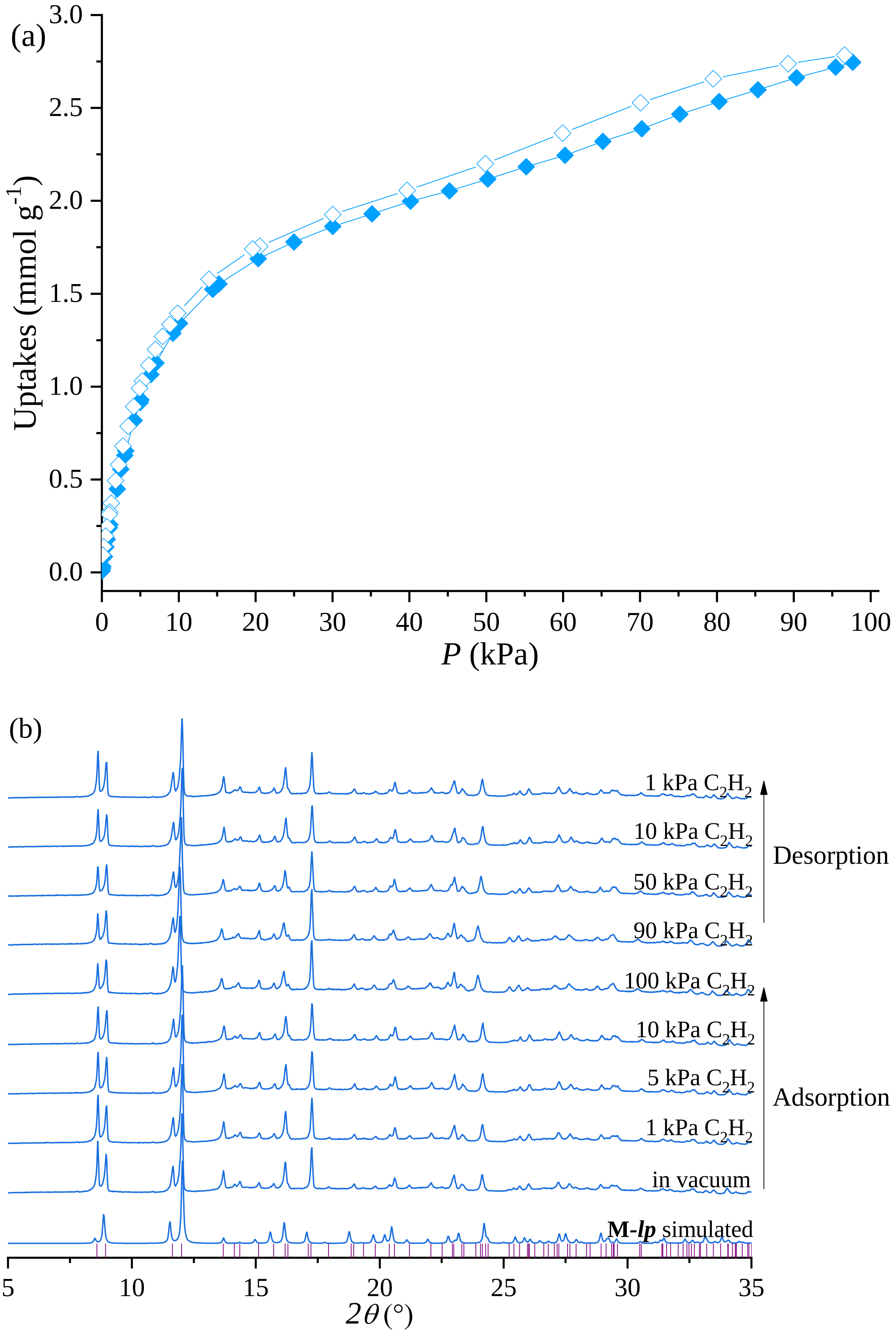


**Figure S46.** *In-situ* sorption-PXRD measurements of **M-*lp*** for $C_2H_2$ at 298 K. (a) Adsorption and desorption isotherms. (b) PXRD patterns.

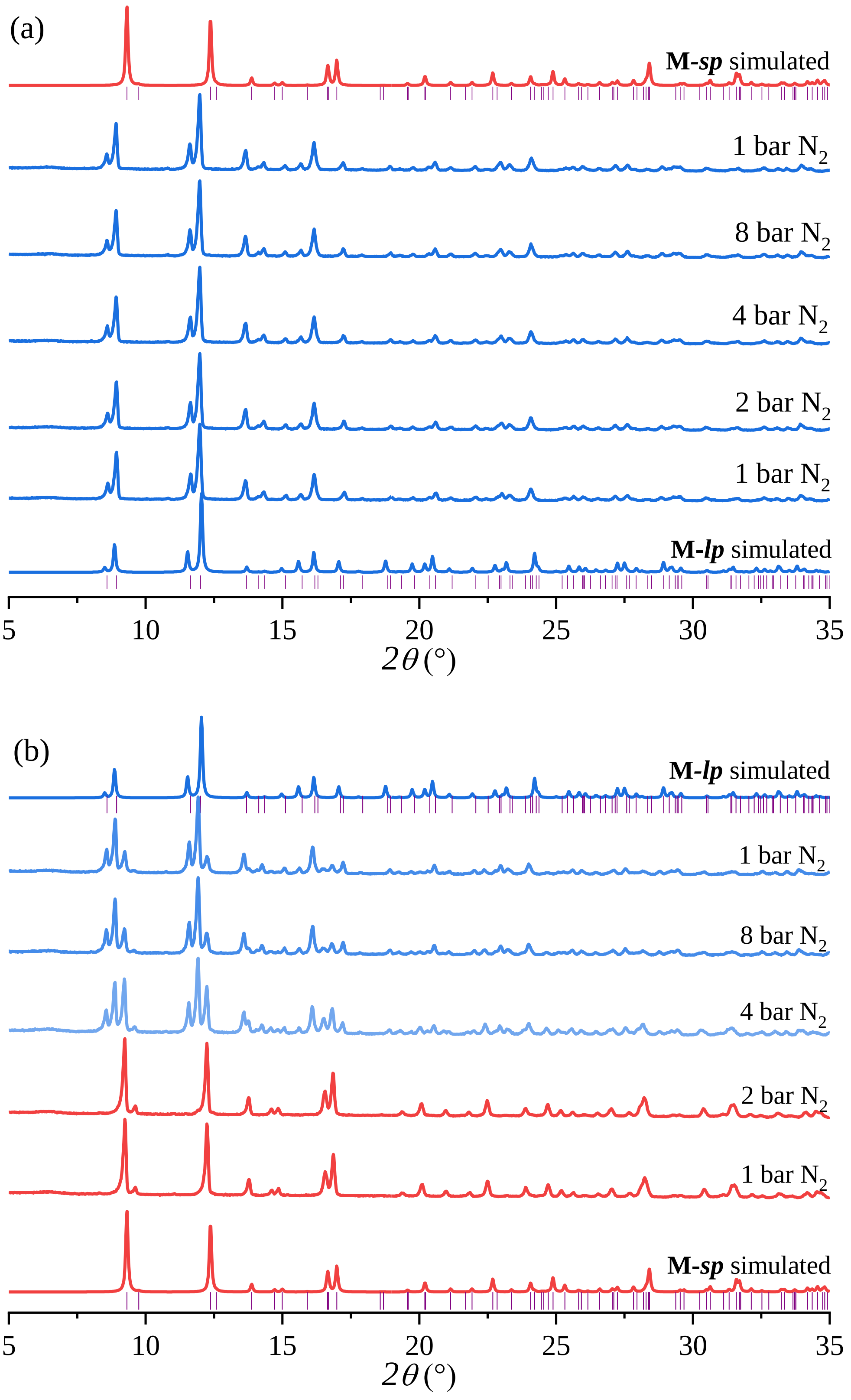


**Figure S47.** PXRD patterns of (a) **M-*lp*** and (b) **M-*sp*** in high-pressure $N_2$ at 298 K.

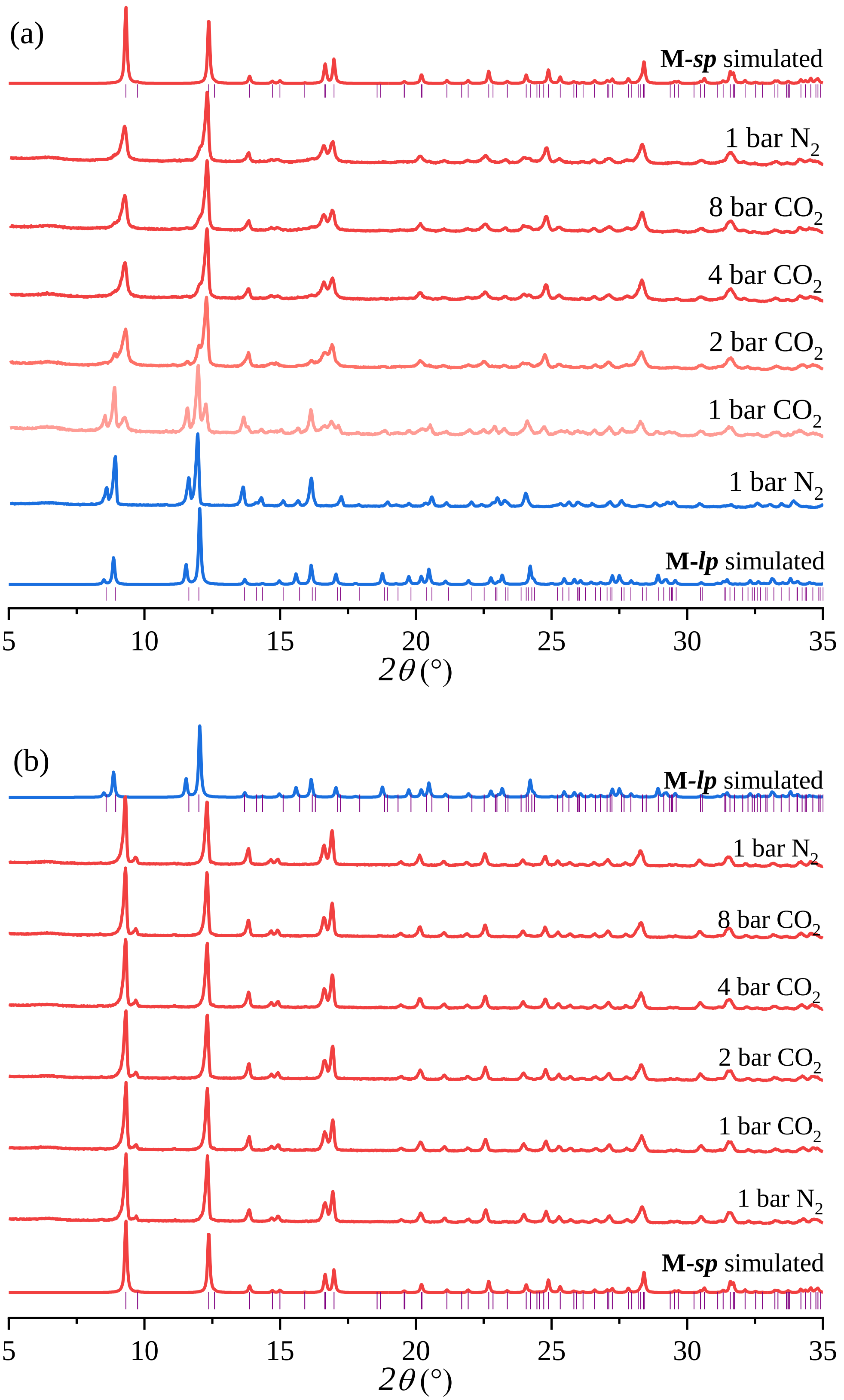


**Figure S48.** PXRD patterns of (a) **M-*lp*** and (b) **M-*sp*** in high-pressure $CO_2$ at 298 K.

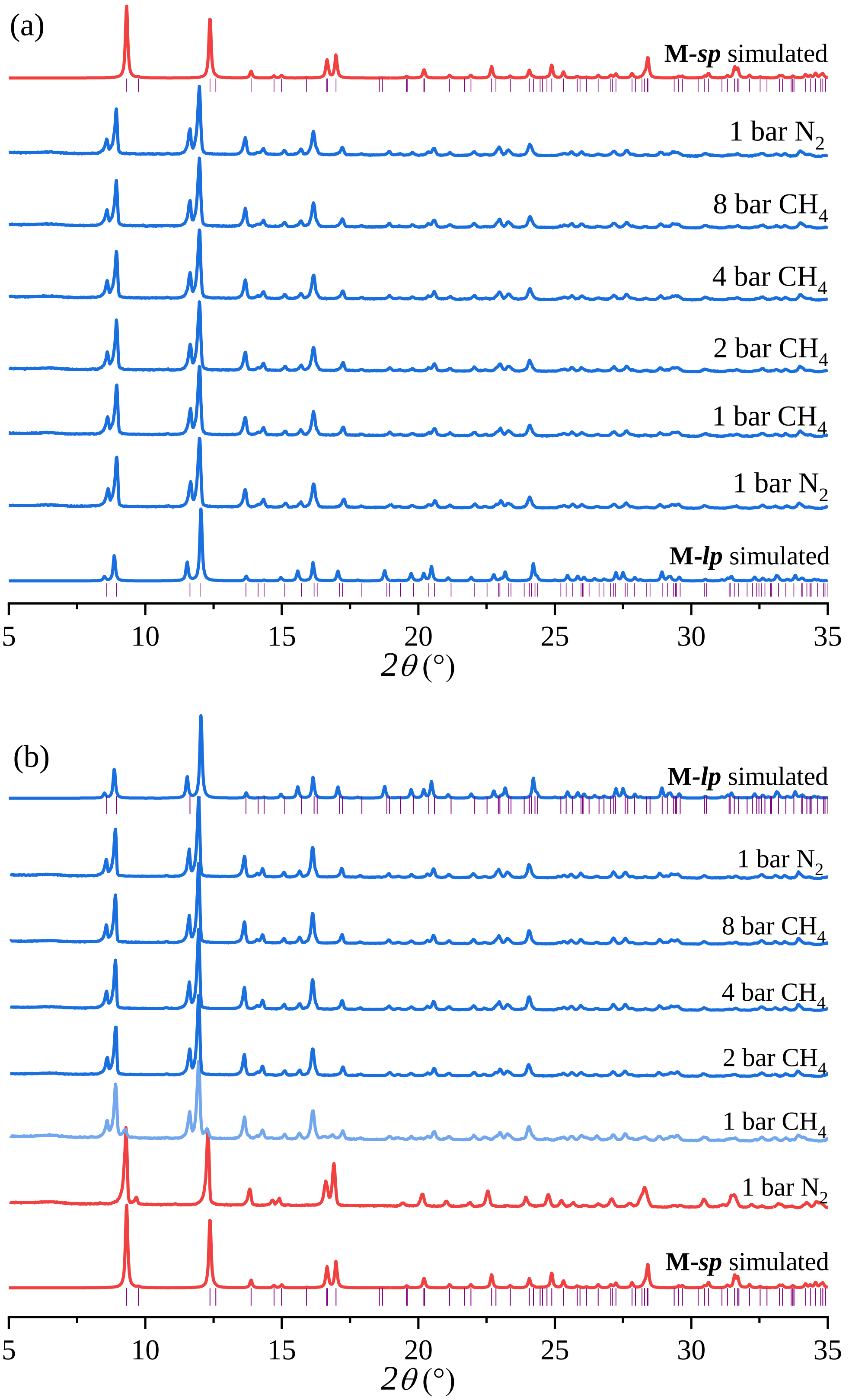


**Figure S49.** PXRD patterns of (a) **M-*lp*** and (b) **M-*sp*** in high-pressure $CH_4$ at 298 K.

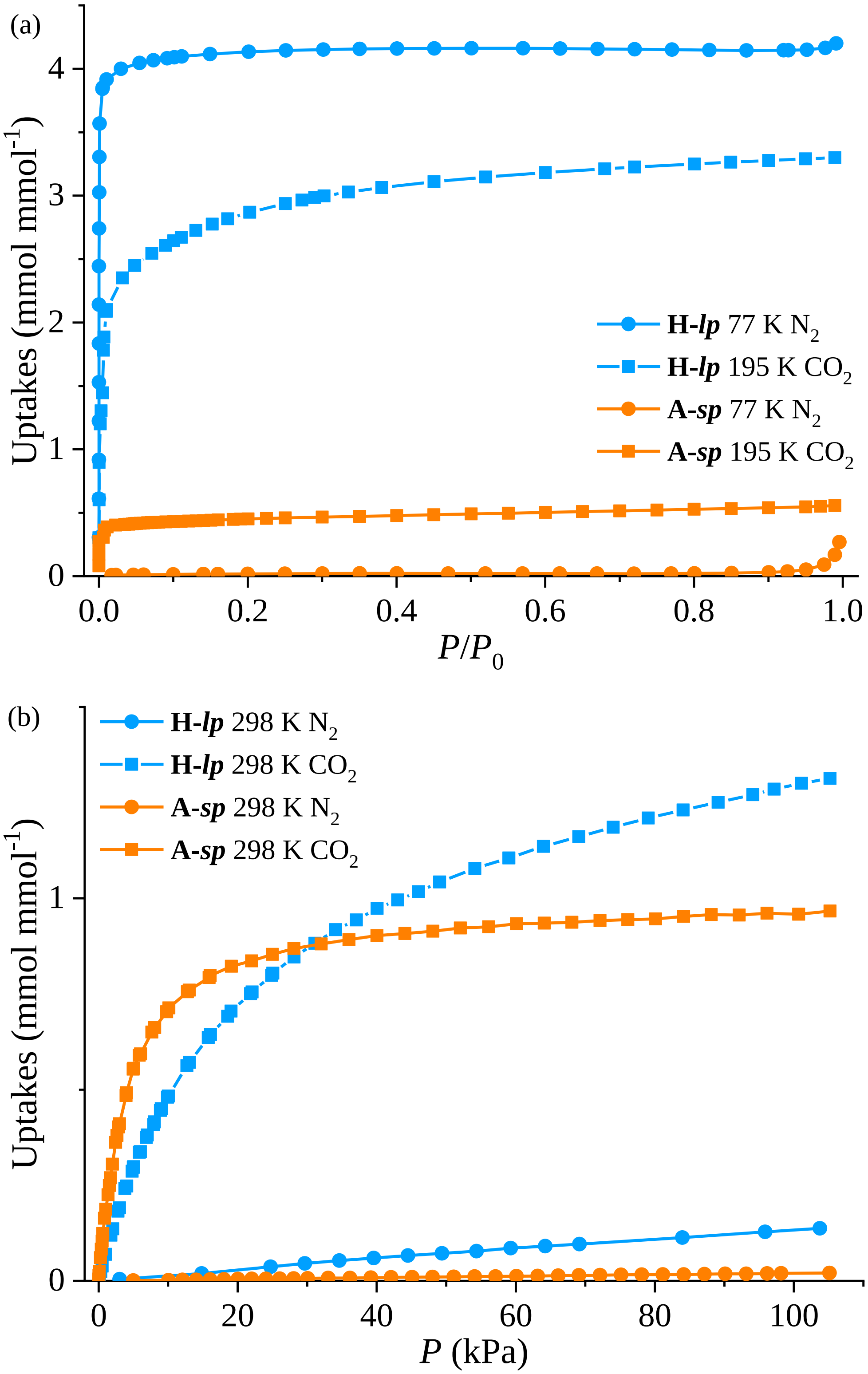


**Figure S50.** (a) Low-temperature and (b) room-temperature $N_2/CO_2/CH_4$ adsorption/desorption isotherms of **H-*lp*** and **A-*sp***.

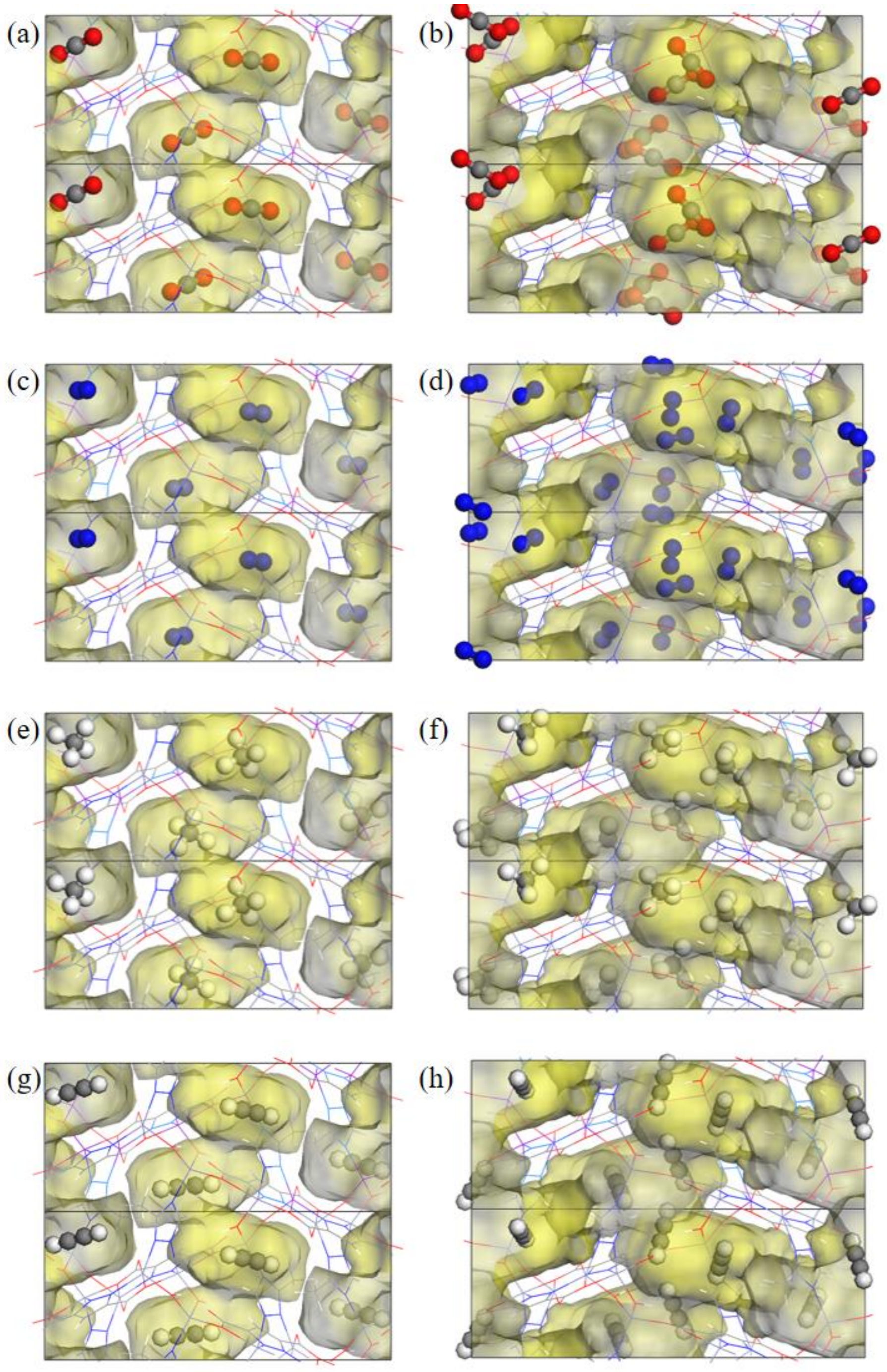


**Figure S51.** Simulated host-guest structures of (a,b) $CO_2$, (c,d) $N_2$, (e,f) $CH_4$, and (g,h) $C_2H_2$-saturated (a,c,e,g) **M-*sp*** and (b,d,f,h) **M-*lp***.

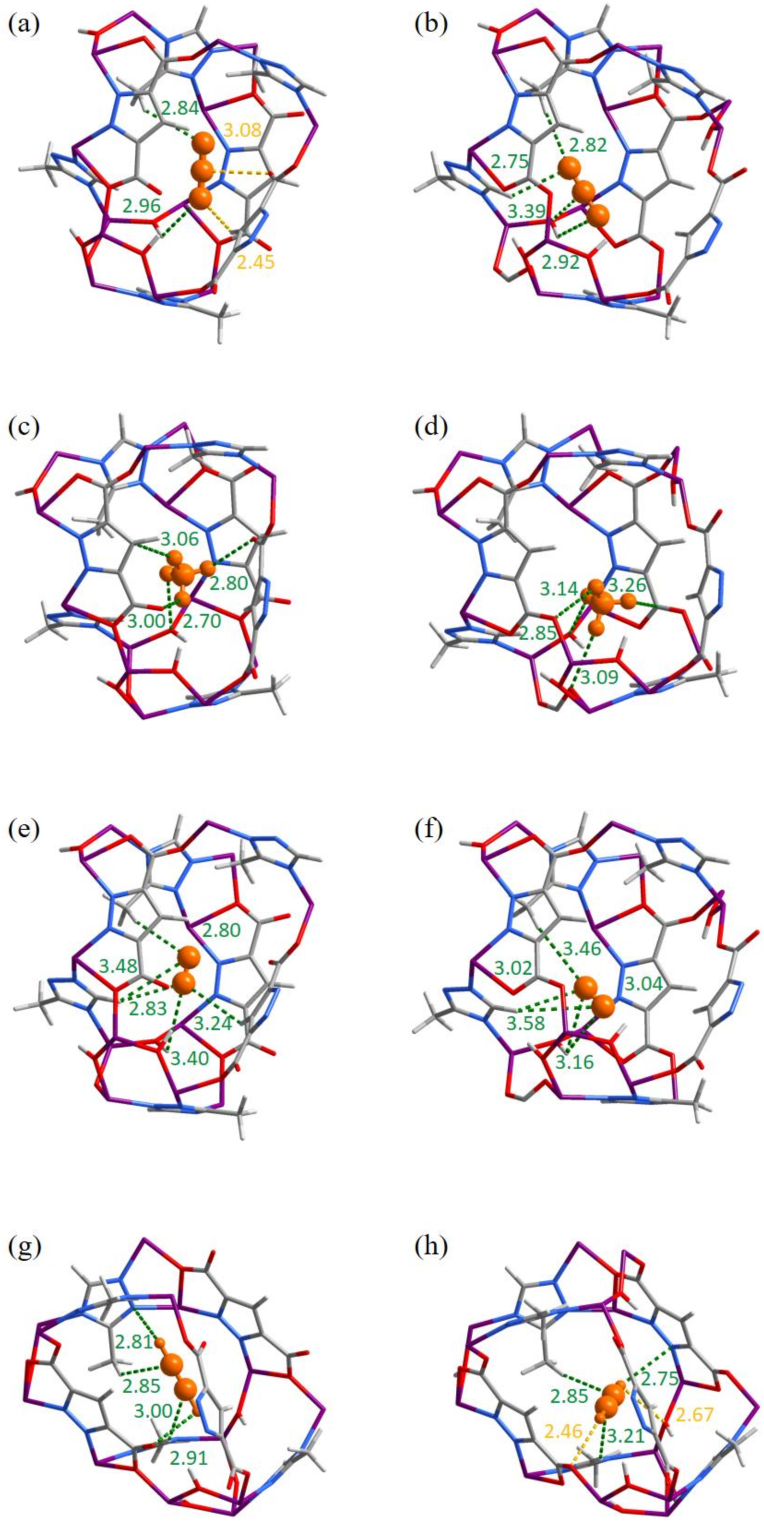


**Figure S52.** Detailed host-guest interactions of (a,b) $CO_2$, (c,d) $CH_4$, (e,f) $N_2$, and (g,h) $C_2H_2$-loaded (a,c,e,g) **M-*sp*** and (b,d,f,h) **M-*lp***.

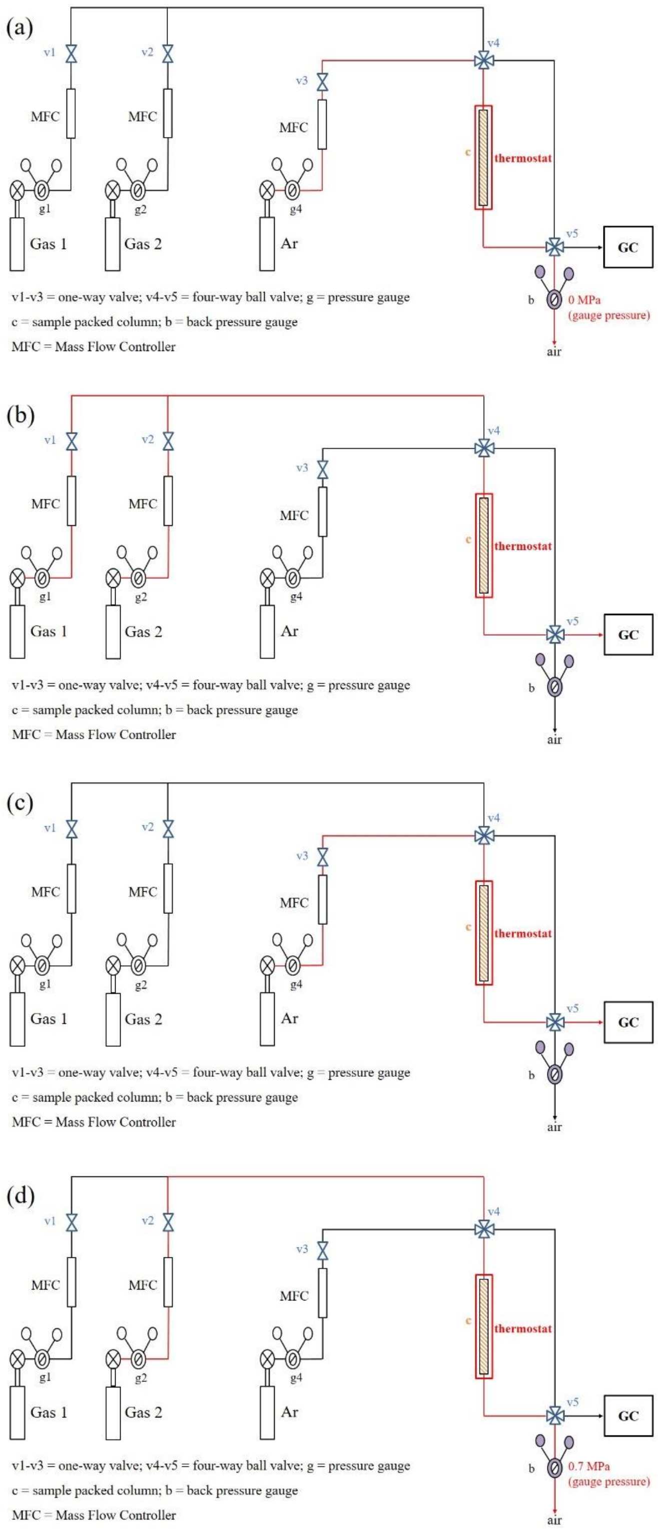


**Figure S53.** The column breakthrough experiment for *in-situ* pore shaping. (a) Activation. (b) Adsorption. (c) Desorption. (d) Pore shaping at high pressure. The red lines represent the gas flowing pathways.

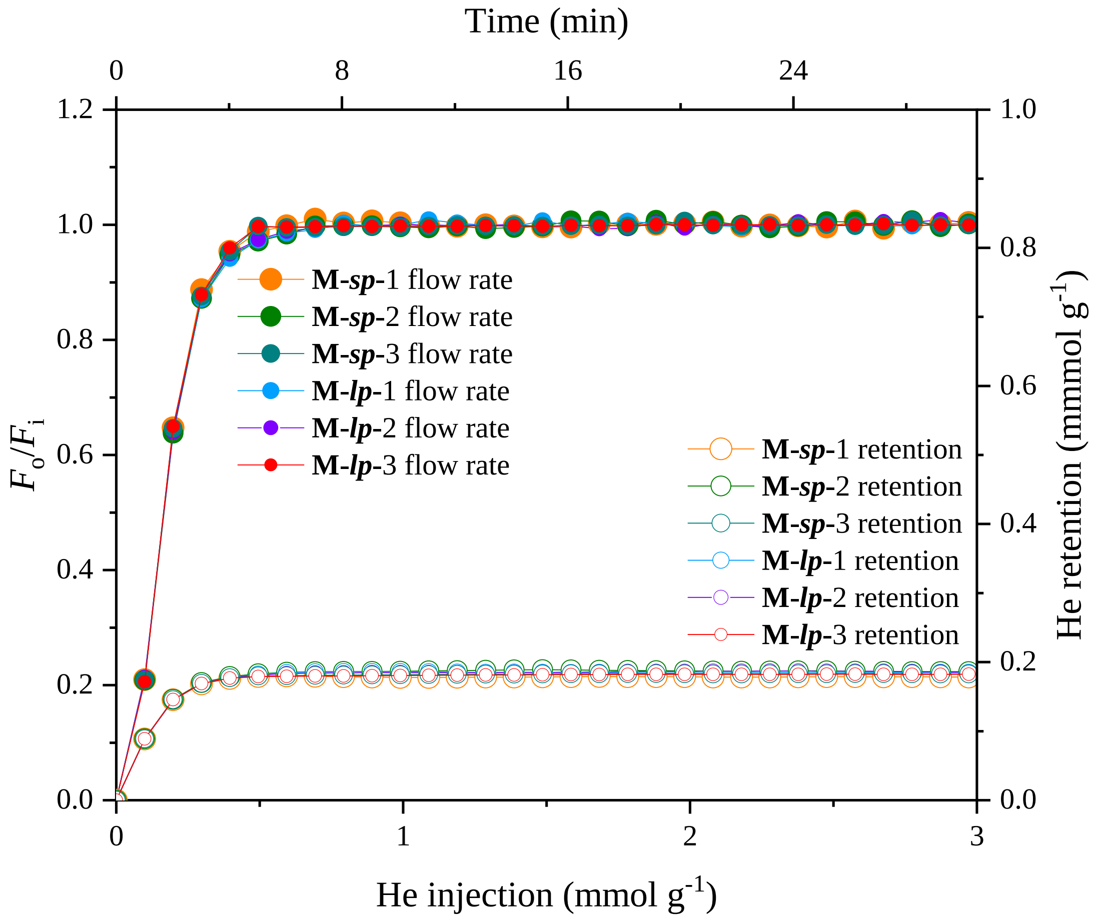


**Figure S54.** Breakthrough for determination of dead-space volume.

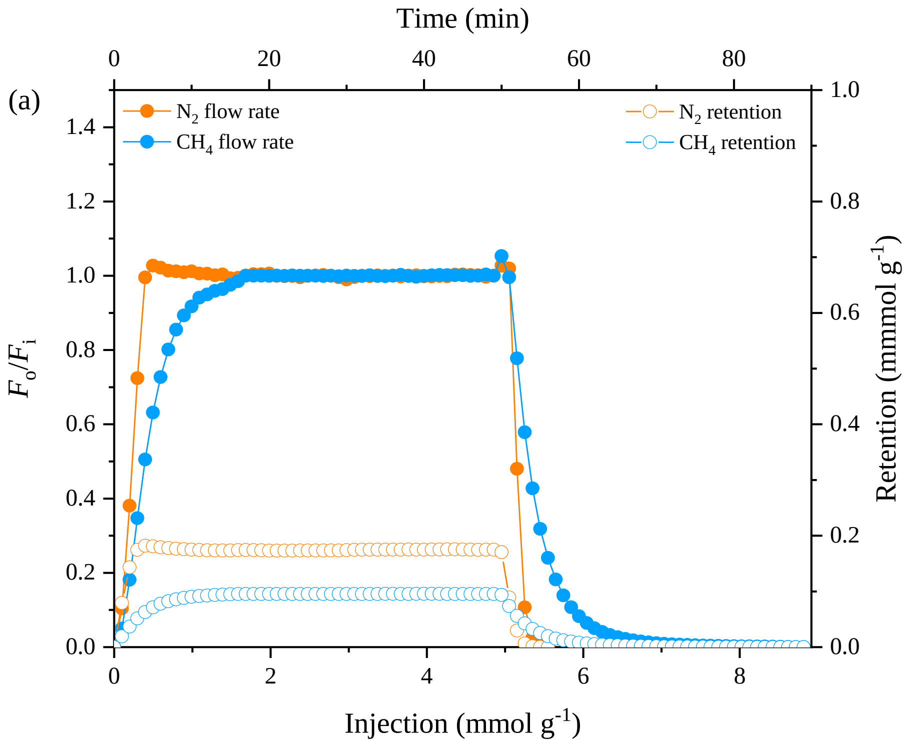


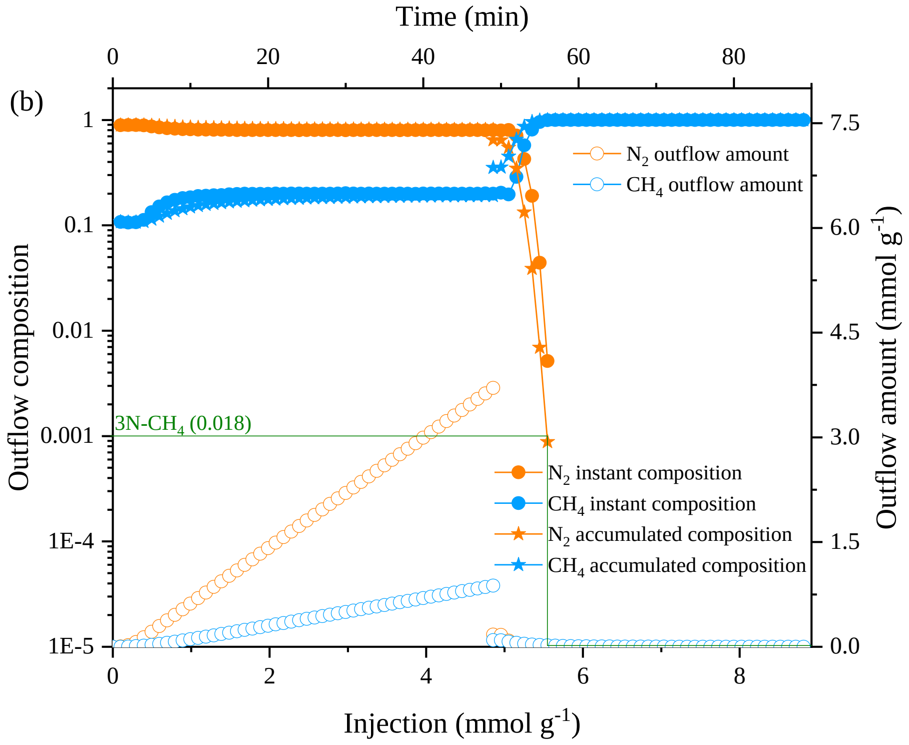


**Figure S55.** Detailed breakthrough curves of **M-*sp*** for a 20:80 $CH_4/N_2$ mixture at 298 K and ambient pressure (1st measurement). (a) Breakthrough and retention curves. (b) Separation performances.

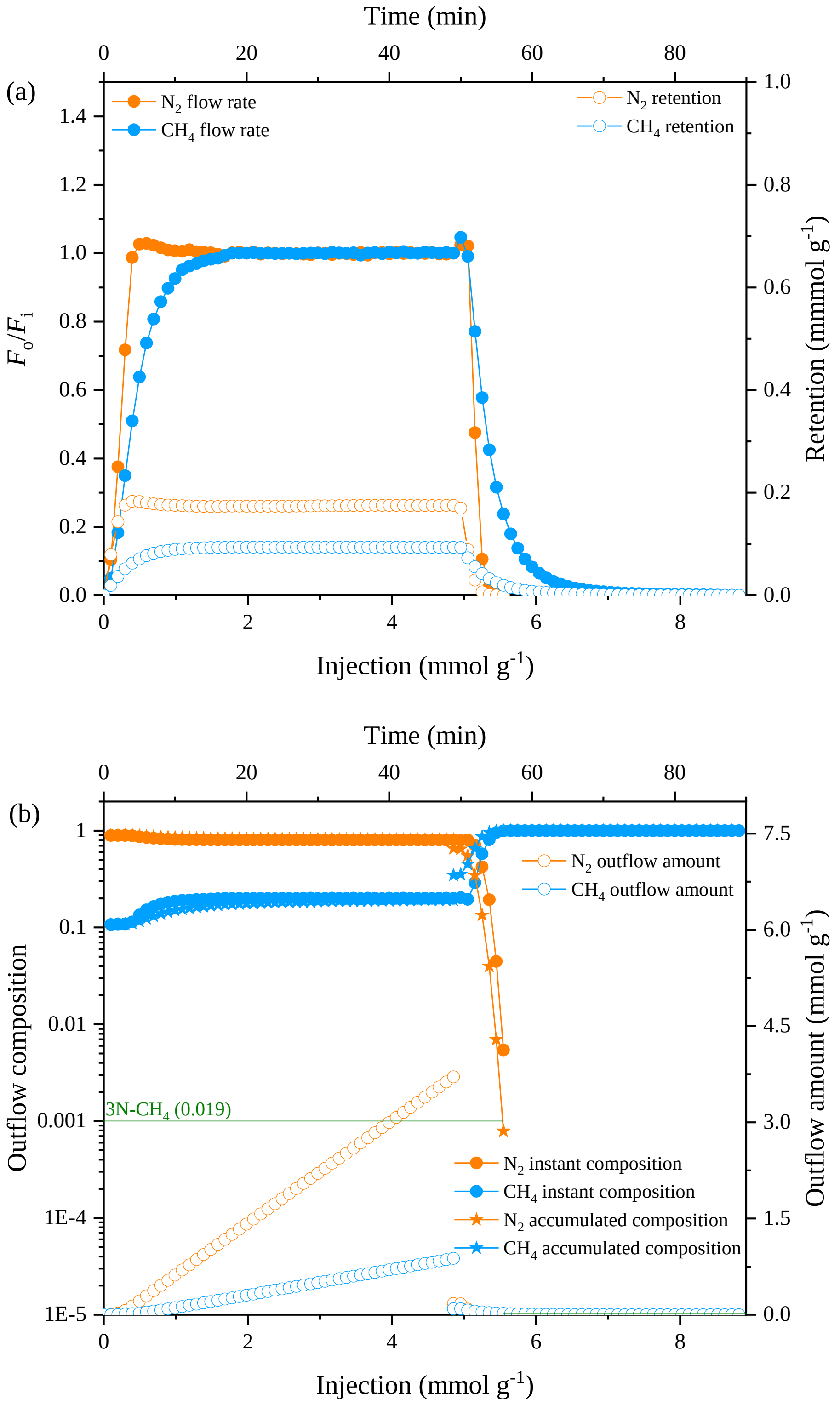


**Figure S56.** Detailed breakthrough curves of **M-*sp*** for a 20:80 $CH_4/N_2$ mixture at 298 K and ambient pressure (2nd measurement). (a) Breakthrough and retention curves. (b) Separation performances.

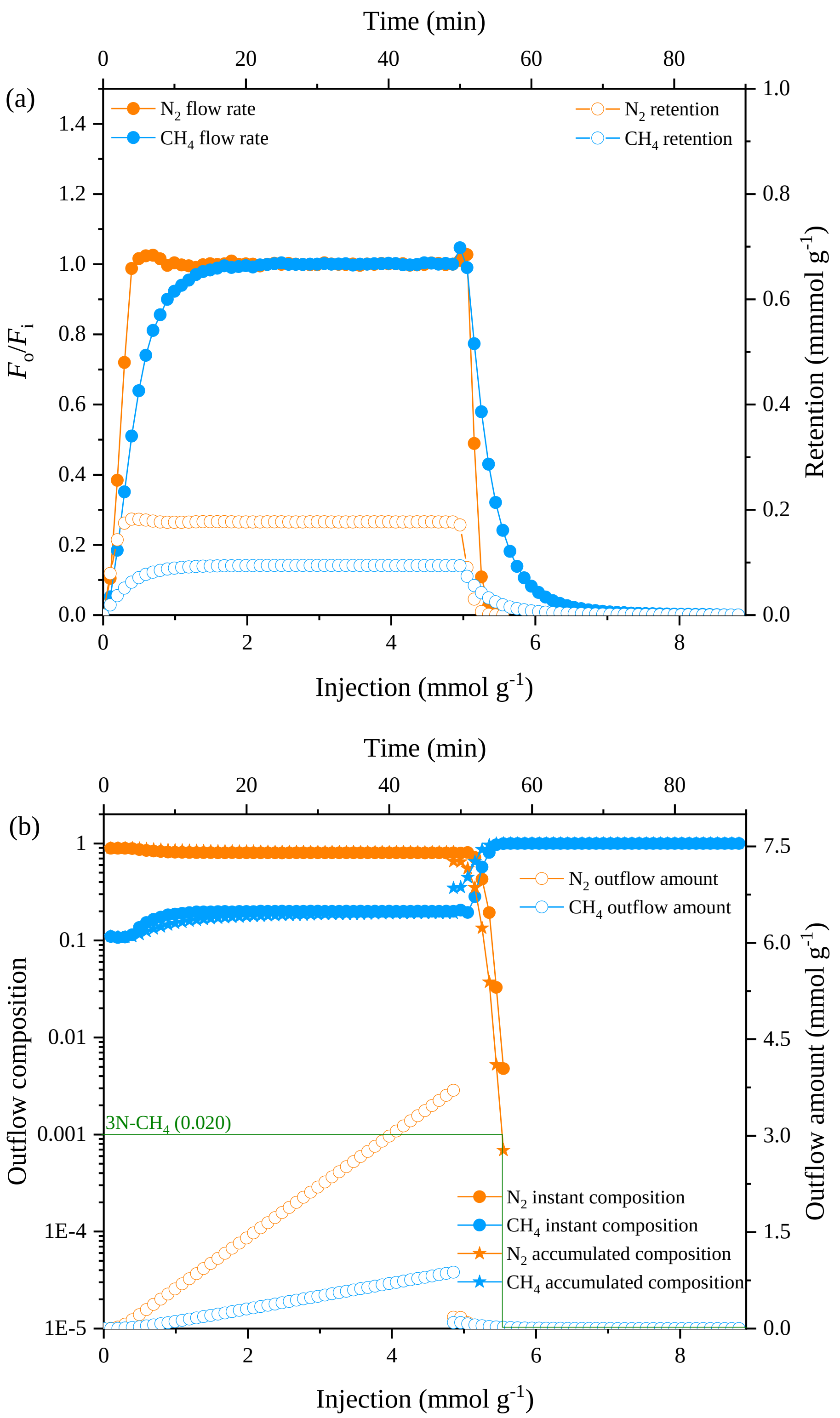


**Figure S57.** Detailed breakthrough curves of **M-*sp*** for a 20:80 $CH_4/N_2$ mixture at 298 K and ambient pressure (3rd measurement). (a) Breakthrough and retention curves. (b) Separation performances.

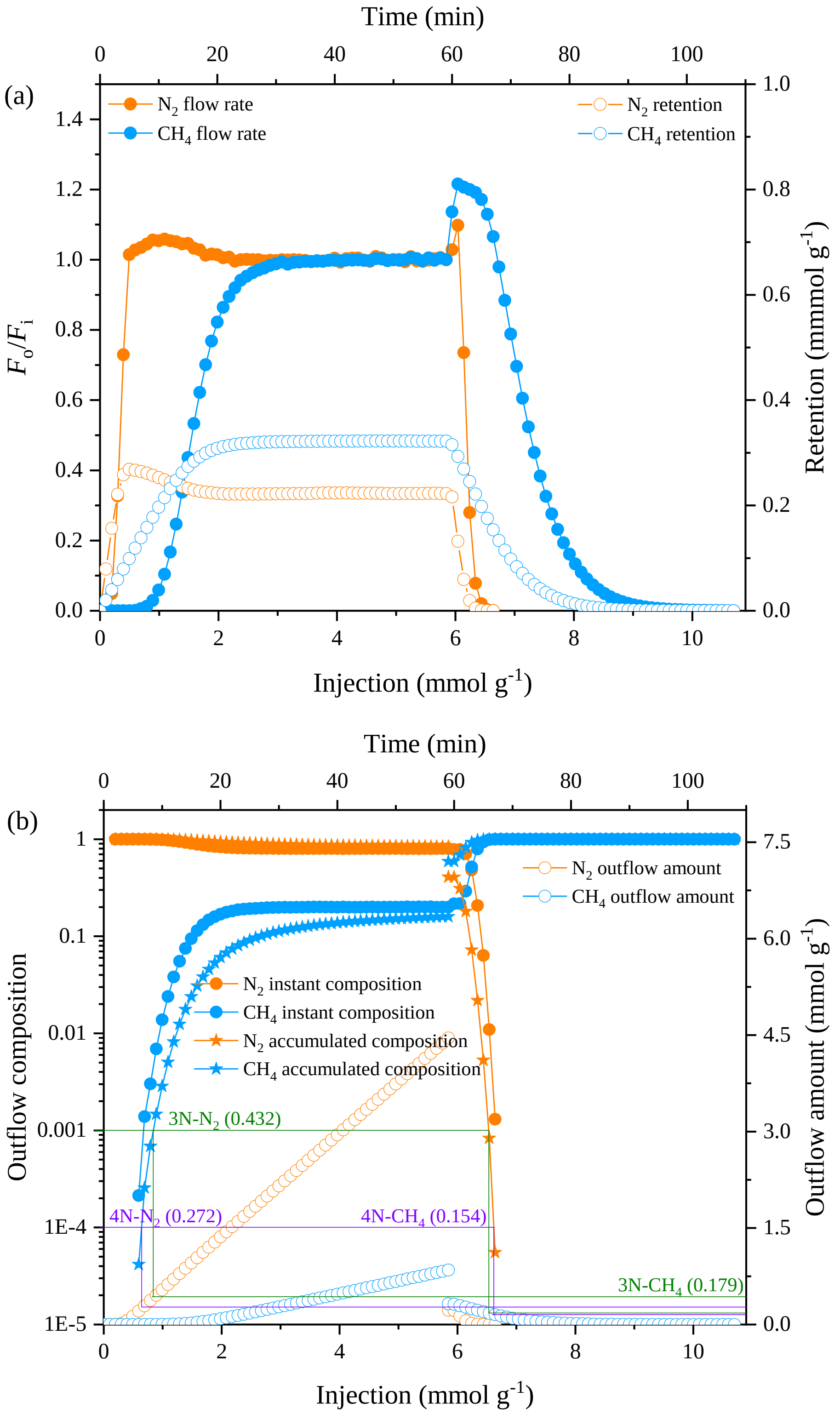


**Figure S58.** Detailed breakthrough curves of **M-*lp*** for a 20:80 $CH_4/N_2$ mixture at 298 K and ambient pressure (1st measurement). (a) Breakthrough and retention curves. (b) Separation performances.

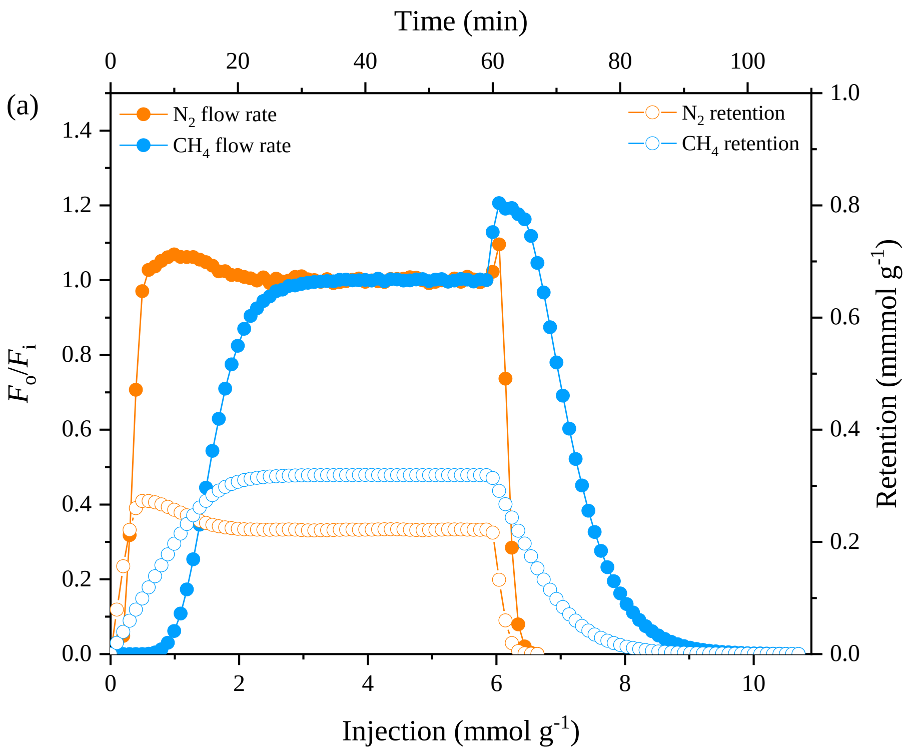


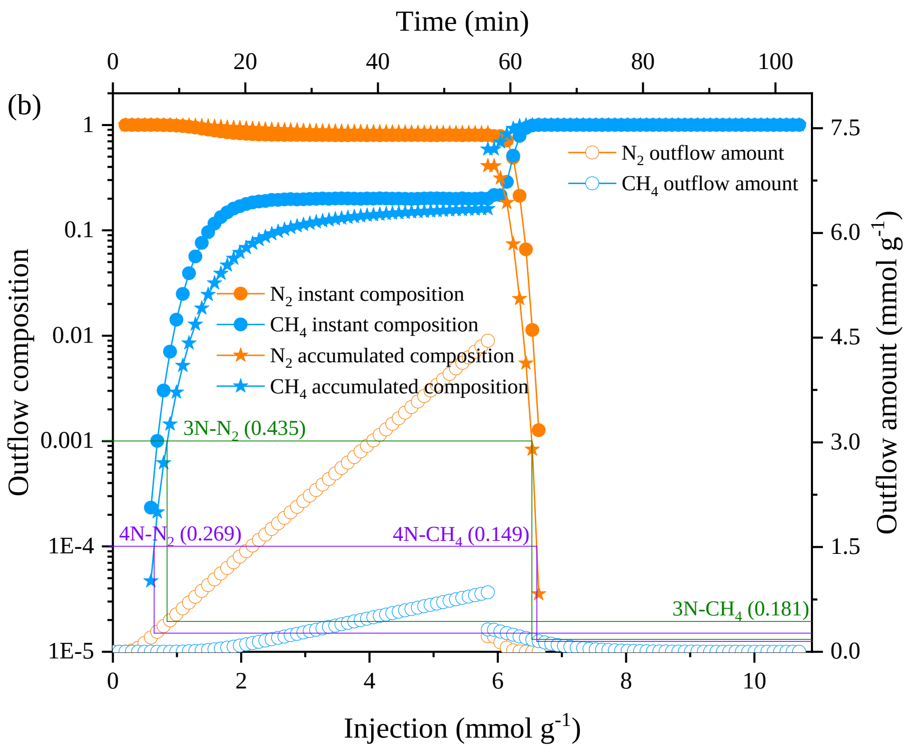


**Figure S59.** Detailed breakthrough curves of **M-*lp*** for a 20:80 $CH_4/N_2$ mixture at 298 K and ambient pressure (2nd measurement). (a) Breakthrough and retention curves. (b) Separation performances.

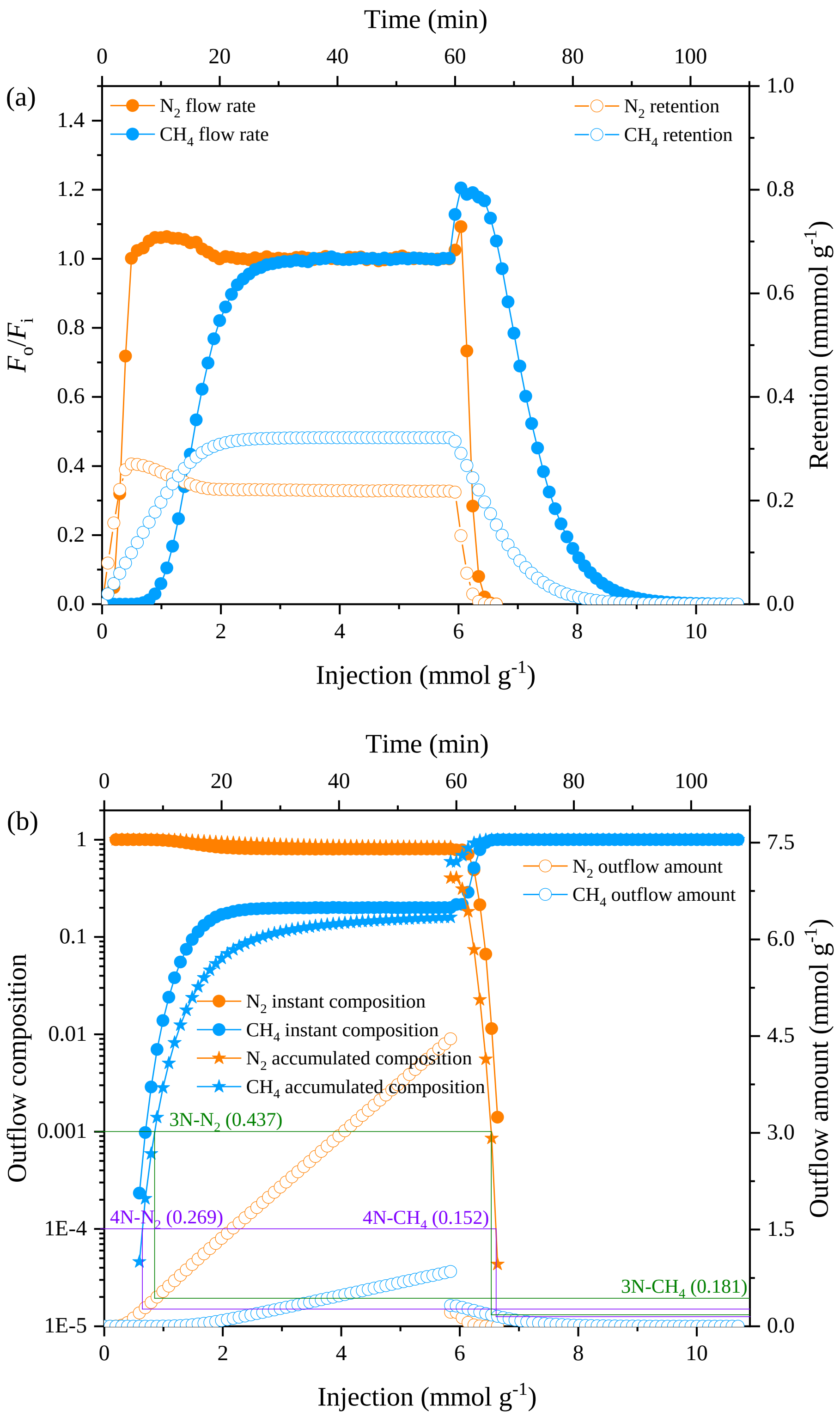


**Figure S60.** Detailed breakthrough curves of **M-*lp*** for a 20:80 $CH_4/N_2$ mixture at 298 K and ambient pressure (3rd measurement). (a) Breakthrough and retention curves. (b) Separation performances.

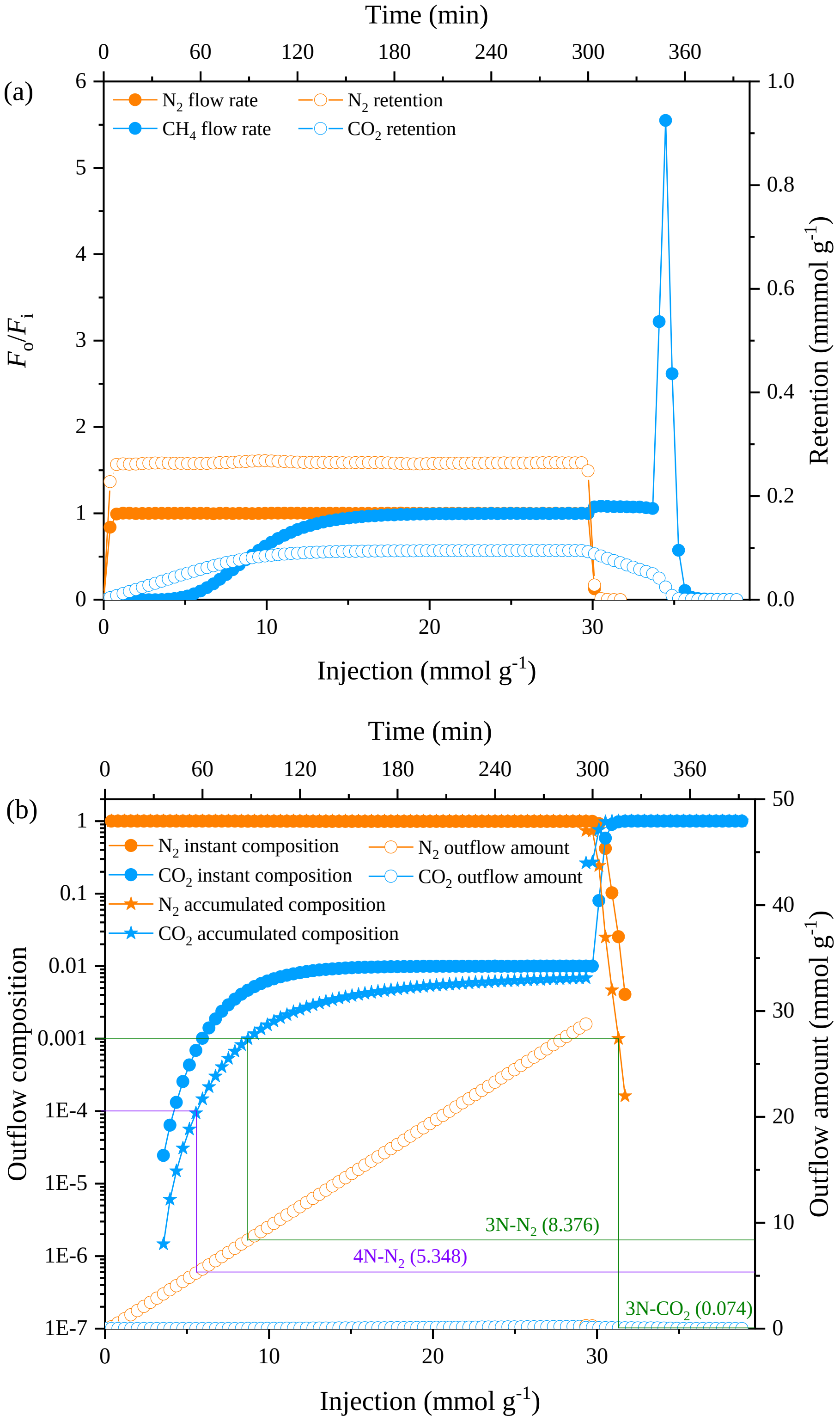


**Figure S61.** Detailed breakthrough curves of **M-*lp*** for a 1:99 $CO_2/N_2$ mixture at 298 K and ambient pressure (1st measurement). (a) Breakthrough and retention curves. (b) Separation performances.

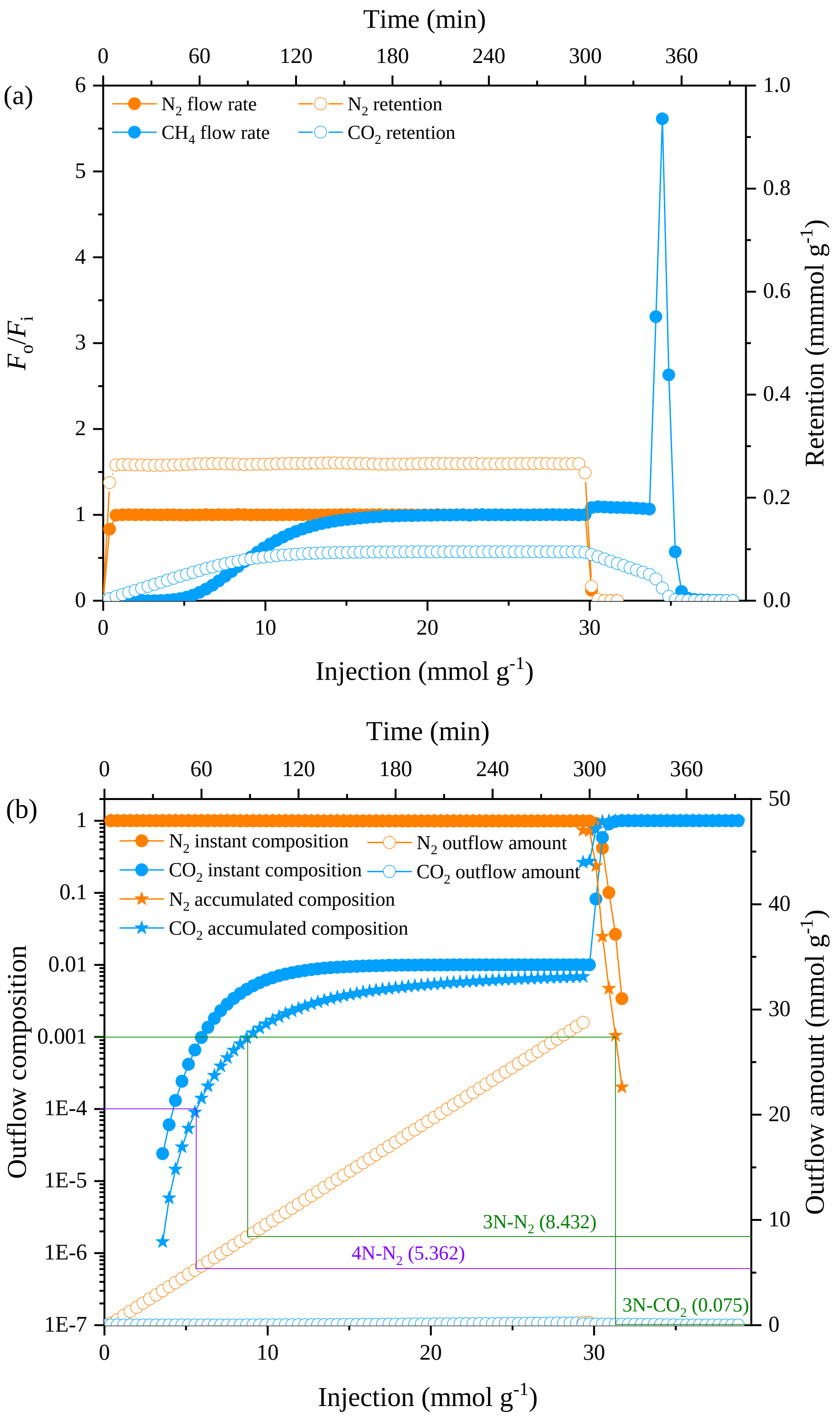


**Figure S62.** Detailed breakthrough curves of **M-*lp*** for a 1:99 $CO_2/N_2$ mixture at 298 K and ambient pressure (2nd measurement). (a) Breakthrough and retention curves. (b) Separation performances.

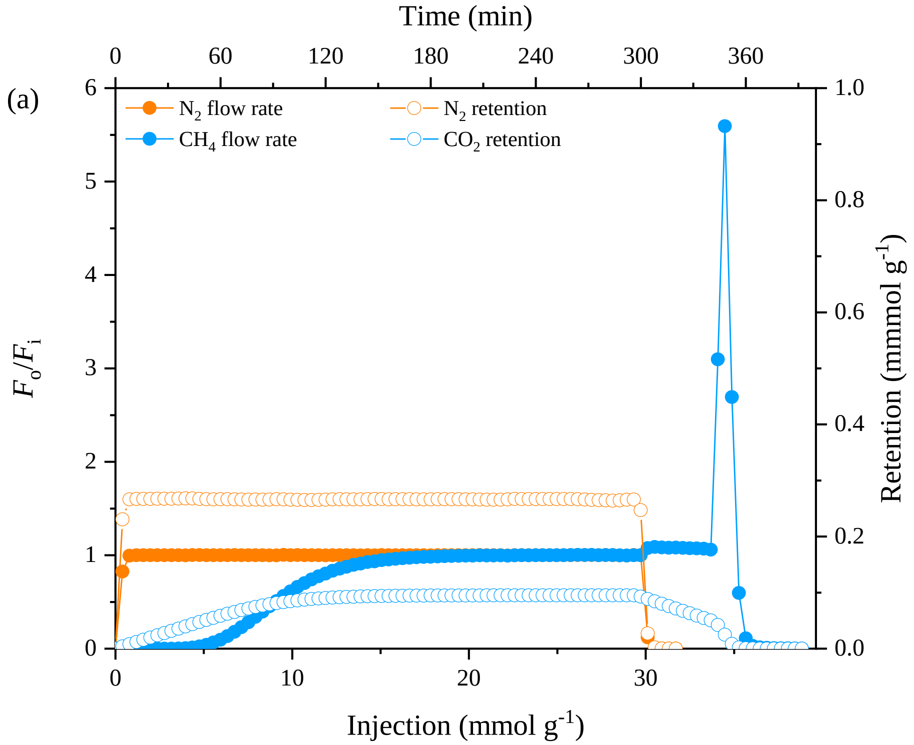


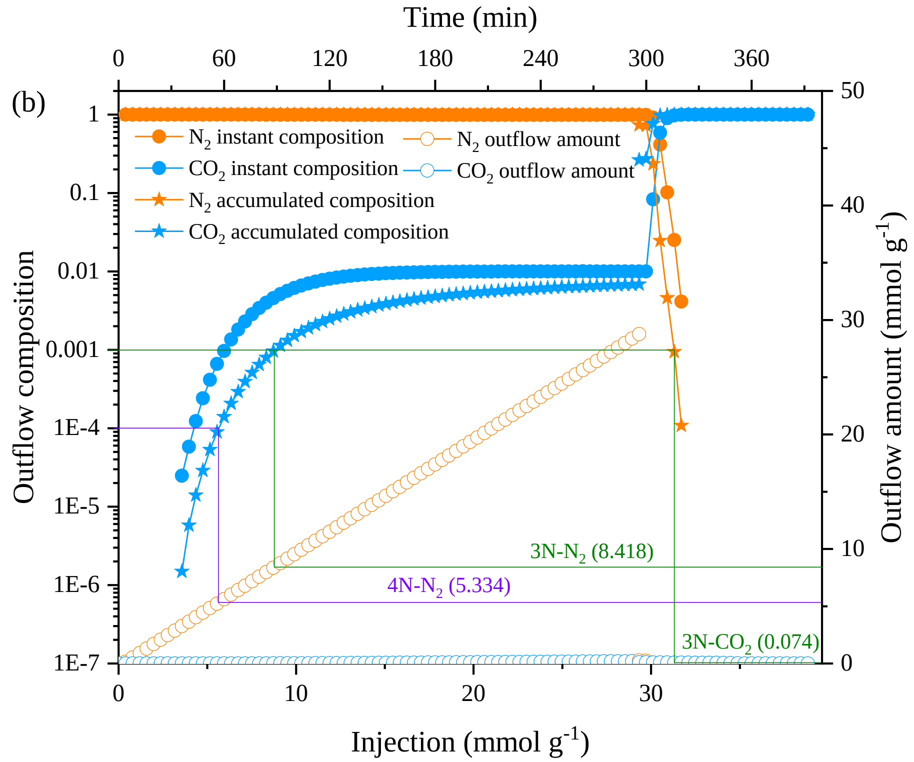


**Figure S63.** Detailed breakthrough curves of **M-*lp*** for a 1:99 $CO_2/N_2$ mixture at 298 K and ambient pressure (3rd measurement). (a) Breakthrough and retention curves. (b) Separation performances.

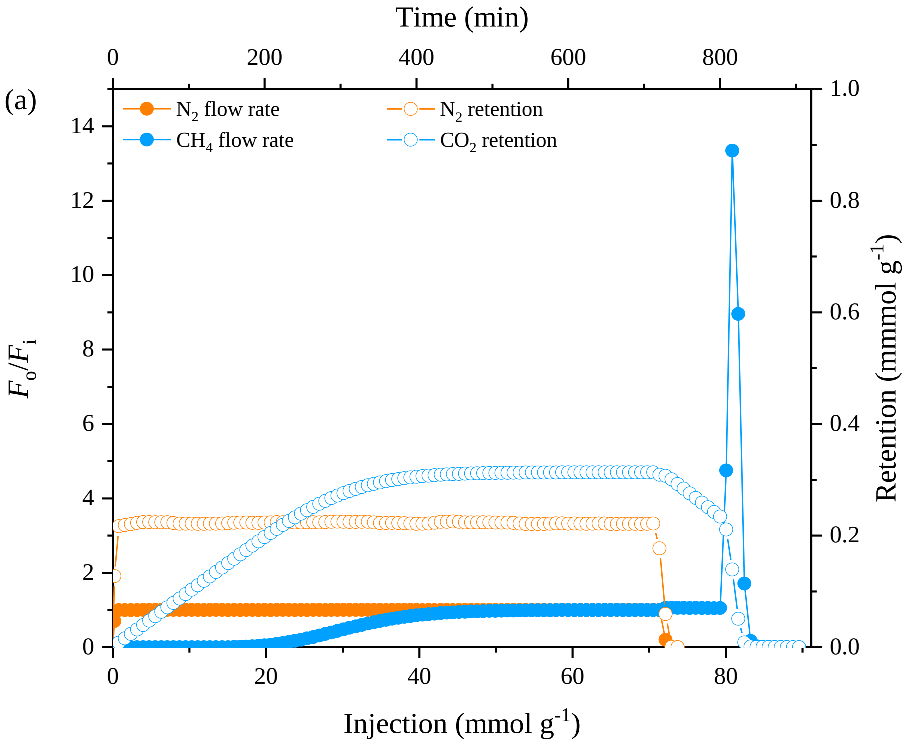


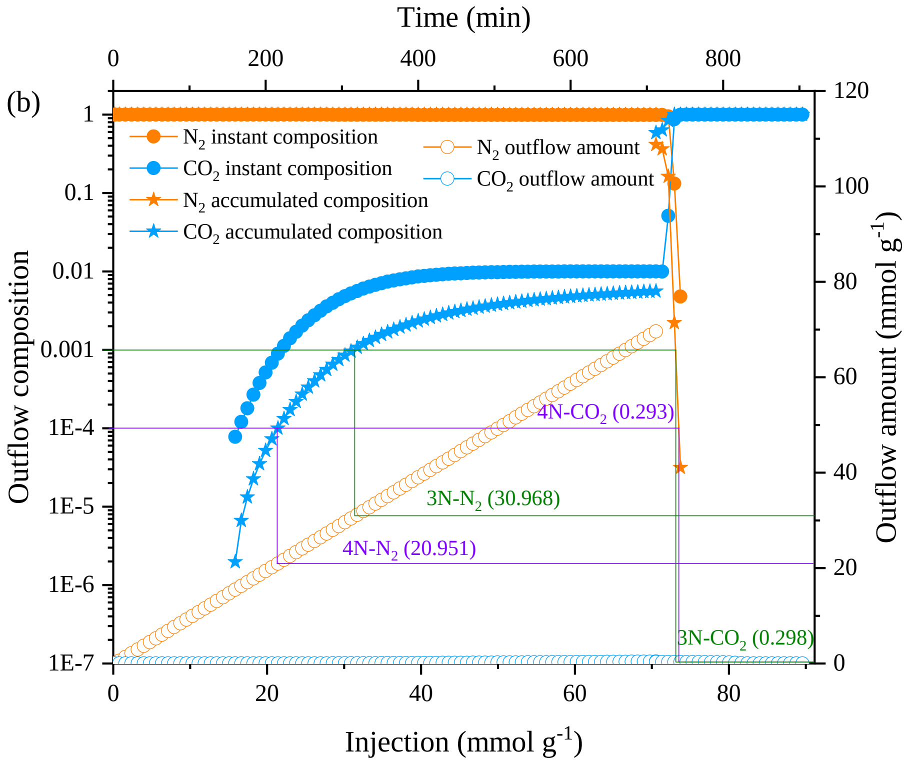


**Figure S64.** Detailed breakthrough curves of **M-*sp*** for a 1:99 $CO_2/N_2$ mixture at 298 K and ambient pressure (1st measurement). (a) Breakthrough and retention curves. (b) Separation performances.

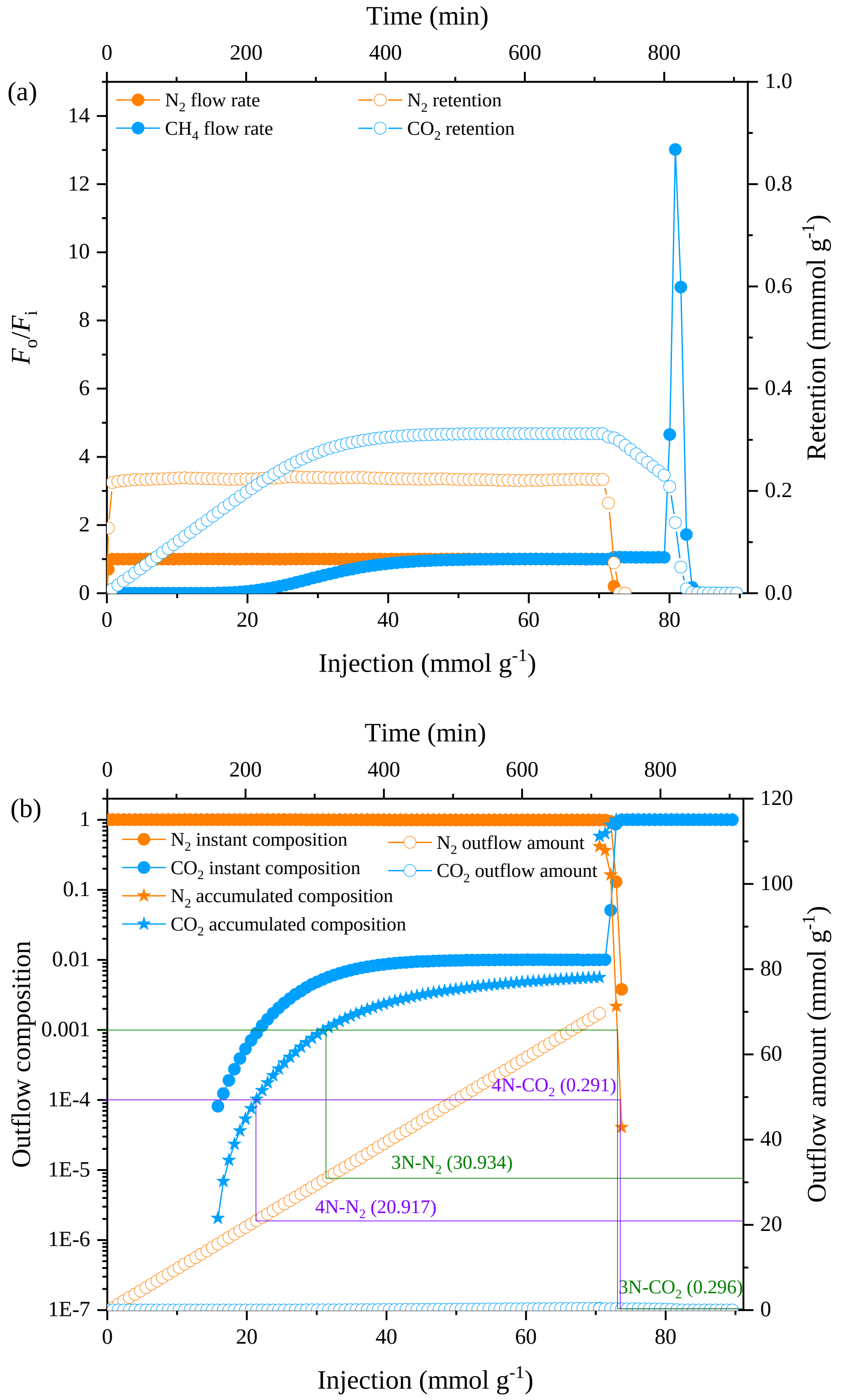


**Figure S65.** Detailed breakthrough curves of **M-*sp*** for a 1:99 $CO_2/N_2$ mixture at 298 K and ambient pressure (2nd measurement). (a) Breakthrough and retention curves. (b) Separation performances.

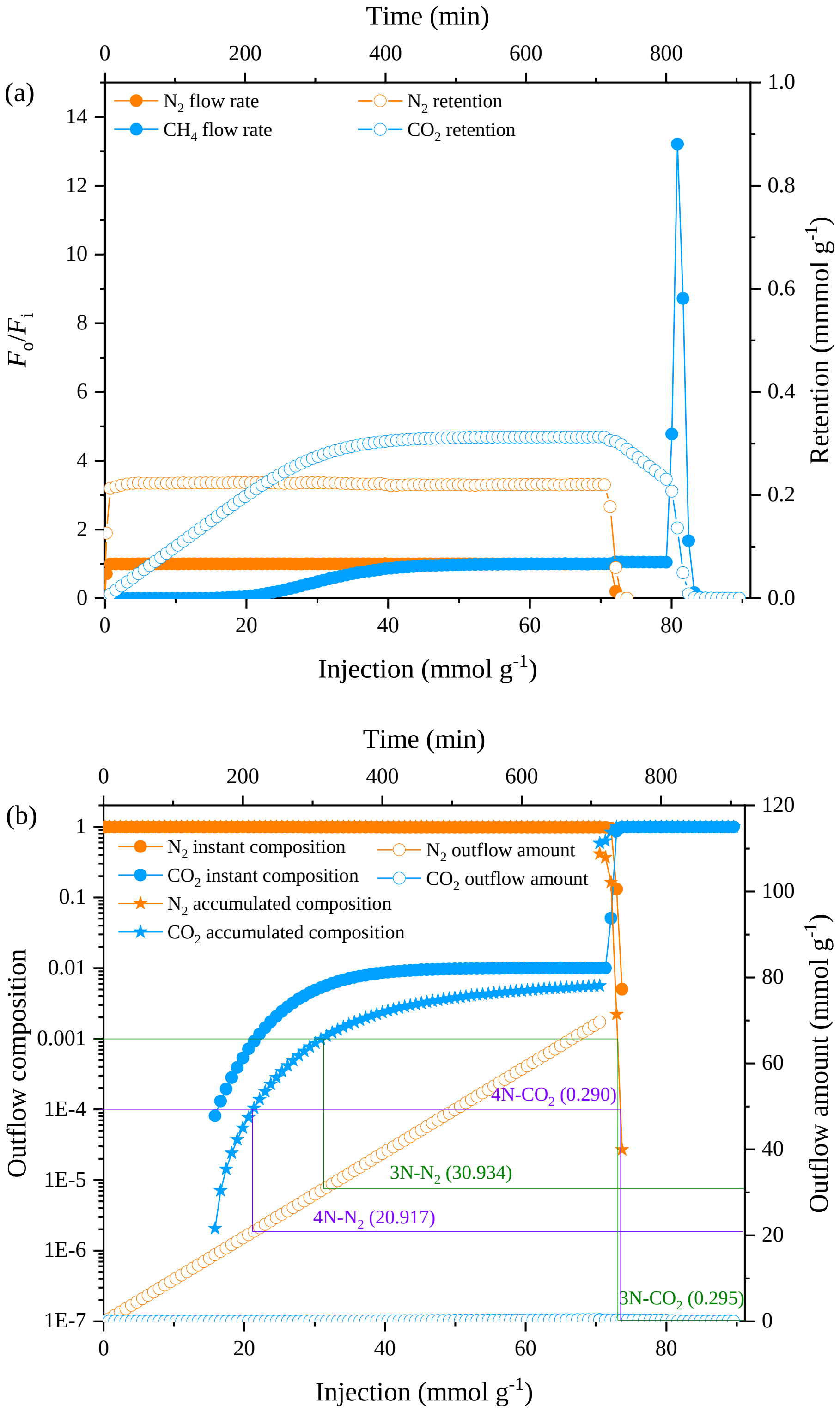


**Figure S66.** Detailed breakthrough curves of **M-*sp*** for a 1:99 $CO_2/N_2$ mixture at 298 K and ambient pressure (3rd measurement). (a) Breakthrough and retention curves. (b) Separation performances.

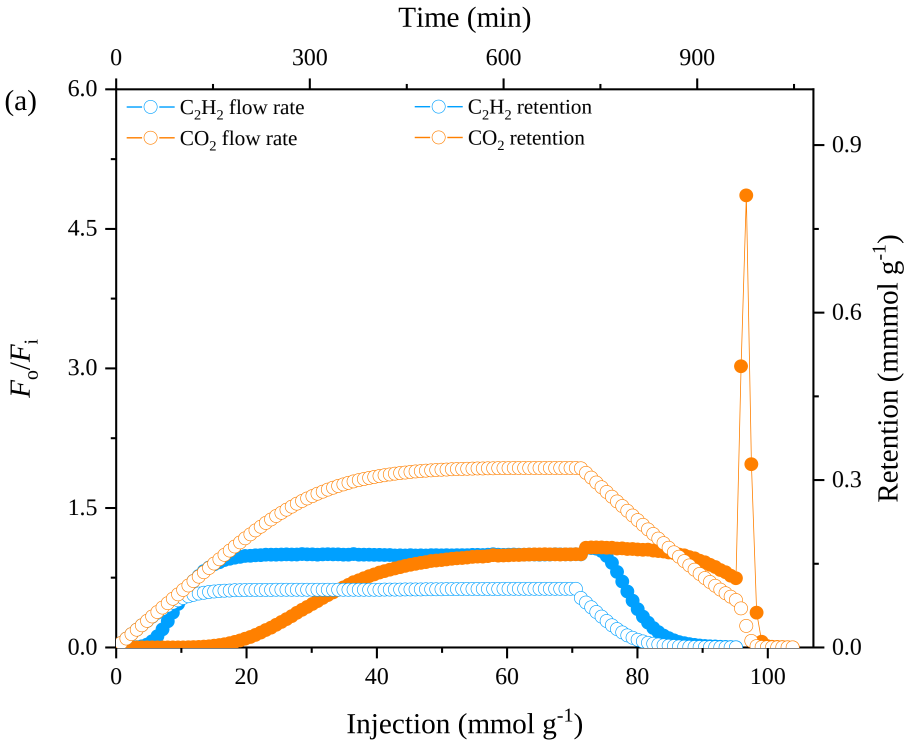


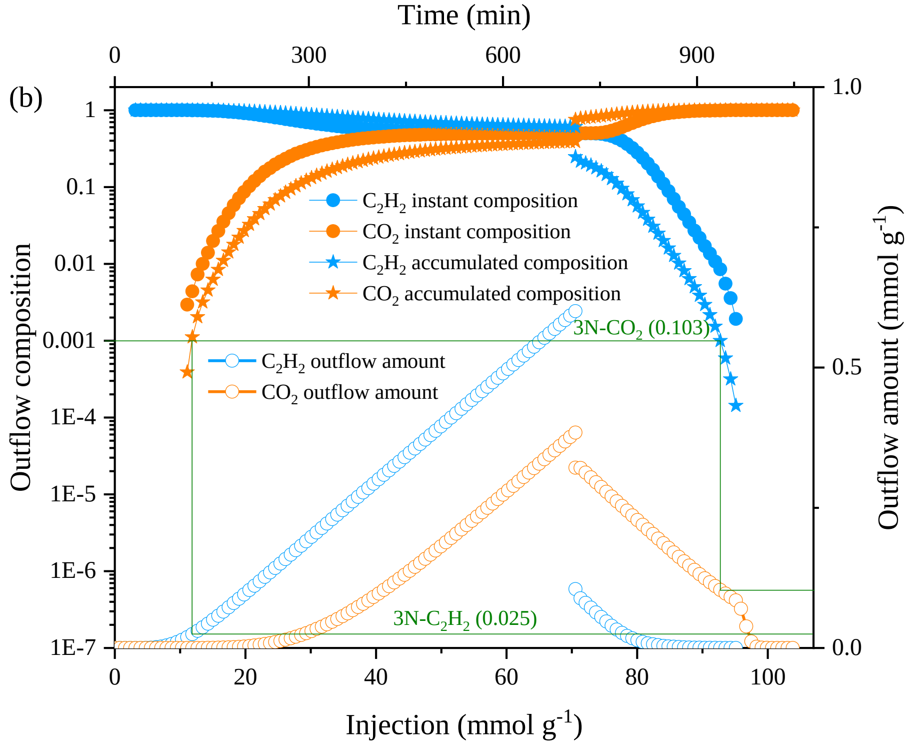


**Figure S67.** Detailed breakthrough curves of **M-*sp*** for a 1:1:98 $CO_2/C_2H_2$/Ar mixture at 298 K and ambient pressure (1st measurement). (a) Breakthrough and retention curves. (b) Separation performances.

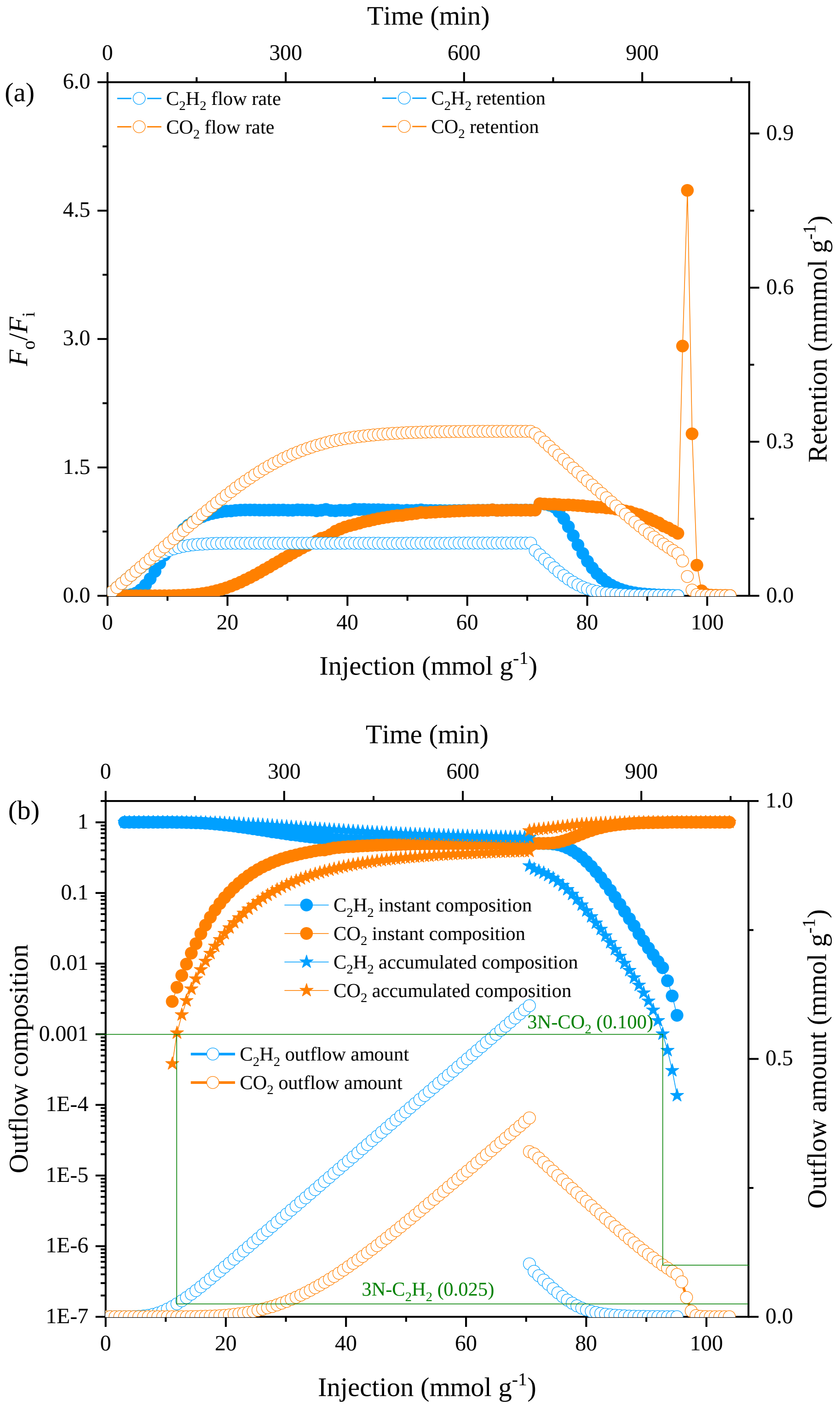


**Figure S68.** Detailed breakthrough curves of ***M-sp*** for a 1:1:98 $CO_2/C_2H_2/Ar$ mixture at 298 K and ambient pressure (2nd measurement). (a) Breakthrough and retention curves. (b) Separation performances.

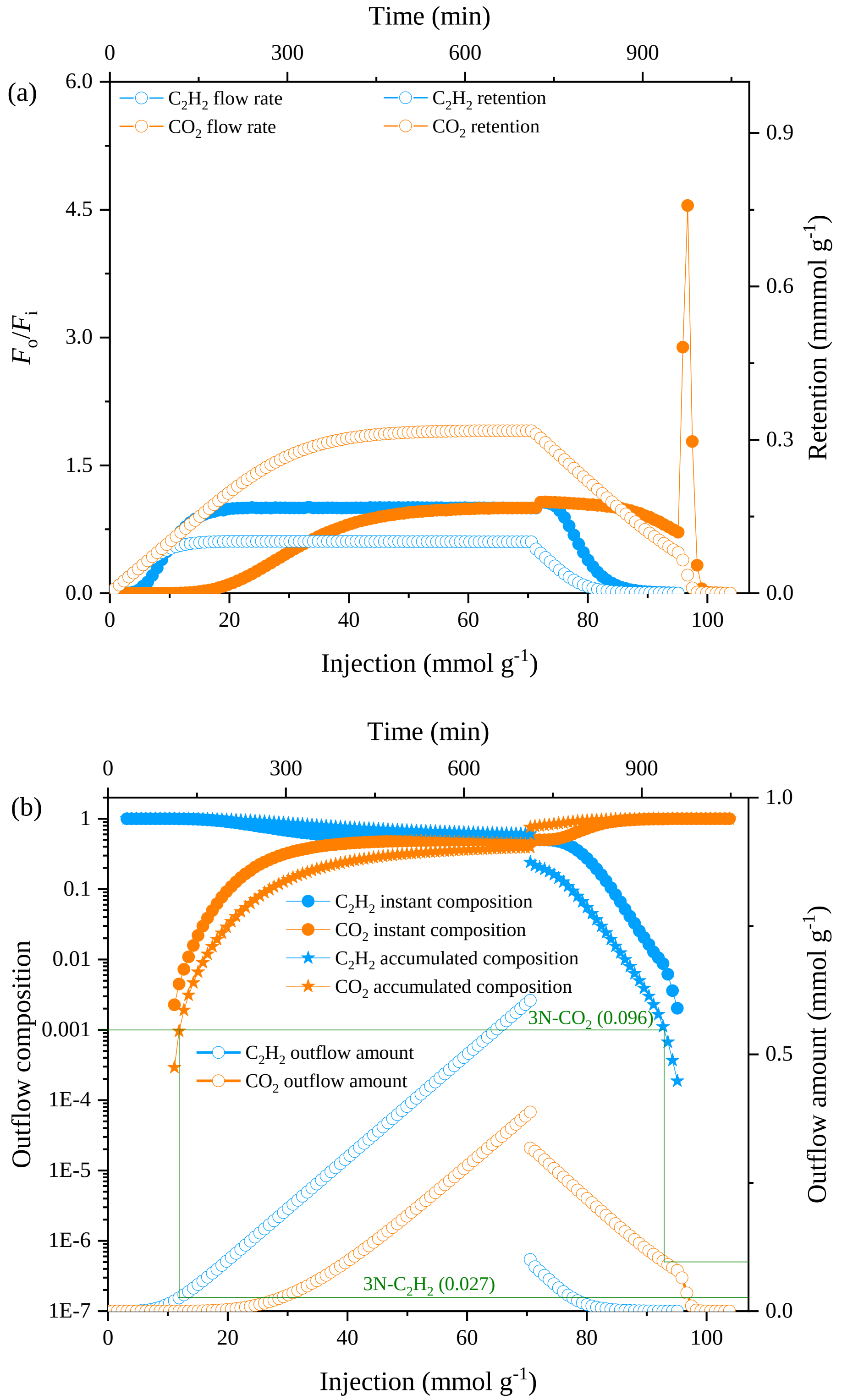


**Figure S69.** Detailed breakthrough curves of ***M-sp*** for a 1:1:98 $CO_2/C_2H_2/Ar$ mixture at 298 K and ambient pressure (3rd measurement). (a) Breakthrough and retention curves. (b) Separation performances.

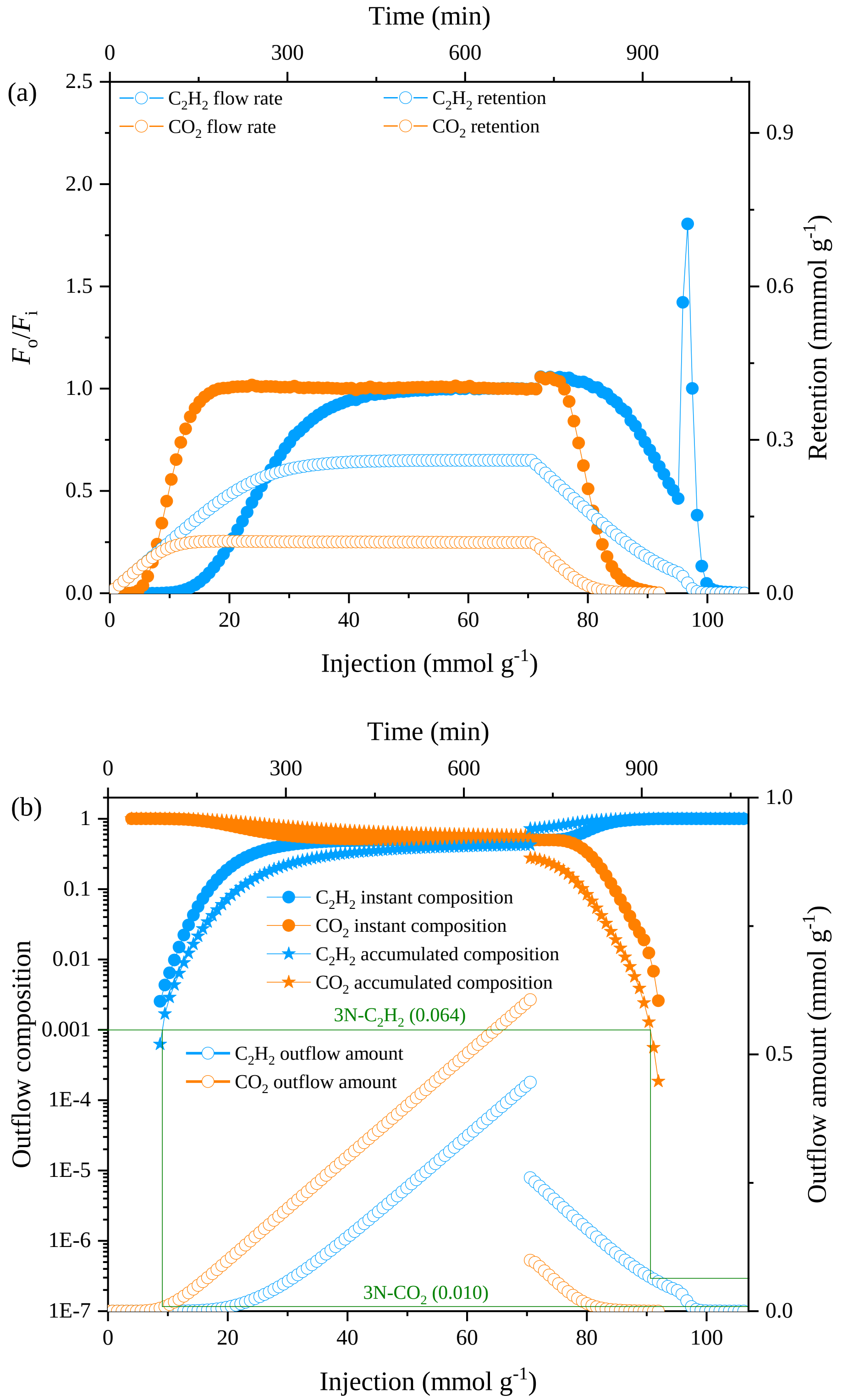


**Figure S70.** Detailed breakthrough curves of ***M-lp*** for a 1:1:98 $CO_2/C_2H_2/Ar$ mixture at 298 K and ambient pressure (1st measurement). (a) Breakthrough and retention curves. (b) Separation performances.

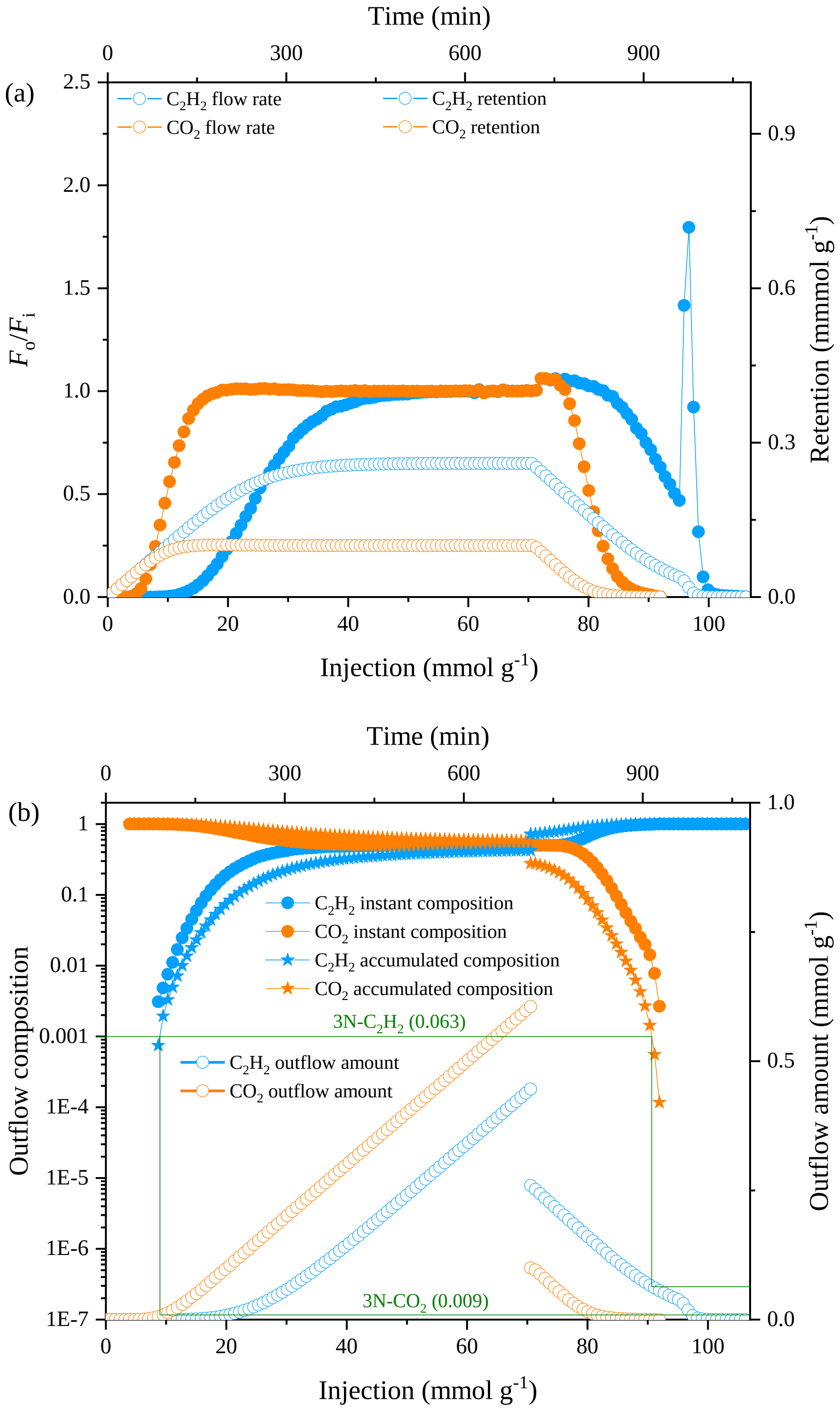


**Figure S71.** Detailed breakthrough curves of ***M-lp*** for a 1:1:98 $CO_2/C_2H_2/Ar$ mixture at 298 K and ambient pressure (2nd measurement). (a) Breakthrough and retention curves. (b) Separation performances.

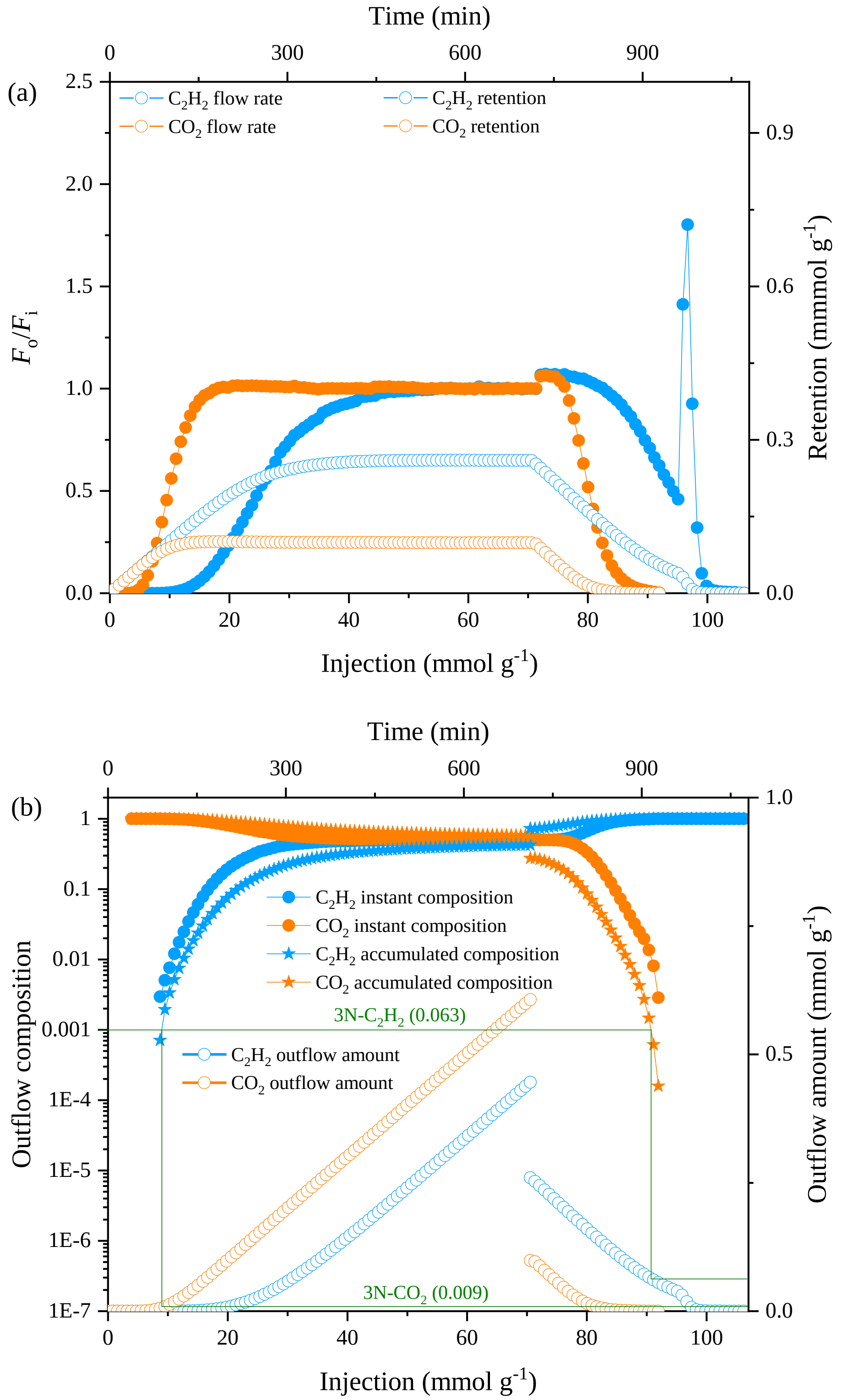


**Figure S72.** Detailed breakthrough curves of ***M-lp*** for a 1:1:98 $CO_2/C_2H_2/Ar$ mixture at 298 K and ambient pressure (3rd measurement). (a) Breakthrough and retention curves. (b) Separation performances.

**Table S1.** Key parameters and behaviors of different types of flexibility (for simplicity, only bistability with two energy states is considered).

| | $\Delta E$ | Driving force of thermodynamically non-spontaneous transformation | $E_a$ of thermodynamically spontaneous transformation | Stability of the guest-free high energy state | | Pathways from high to low energy state |
|---|---|---|---|---|---|---|
| | | | | Thermodynamics | Kinetics | |
| Dynamism (Gating) | ultrasmall | System energy fluctuation or adsorption/exchange | $<xkT$[a] | From unstable to stable when $\Delta E$ approaching 0 | Unstable | Spontaneous |
| Elasticity | not too small | Adsorption/Exchange | $<xkT$[a] | Unstable | Unstable | Spontaneous after desorption |
| Plasticity | As small as possible | | $\approx/>xkT$[a] | From unstable to stable when $\Delta E$ approaching 0 | Stable | Heating after desorption (**unnecessary**) or adsorption/exchange (**necessary, key behavior**) |
| Shape memory | not too small | | $\approx xkT$[a] | Unstable | Stable | Heating after desorption (**necessary, key behavior**) or adsorption/exchange[b] (**unnecessary**) |

[a] *xkT* refers to the system energy fluctuation, where *x* is related to the spatial range of independent structural transformation (e.g., the domain size).

[b] Because the low energy state is generally nonporous, these actions can be hardly used for pore shaping.

**Table S2.** Crystallographic data and structural refinement details.

| Compound | **M-*lp*** ·DMA | **M-*sp*** ·2$H_2O$ | **M-*lp*** | **M-*sp*** |
|---|---|---|---|---|
| Formula | $C_{12}H_{16}N_6O_7Zn_3$ | $C_8H_{11}N_5O_8Zn_3$ | $C_8H_7N_5O_6Zn_3$ | $C_8H_7N_5O_6Zn_3$ |
| Formula weight | 552.42 | 501.33 | 465.30 | 465.30 |
| Temperature (K) | 293(2) | 293(2) | 293(2) | 293(2) |
| Crystal system | orthorhombic | orthorhombic | orthorhombic | orthorhombic |
| Space group | $P2_12_12_1$ | $P2_12_12_1$ | $P2_12_12_1$ | $P2_12_12_1$ |
| $a$ / Å | 7.85176(6) | 7.78019(17) | 7.8902(4) | 7.79470(10) |
| $b$ / Å | 11.36087(9) | 11.1328(3) | 11.2677(4) | 11.06390(10) |
| $c$ / Å | 20.7770(2) | 18.1295(6) | 20.5775(8) | 18.1671(3) |
| $V$ / Å$^3$ | 1853.37(3) | 1570.30(7) | 1829.43(13) | 1566.73(4) |
| $Z$ | 4 | 4 | 4 | 4 |
| $D_c$ / g cm$^{-3}$ | 1.980 | 2.121 | 1.689 | 1.973 |
| Flack/Basf[a] | 0.03(2) | 0.01(3) | 0.14(16)[a] | 0.23(4)[a] |
| $R_{int}$ | 0.0194 | 0.0707 | 0.0945 | 0.0434 |
| $R_1$ [$I > 2\sigma(I)$] | 0.0262 | 0.0413 | 0.0659 | 0.0290 |
| $wR_2$ (all data) | 0.0698 | 0.1072 | 0.2530 | 0.0789 |
| GOF | 1.070 | 1.062 | 1.075 | 1.049 |

| Compound | **A-*sp*** ·2$H_2O$ | **A-*lp*** ·5$H_2O$ | **H-*sp*** ·2$H_2O$ | **H-*lp*** ·5$H_2O$ |
|---|---|---|---|---|
| Formula | $C_7H_{10}N_6O_8Zn_3$ | $C_7H_{16}N_6O_{11}Zn_3$ | $C_7H_9N_5O_8Zn_3$ | $C_7H_{15}N_5O_{11}Zn_3$ |
| Formula weight | 502.32 | 556.40 | 487.35 | 541.39 |
| Temperature (K) | 293(2) | 293(2) | 293(2) | 293(2) |
| Crystal system | orthorhombic | orthorhombic | orthorhombic | orthorhombic |
| Space group | $P2_12_12_1$ | $P2_12_12_1$ | $P2_12_12_1$ | $P2_12_12_1$ |
| $a$ / Å | 7.5309(2) | 7.717(1) | 7.655(2) | 7.725(1) |
| $b$ / Å | 10.7642(3) | 11.391(1) | 10.879(2) | 11.262(1) |
| $c$ / Å | 17.9702(4) | 20.883(1) | 18.738(5) | 21.311(1) |
| $V$ / Å$^3$ | 1456.74(6) | 1836(1) | 1560(1) | 1854(1) |
| $Z$ | 4 | 4 | 4 | 4 |
| $D_c$ / g cm$^{-3}$ | 2.290 | 2.013 | 2.075 | 1.940 |
| Flack/Basf[a] | 0.25(5)[a] | / | / | / |
| $R_{int}$ | 0.0276 | / | / | / |
| $R_1$ [$I > 2\sigma(I)$] | 0.0263 | $R_p$ = 0.0182 | $R_p$ = 0.0455 | $R_p$ = 0.0432 |
| $wR_2$ (all data) | 0.0723 | $R_{wp}$ = 0.0238 | $R_{wp}$ = 0.0755 | $R_{wp}$ = 0.0581 |
| GOF | 1.067 | / | / | / |

[a] Twin law [-1 0 0 0 -1 0 0 0 -1]

**Table S3.** Comparison of framework energies.

| | Energy (hartree/unit cell) | | |
|---|---|---|---|
| | *sp* | *lp* | Δ |
| **H** | -7030.9603887 | **-7030.9989993** | -0.0386106 |
| **A** | **-7252.3942787** | -7252.3639547 | 0.0303240 |
| **M** | -7188.113059 | **-7188.1169896** | 0.0039306 |

1 hartree = 2625.5 kJ $mol^{-1}$; $Z = 4$

**Table S4.** Comparison of the 20:80 $CH_4/N_2$ mixture column breakthrough separation performances.

| | | **M-*sp*** | | **M-*lp*** | |
|---|---|---|---|---|---|
| | | $CH_4$ | $N_2$ | $CH_4$ | $N_2$ |
| Retention (mmol $g^{-1}$) | 1st | 0.094 | 0.177 | 0.321 | 0.218 |
| | 2nd | 0.093 | 0.175 | 0.319 | 0.221 |
| | 3rd | 0.095 | 0.175 | 0.322 | 0.222 |
| | Average | 0.094(1) | 0.176(1) | 0.321(2) | 0.220(2) |
| Dead space (mmol $g^{-1}$) | | 0.183(4) | | | |
| Uptake (mmol $g^{-1}$) | | 0.057(5) | 0.029(5) | 0.284(6) | 0.074(6) |
| Single-component isotherm (mmol $g^{-1}$) | | 0.10 | 0.08 | 0.32 | 0.24 |
| Experimental selectivity | | 7.8 | | 15.4 | |
| 3N Productivity (mmol $g^{-1}$) | 1st | 0.018 | 0 | 0.179 | 0.432 |
| | 2nd | 0.019 | 0 | 0.181 | 0.435 |
| | 3rd | 0.020 | 0 | 0.181 | 0.437 |
| | Average | 0.019(1) | 0 | 0.180(1) | 0.435(3) |
| 4N Productivity (mmol $g^{-1}$) | 1st | N.A. | 0 | 0.154 | 0.272 |
| | 2nd | N.A. | 0 | 0.149 | 0.269 |
| | 3rd | N.A. | 0 | 0.152 | 0.269 |
| | Average | N.A. | 0 | 0.152(3) | 0.270(2) |

N.A. = Not applicable

**Table S5.** Comparison of the 1:99 $CO_2/N_2$ mixture column breakthrough separation performances.

| | | **M-*sp*** | | **M-*lp*** | |
|---|---|---|---|---|---|
| | | $CO_2$ | $N_2$ | $CO_2$ | $N_2$ |
| Retention (mmol $g^{-1}$) | 1st | 0.313 | 0.221 | 0.095 | 0.264 |
| | 2nd | 0.312 | 0.222 | 0.094 | 0.265 |
| | 3rd | 0.312 | 0.220 | 0.095 | 0.266 |
| | Average | 0.312(1) | 0.221(1) | 0.095(1) | 0.265(1) |
| Dead space (mmol $g^{-1}$) | | 0.183(4) | | | |
| Uptake (mmol $g^{-1}$) | | 0.311(5) | 0.040(5) | 0.093(5) | 0.084(5) |
| Single-component isotherm (mmol $g^{-1}$) | | 0.36 | 0.11 | 0.11 | 0.28 |
| Experimental selectivity | | 772 | | 110 | |
| 3N Productivity (mmol $g^{-1}$) | 1st | 0.074 | 8.376 | 0.298 | 30.968 |
| | 2nd | 0.075 | 8.432 | 0.296 | 30.934 |
| | 3rd | 0.074 | 8.418 | 0.295 | 30.934 |
| | Average | 0.074(1) | 8.409(29) | 0.296(2) | 30.945(20) |
| 4N Productivity (mmol $g^{-1}$) | 1st | N.A. | 5.348 | 0.293 | 20.951 |
| | 2nd | N.A. | 5.362 | 0.291 | 20.917 |
| | 3rd | N.A. | 5.334 | 0.290 | 20.917 |
| | Average | N.A. | 5.348(14) | 0.291(2) | 20.928(20) |

N.A. = Not applicable

**Table S6.** Comparison of the 1:1:98 $CO_2/C_2H_2/Ar$ mixture column breakthrough separation performances.

| | | **M-*sp*** | | **M-*lp*** | |
|---|---|---|---|---|---|
| | | $CO_2$ | $C_2H_2$ | $CO_2$ | $C_2H_2$ |
| Retention (mmol $g^{-1}$) | 1st | 0.321 | 0.105 | 0.099 | 0.260 |
| | 2nd | 0.320 | 0.102 | 0.100 | 0.259 |
| | 3rd | 0.318 | 0.101 | 0.099 | 0.260 |
| | Average | 0.320(2) | 0.103(2) | 0.099(1) | 0.260(1) |
| Dead space (mmol $g^{-1}$) | | 0.183(4) | | | |
| Uptake (mmol $g^{-1}$) | | 0.318(6) | 0.101(6) | 0.098(5) | 0.258(5) |
| Single-component isotherm (mmol $g^{-1}$) | | 0.36 | 0.11 | 0.11 | 0.27 |
| Experimental selectivity | | 3.2 | | 2.6 | |
| 3N Productivity (mmol $g^{-1}$) | 1st | 0.103 | 0.025 | 0.010 | 0.064 |
| | 2nd | 0.100 | 0.025 | 0.009 | 0.063 |
| | 3rd | 0.096 | 0.027 | 0.009 | 0.063 |
| | Average | 0.100(4) | 0.026(1) | 0.009(1) | 0.063(1) |
